# Mechanochemistry Enables Rapid and Solvent‐Free Wittig Reactions on Sugars

**DOI:** 10.1002/cssc.202502026

**Published:** 2025-12-16

**Authors:** Francesco Mele, Nina Biedermann, Christoph Suster, Johanna Templ, Christian Stanetty, Michael Schnürch

**Affiliations:** ^1^ Institute of Applied Synthetic Chemistry TU Wien Vienna Austria; ^2^ SynCat Lab Department of Chemistry, Life Sciences and Environmental Sustainability University of Parma Parma Italy

**Keywords:** ball milling, carbohydrate, mechanochemistry, Wittig reaction

## Abstract

A versatile solvent‐free Wittig olefination of sugars via mechanochemistry is reported, tolerating common protecting groups as well as unprotected substrates. The method efficiently delivers structurally diverse olefins under mild and solvent‐free conditions. Subsequent solvent‐free late‐stage modifications of the olefins demonstrate the potential of the methodology for greener sugar‐based synthetic sequences.

## Graphical abstract

A solvent‐free mechanochemical protocol for the Wittig olefination of reducing sugars is reported. The method enables rapid access to structurally diverse olefins from acetonide‐ and benzyl‐protected carbohydrates, as well as unprotected sugars, under mild conditions, with improved selectivity, broad substrate scope, and enhanced sustainability.

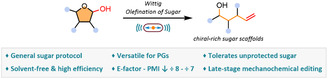

## 1 Introduction

Carbohydrates—nature's most abundant chiral feedstock—offer a renewable source of densely functionalized, chiral molecules. Their array of stereogenic centers together with the ability to form oligomeric or polymeric structures is the basis of countless important functions in biological systems [1, 2]. They are, in principle, ideal “ex‐chiral‐pool” feedstocks for green total synthesis [3, 4]. In many synthetic campaigns, a key step is the ring‐opening of the cyclic hemiacetal by targeting the aldehyde moiety [5, 6, 7].

**FIGURE 1:**
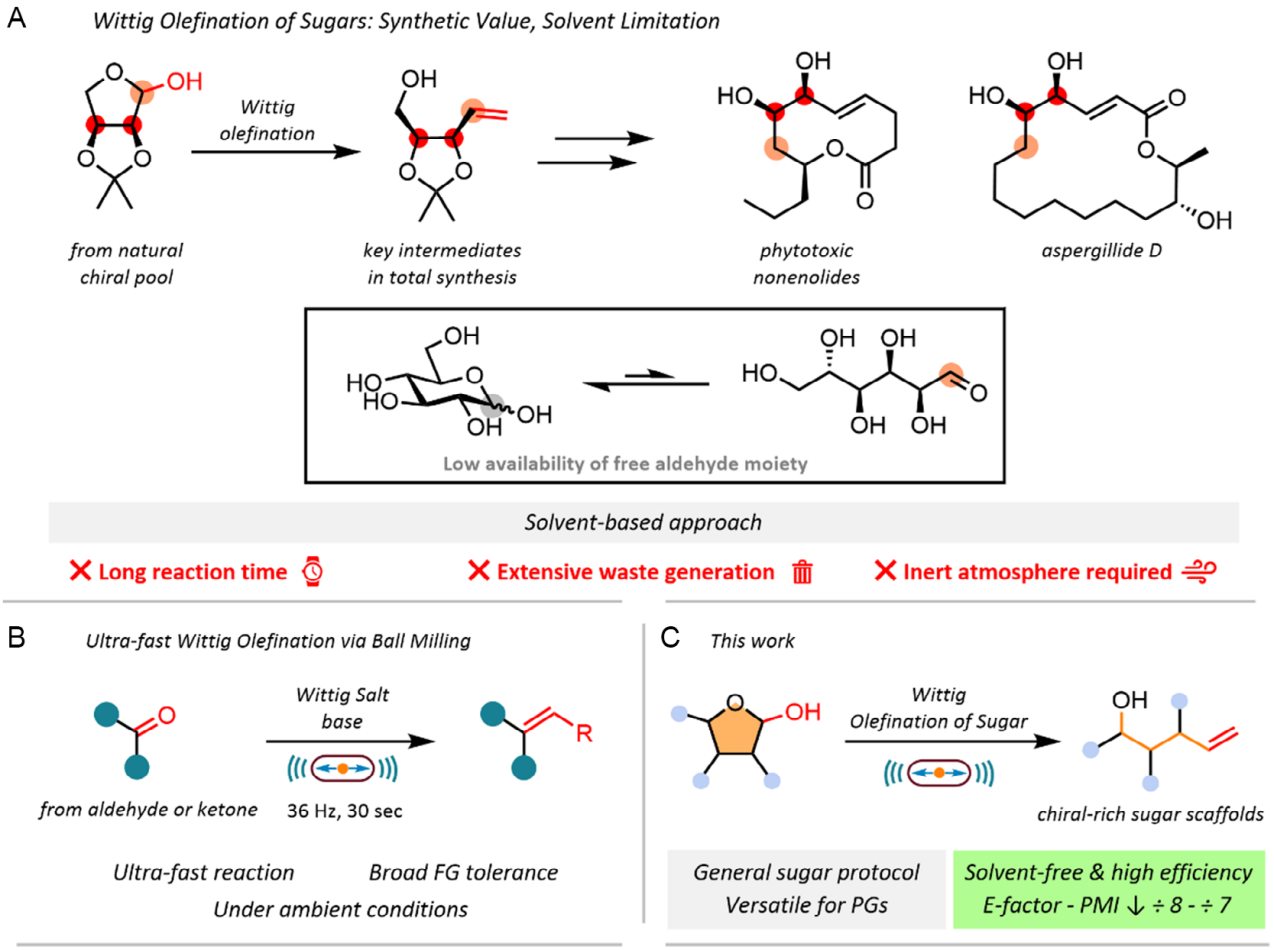
(A) Synthetic relevance and current limitations of the Wittig olefination on sugars: examples of its application in total synthesis and drawbacks of typical solution‐based protocols. (B) Our previous study on the mechanochemical Wittig reaction of common aldehydes and ketones. (C) This work.

Such reactions present a big challenge, as only minute amounts of aldehyde are exposed to react with common sugar molecules [8, 9], rendering direct functionalization kinetically unfavored. Examples of such transformations often introduce transient protecting groups such as oximes [5, 10] and thioacetals [11], or directly use C—C bond‐forming reactions such as indium‐mediated acyloxyallylation [12, 13], Grignard‐type reactions [14], and others. For example, the Wittig reaction (and its variants) has been exploited to give direct access to substituted alkene species or Michael acceptors [15, 16, 17]. The method was shown to perform well on a wide range of carbohydrates, in both protected and unprotected form. However, traditional protocols generally suffer from long reaction times, high temperatures, and low energy efficiency and significant solvent consumption (Figure 1A).

In this light, mechanochemistry has emerged as a powerful and versatile platform in modern synthetic chemistry, offering a promising alternative to traditional solution‐based methodologies [18, 19, 20, 21]. Beyond providing operational simplicity and access to unique reaction environments, mechanochemical methods often exhibit faster kinetics [22, 23, 24, 25], enhanced selectivity [26, 27, 28, 29], and dramatically reduced solvent requirements [30, 31, 32, 33]. These features make it particularly attractive for the development of more sustainable synthetic processes [34, 35], while also enabling transformations that remain challenging or entirely inaccessible in solution [36, 37]. Specifically for the Wittig olefination on general substrates, we recently demonstrated that a lot of the drawbacks of solution‐based protocols can be overcome by employing mechanical agitation instead of conventional solvent‐based conditions for the reaction [38]. Utilizing a solvent‐free ball milling approach allowed us to perform Wittig reactions of conventional ketones and aldehydes within seconds instead of hours (Figure 1B).

Encouraged by these findings, we were interested in whether the excellent reaction kinetics of these mechanochemical conditions allow transformations on the anomeric center of carbohydrates under mild conditions. To date, mechanochemistry has shown promise in carbohydrate chemistry—for monosaccharides particularly in glycosylation reactions (*O*‐,*N*‐,*S*‐glycosylation) [39, 40, 41, 42] and azide formation [43], modifications of the ribose or deoxyribose unit in nucleosides [44], or glycosylation toward disaccharides [45, 46]—but examples targeting the masked aldehyde functionality remain unexplored. Developing such a strategy would not only broaden the scope of mechanochemical transformations but also provide a direct and sustainable route to alkene‐functionalized sugar‐derivatives.

Herein, we present a mechanochemical protocol for the Wittig olefination of reducing sugar derivatives under solvent‐free conditions using ball milling. The method is applicable to a variety of monosaccharides bearing different protecting groups and is designed to overcome key challenges of traditional protocols, such as long reaction times, limited operational simplicity, and extensive use of organic solvents. By integrating solvent‐free conditions with a solid‐state activation strategy, this approach aims to provide a more sustainable alternative for carbohydrate functionalization.

## 2 Results and Discussion

### 2.1 Optimization of the Reaction

We commenced our investigation using a commercially available d‐mannose derivative (**1a**) with a free hydroxyl group at the anomeric center and two acetonide protecting groups as model substrate. To identify the optimal conditions for the formation of olefin **2a**, we employed methyltriphenylphosphonium bromide (PPh_3_MeBr) as the Wittig methenylation reagent and screened a series of bases. Reactions were performed in a PTFE milling vessel (8.2 mL inner volume) charged with a single stainless‐steel ball (ø = 12 mm), and in all cases, preformation of the reactive ylide species by milling of Wittig salt and base was conducted prior to substrate addition.

To assess the feasibility of the reaction, we first tested the process under completely solvent‐free (*neat*) conditions by milling the reactants without any liquid additive with KO^*t*^Bu as base. Remarkably, these conditions proved highly efficient, affording the desired olefin **2a** in 92% yield after 1 h of milling at 30 Hz (Table 1, entry 1).

**TABLE 1:** Optimization of reaction conditions.

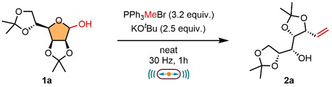		
Entry	Deviation from conditions	**Yield (%) of 2a** a
1	None	92
2	Toluene as LAG	85
3	^*t*^BuOH as LAG	52
4	THF as LAG	91
5	KO^*t*^Bu (4 equiv.), P Salt (5 equiv.)	94
6	KO^*t*^Bu (1.2 equiv.), P Salt (1.5 equiv.)	46
7	Cs_2_CO_3_ as base	66
8	DBU as base	—
9	NaHMDS as base	73
10	ZrO_2_ milling ball (Ø = 12 mm)	87
11	20 Hz	51
**12**	**35 Hz**	**96**

*General procedure*: In a PTFE milling vessel (*V* = 8.2 mL) equipped with a single stainless‐steel ball (ø = 12 mm), PPh_3_MeBr (0.64 mmol), and KO^*t*^Bu (0.50 mmol) are added sequentially. The vessel is sealed, mounted on a ball mill, and shaken at a frequency of 30 Hz for 1 min. Subsequently, substrate **1a** (0.2 mmol) and the LAG agent (when specified) are added, and milling is continued for 1 h.

a Yields determined by ^1^H‐NMR spectroscopy (3,4,5‐trichloropyridine as internal standard).

Subsequently, we explored whether the use of a liquid‐assisted grinding (LAG) agent could further influence the reaction outcome. An initial test with toluene—a typical solvent for Wittig reactions—used as LAG additive (150 μL, *η* = 0.5) provided **2a** in 85% yield (entry 2). To better understand the effect of the additive's nature, we also tested two moderately polar solvents: *tert*‐butanol (^*t*^BuOH), a protic additive, and tetrahydrofuran (THF), an aprotic one (entries 3 and 4). It is noteworthy that ^*t*^BuOH is inherently formed in situ during the acid–base reaction involved in ylide formation (approximately 50 μL, *η* = 0.14). However, its further addition negatively affected the outcome, lowering the yield to 52%. In contrast, THF delivered **2a** in 91% yield, comparable to the result obtained under neat conditions. Based on these results, neat conditions were selected for further studies, offering both optimal efficiency and full compliance with a solvent‐free methodology.

Proceeding with our investigations, we evaluated the effect of various amounts of phosphonium salt and base while keeping their ratio constant. An increase in their quantities led to a modest improvement in yield (94%, entry 5), whereas a reduction caused a significant drop to 46% (entry 6). Subsequently, other bases were tested (see Section A.1.3 of the Supporting Information for full details), revealing that under neat conditions, Cs_2_CO_3_ delivered **2a** in 66% yield (entry 7), proceeding with comparable efficiency to KO^*t*^Bu, contrary to our previous observations for common aldehydes, where Cs_2_CO_3_ was only effective under LAG conditions [38]. Stronger bases such as DBU and solid NaHMDS were evaluated as well (entry 8 and entry 9). Interestingly, liquid DBU completely suppressed the reaction, whereas NaHMDS provided a satisfactory yield of 73%. Replacing the stainless‐steel ball with a ZrO_2_ grinding ball—to rule out any effect of the metallic grinding media—still afforded product **2a** in a consistent yield of 87% (entry 10). Finally, the milling frequency was reduced to 20 Hz resulting in a significantly lower yield of 51% (entry 11), while milling at 35 Hz resulted in further improvement compared to 30 Hz, with the product **2a** being obtained in 96% yield (entry 12). These results demonstrate that efficient mechanochemical activation—which depends on the milling frequency—is essential for achieving the desired solvent‐free transformation.

### 2.2 Investigation of the Reaction Scope

**SCHEME 1:**
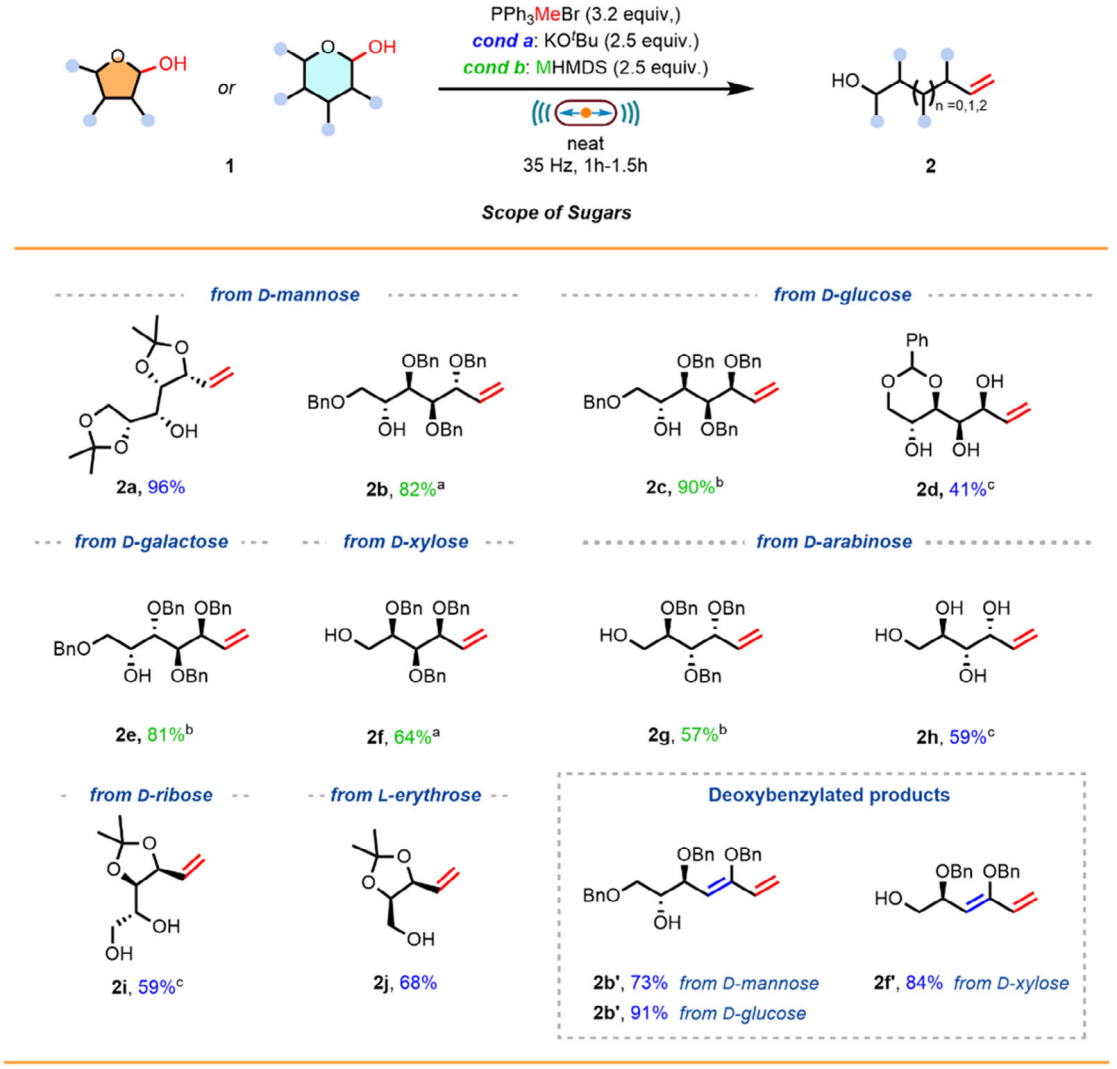
Scope of the Wittig olefination of sugars. Reactions were carried out on a 0.2‐mmol scale under air, using a PTFE milling jar (8.2 mL) and one hardened stainless‐steel milling ball (ø = 12 mm) at a milling frequency of 35 Hz until complete consumption of the starting material was observed. *Conditions a*: for acetonide‐protected sugars, the reaction was performed using KO^*t*^Bu as base (0.5 mmol, 2.5 equiv.) and PPh_3_MeBr (0.64 mmol, 3.2 equiv.). *Conditions b*: for benzyl‐protected sugars, the reaction was performed using LiHMDS or NaHMDS as base (0.5 mmol, 2.5 equiv.) and PPh_3_MeBr (0.64 mmol, 3.2 equiv.). All reported yields are intended after isolation. ^a^LiHMDS was used as base. ^b^NaHMDS was used as base. ^c^Conditions were adjusted to account for free OH groups, adding one additional equivalent of the generated ylide per free OH present in the starting material.

Having established the optimal solvent‐free conditions for the Wittig olefination of sugars, we next explored the scope of the reaction on a series of substrates (Scheme 1). We began our study with a benzyl‐protected d‐mannose derivative **1b**. It was discovered that under the optimal conditions using KO^*t*^Bu as base, the reaction selectively afforded the conjugated diene **2b′** in 73% yield through a competing pathway in which methylenation occurred concurrently with the elimination of benzyl alcohol (Scheme 1 bottom right and Supporting Information, Section A.2.1 for detailed studies). This behavior is consistent with previous reports describing similar reactivity of benzyl‐protected hexoses under solution‐phase conditions [47]. Therefore, other bases were investigated, revealing that the use of LiHMDS efficiently suppressed the elimination and delivered the desired compound **2b** in 82% yield, which is also in agreement with Sawant et al.'s work [47], who attributed the different selectivity to the steric hindrance of the base employed. Based on this outcome, *conditions a* (*cond a*: KO^*t*^Bu, 2.5 equiv.; PPh_3_MeBr, 3.2 equiv.; 35 Hz, 1.5 h) were applied to acetal‐protected sugars, while *conditions b* (*cond b*: LiHMDS or NaHMDS, 2.5 equiv.; PPh_3_MeBr, 3.2 equiv.; 35 Hz, 1.5 h) were used for benzyl‐protected ones. By adapting the reaction conditions accordingly, the benzyl‐protected d‐glucose derivative **2c** was obtained in excellent yield (90%) under *cond b* using NaHMDS as base (see Supporting Information, Section A.2.2 for the selection of the base). Conversely, applying *cond a*, again the elimination of benzyl alcohol described previously was observed, furnishing the conjugated diene **2b′** in excellent yield (91%) and with high selectivity. We also investigated a partially protected d‐glucose derivative featuring a benzylidene acetal. Interestingly, the desired product **2d** was obtained under solvent‐free conditions in a synthetically useful yield of 41%, by simply increasing the equivalents of the generated ylide to match the number of free hydroxyl groups present on the substrate (KO^*t*^Bu, 4.5 equiv.; PPh_3_MeBr, 5.2 equiv.). Pleasingly, when benzyl‐protected d‐galactose was subjected to *cond b*, the corresponding Wittig product **2e** was obtained in an excellent yield of 81%.

Next, we extended our investigations to a series of aldo‐pentose sugars. d‐Xylose, which shares the same stereochemistry at C‐2, C‐3, and C‐4 as d‐glucose, showed a similar behavior under *cond a*, providing the corresponding diene **2f′** in 84% yield (see Supporting Information, Section A.2.4). Switching to *cond b*, the Wittig product **2f** was obtained in good yield (64%) with complete selectivity for the desired product and residual starting material, reflecting effective control over the selectivity of the methodology. Pleasingly, the d‐arabinose‐derived product **2g** was obtained in a synthetically useful yield of 57% under *cond b*. Importantly, when unprotected d‐arabinose was employed, the desired product **2h** was obtained in good yield (59%) without the need for protection. Similarly, the acetonide‐protected d‐ribose, bearing a free OH group, was well tolerated under *cond a*, affording product **2i** with a satisfactory yield of 59%. Finally, the compatibility of the mechanochemical conditions was evaluated by acetonide‐protected l‐erythrose, a tetrose monosaccharide. This substrate is of particular interest due to its limited number of stereocenters, making it a useful intermediate in total synthesis [48, 49, 50]. Under *cond a*, the synthetically valuable product **2j** was obtained under mild, solvent‐free conditions in a useful yield of 68%.

**SCHEME 2:**
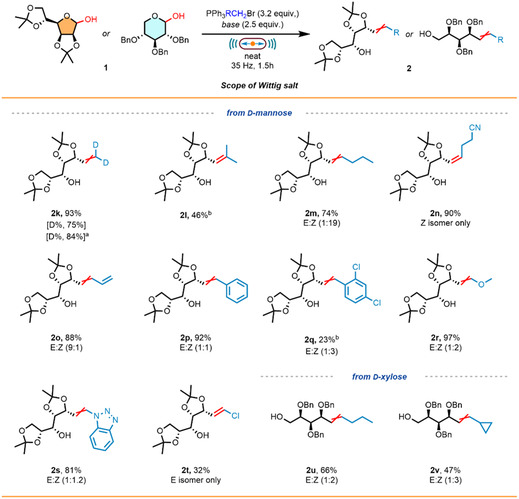
Scope of the Wittig salts. Reactions were carried out on a 0.2‐mmol scale under air, using a PTFE milling jar (8.2 mL) and one hardened stainless‐steel milling ball (Ø = 12 mm) at a milling frequency of 35 Hz and milling for 1.5 h. *Conditions for d‐mannose derivatives*: the reaction was performed using KO^*t*^Bu as base (0.5 mmol, 2.5 equiv.) and the corresponding Wittig salt (0.64 mmol, 3.2 equiv.). *Conditions for d‐xylose derivatives*: the reaction was performed using LiHMDS as base (0.5 mmol, 2.5 equiv.) and the corresponding Wittig salt (0.64 mmol, 3.2 equiv.). All reported yields are intended after isolation. ^a^Starting material **1a** was deuterated prior to milling. ^b^NaHMDS was used as base.

With the methodology established across a range of sugars and protecting groups, we examined its generality with respect to different Wittig salts to introduce structural diversity to the olefin products. This study was conducted using derivatives of d‐mannose **1a** as a model substrate, as well as a benzyl‐protected d‐xylose derivative (Scheme 2). When the deuterated Wittig salt (PPh_3_CD_3_Br) was employed, the corresponding product **2k** was obtained with comparable efficiency (93%) and a deuterium incorporation of 75%. This value could be further improved to 85% by subjecting substrate **1a** to a simple H/D exchange prior to the reaction using CD_3_OD. In contrast, when a Wittig salt bearing two substituents on the ylide carbon was used, the reaction proved inefficient using KO^*t*^Bu, likely due to the reduced acidity of the proton in *α*‐position to phosphorus in the Wittig salt. Nonetheless, the trisubstituted olefin **2l** could be accessed in a synthetically useful yield (46%) by switching to the stronger base NaHMDS. Employing a Wittig salt bearing a longer alkyl chain, the reaction proceeded efficiently, affording product **2m** in 74% yield. Interestingly, incorporation of a cyano group on the alkyl chain led to the formation of functionalized olefin **2n** in excellent yield (90%) exclusively as the Z‐isomer. Additionally, an allyl fragment was successfully installed, delivering the conjugated diene **2o** in an excellent yield of 88%. Moving on to aromatic substituents, the conjugated olefin **2p** bearing a phenyl ring was obtained with high efficiency and 92% yield. However, when a more electron‐deficient aryl group bearing two chlorine substituents was employed, the reaction proved significantly less efficient, affording product **2q** in only 23% yield (using NaHMDS). This decrease is likely due to stabilization of the ylide, resulting in diminished nucleophilicity. Furthermore, heteroatoms directly attached to the double bond, such as oxygen or nitrogen, were fully tolerated under solvent‐free conditions, affording products **2r** and **2s** in yields of 97% and 81%, respectively. Moreover, a chlorine atom could also be directly installed on the double bond, affording product **2t**, albeit with reduced efficiency (32% yield for the E‐isomer). Finally, d‐xylose derivatives, which required the use of a stronger base (LiHMDS), afforded products **2u** and **2v** bearing alkyl side chains in overall yields of 66% and 47%, respectively, with unreacted starting material recoverable from the reaction mixture.

### 2.3 Investigation of the Reaction Process and Late‐Stage Modification

**FIGURE 2:**
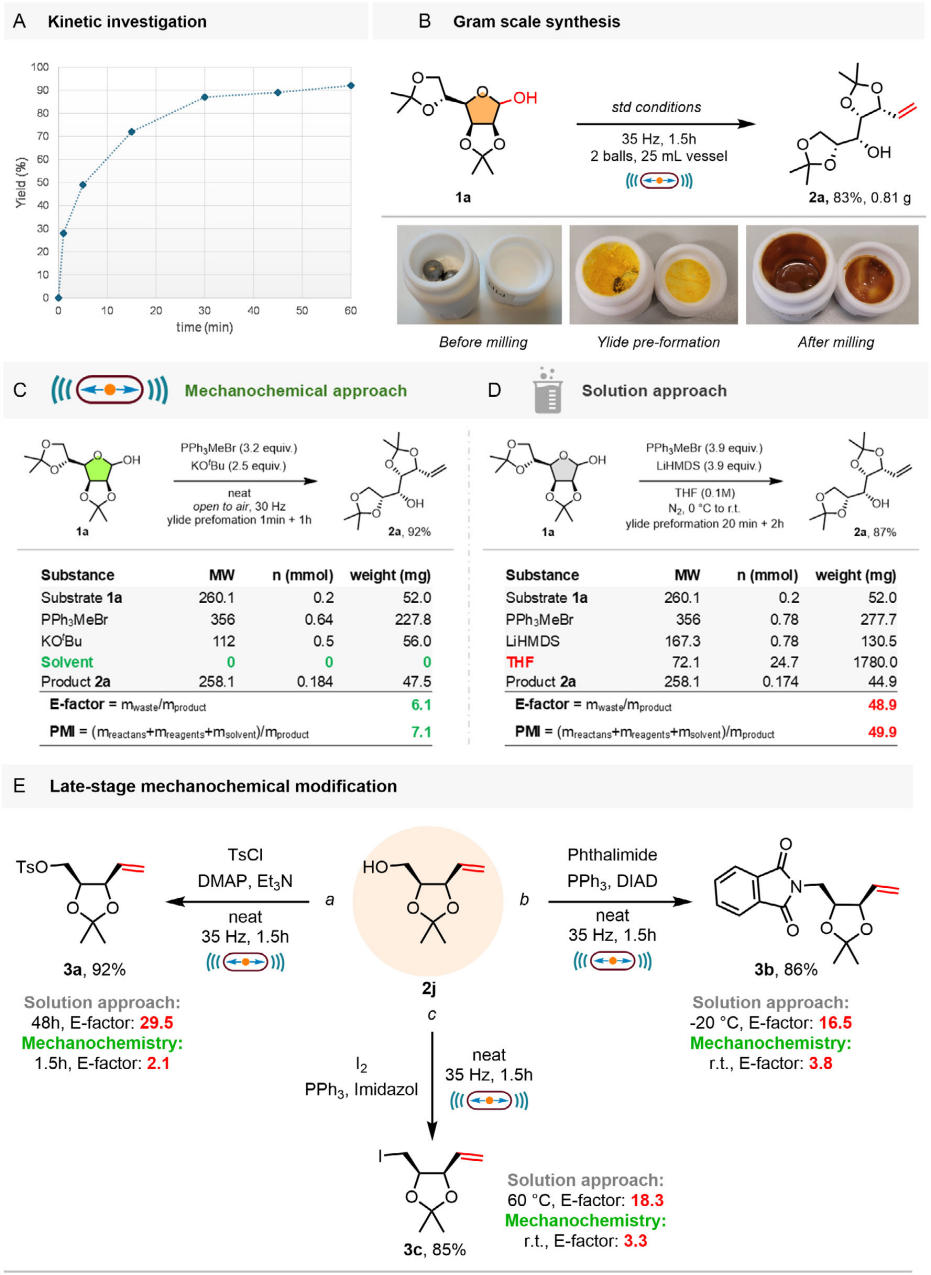
(A) Kinetic study of the mechanochemical Wittig methylenation reaction of sugar **1a**. (B) Gram‐scale mechanosynthesis of **2a**. (C) Evaluation of green chemistry metrics for our mechanochemical protocol and (D) previously reported solution‐based method. (E) Mechanochemical functionalizations of **2j**: (a) Tosyl‐protection; (b) Mitsunobu reaction; (c) Appel reaction.

In addition to the exploration of the reaction scope, we sought to gain deeper insight into the mechanochemical Wittig reaction on sugars. Building on our previous findings with common aldehydes [38]—where the reaction was extremely rapid, completing within minutes—we aimed to determine whether the sugar substrates would exhibit a distinct kinetic behavior. With this aim, we performed a kinetic study of the mechanochemical Wittig reaction, using sugar **1a** as a model substrate (Figure 2A). It was found that the reaction proceeded swiftly within the first few minutes of milling, similar to conventional aldehydes. After only 5 min, a conversion of 49% to product **2a** was observed. This result is noteworthy, considering the predominance of the hemiacetal form in sugars and the limited availability of the reactive open‐chain aldehyde species. Following the rapid initial stage, the reaction rate progressively decreased, likely due to a reduction in the effective concentration of the reactants. Moreover, the accumulation of the liquid product **2a** and/or the additional formation of ^*t*^BuOH (from the ylide formation), which was shown to be detrimental (Table 1, entry 3), could also interfere with the progress of the reaction by forming a slurry‐like mixture, thereby slowing the mechanochemical process and requiring up to 1 h of milling to reach full conversion of the starting material.

To assess the synthetic practicality of the developed solvent‐free protocol, we performed the model Wittig reaction on a gram scale (Figure 2B). The reaction was conducted under standard milling conditions, with the only adjustment being the use of two grinding balls to ensure effective mixing. Pleasingly, when the reaction was performed on 1.0 g (3.8 mmol) of substrate **1a**, the process maintained its efficiency on scale‐up, affording compound **2a** in 0.81 g (83%) after isolation, thus confirming the practical utility of the developed mechanochemical protocol at larger scale.

To evaluate the efficiency and sustainability of the developed methodology, we calculated several green chemistry metrics. Specifically, our mechanochemical protocol was compared with a conventional solution‐based approach previously reported for the synthesis of **2a**, which was later applied in the total synthesis of the biologically active siladenoserinols A and D [51]. For consistency, the quantities of all materials were normalized to the reaction scale employed in our study (Figure 2C,D).

The mechanochemical approach offers operational simplifications, such as the generation of the ylide at room temperature in one minute, in contrast to a preformation step carried out at 0°C for 20 min in solution, significantly reducing the reaction time of this step compared to the solution‐based protocol. Moreover, the reaction can be performed under ambient conditions, without the need for an inert atmosphere. The protocol also operates under milder basic conditions, employing KO^*t*^Bu instead of LiHMDS.

From a sustainability perspective, environmental parameters such as the E‐factor and Process Mass Intensity (PMI) indicate the advantages of the mechanochemical protocol. Specifically, the E‐factor is approximately eight times lower (6.1 vs. 48.9), and the PMI is reduced by a factor of seven (7.1 vs. 49.9), reflecting the significant reduction in waste generation achieved by the mechanochemical protocol (Figure 2C,D).

The reported PMIs were calculated without consideration of solvents used in the work‐up procedure to allow direct comparison with the solution‐based approach. The PMI for the model reaction including the work‐up procedures is provided in Section D of the Supporting Information.

Finally, to demonstrate the synthetic utility of the developed protocol, we explored further functionalization of product **2j** via representative transformations previously employed in multistep synthesis (Figure 2E). The reaction conditions, which were not further optimized, are detailed in the Supporting Information (see Section C.9‐11 for procedures, and Section D.2‐4 for green chemistry metrics). First, the free hydroxyl group was protected as a tosylate under mechanochemical conditions, affording compound **3a** in 92% yield with a 15‐fold improved E‐factor compared to the solution‐phase protocol [52]. Next, we tested the Mitsunobu reaction, a transformation that has been demonstrated under mechanochemical conditions by Bolm and coworkers [53]. Gratifyingly, compound **3b** was obtained in excellent yield (86%), outperforming the reported solution‐based protocol (79%) in both efficiency and operational simplicity [54], and with an improved E‐factor (3.8 vs. 16.5). Finally, the Appel reaction was successfully applied to compound **2j** in the ball mill, delivering the synthetically valuable intermediate **3c** in 85% yield with an E‐factor reduced by a factor of 6 compared to the literature method in solution [52].

## 3 Conclusion

In conclusion, we have developed a solvent‐free mechanochemical protocol for the Wittig olefination of reducing sugar derivatives using ball milling. The method proved effective for three common protecting groups, is compatible with unprotected hydroxyl groups as well as unprotected sugars, and works across a broad range of monosaccharides—including hexoses, pentoses, and tetroses—demonstrating wide substrate generality and consistently high yields. The kinetic study confirmed the rapid initial conversion of the sugar under mechanochemical conditions, despite the limited availability of the open‐chain sugar form. By eliminating solvents, the reaction benefits from improved efficiency and operational simplicity, also at gram scale. Sustainability improvements were quantified through green chemistry metrics, with an E‐factor and Process Mass Intensity (PMI) approximately 8 and 7 times lower, respectively, compared to reported solution‐phase protocols. To further demonstrate the synthetic value of the method, selected downstream functionalizations were successfully performed under mechanochemical conditions, showcasing the applicability of this approach to multistep synthesis while drastically reducing the E‐factor. Overall, this work underscores how mechanochemistry can facilitate synthetically valuable carbohydrate functionalizations, providing access to versatile alkene motifs, while advancing greener synthetic approaches.

## Supporting Information

Additional supporting information can be found online in the Supporting Information section. **Supporting Fig. S1:** Setup for the mechanochemical reaction: (A) Retsch MM500 Vario vibratory ball mill. (B) PTFE jar of 8.2 mL and stainless‐steel milling ball (Ø: 12 mm, 7.5 g); a 0.1 € coin for comparison. **Supporting Fig. S2:** Starting materials investigated in this study. **Supporting Fig. S3:** Phosphonium salts used in this study as reagents for olefin diversification. **Supporting Fig. S4:** 400 MHz ^1^H NMR of **1e** as anomeric mixture *α*:*β* 0.7:1. **Supporting Fig. S5:** 101 MHz ^13^C NMR of **1e** as anomeric mixture *α*:*β* 0.7:1. **Supporting Fig. S6:** 600 MHz ^1^H NMR of **1f** as anomeric mixture *α*:*β* 2:1. **Supporting Fig. S7:** 151 MHz ^13^C NMR of **1f** as anomeric mixture *α*:*β* 2:1. **Supporting Fig. S8:** 600 MHz ^1^H NMR of **1g** as anomeric mixture *α*:*β* 1:0.8. **Supporting Fig. S9:** 151 MHz ^13^C NMR of **1g** as anomeric mixture *α*:*β* 1:0.8. **Supporting Fig. S10:** 400 MHz ^1^H NMR of **1i** as anomeric mixture *α*:*β* 0.1:1. **Supporting Fig. S11:** 101 MHz ^13^C NMR of **1i** as anomeric mixture *α*:*β* 0.1:1. **Supporting Fig. S12:** 600 MHz ^1^H NMR of **1j** as anomeric mixture *α*:*β* 0.15:1. **Supporting Fig. S13:** 151 MHz ^13^C NMR of **1j** as anomeric mixture *α*:*β* 0.15:1. **Supporting Fig. S14:** 400 MHz ^1^H NMR of **2a**. **Supporting Fig. S15:** 101 MHz ^13^C NMR of **2a**. **Supporting Fig. S16:** 400 MHz ^1^H NMR of **2b**. **Supporting Fig. S17:** 101 MHz ^13^C NMR of **2b**. **Supporting Fig. S18:** 400 MHz ^1^H NMR of **2c**. **Supporting Fig. S19:** 101 MHz ^13^C NMR of **2c**. **Supporting Fig. S20:** 400 MHz ^1^H NMR of **2d**. **Supporting Fig. S21:** 101 MHz ^13^C NMR of **2d**. **Supporting Fig. S22:** 400 MHz ^1^H NMR of **2e**. **Supporting Fig. S23:** 101 MHz ^13^C NMR of **2e**. **Supporting Fig. S24:** 400 MHz ^1^H NMR of **2f**. **Supporting Fig. S25:** 101 MHz ^13^C NMR of **2f**. **Supporting Fig. S26:** 400 MHz ^1^H NMR of **2g**. **Supporting** **Fig.** **27:** 101 MHz ^13^C NMR of **2g**. **Supporting Fig. S28:** 400 MHz ^1^H NMR of **2h**. **Supporting Fig. S29:** 101 MHz ^13^C NMR of **2h**. **Supporting Fig. S30:** 400 MHz ^1^H NMR of **2i**. **Supporting Fig. S31:** 101 MHz ^13^C NMR of **2i**. **Supporting Fig. S32:** 400 MHz ^1^H NMR of **2j**. **Supporting Fig. S33:** 101 MHz ^13^C NMR of **2j**. **Supporting Fig. S34:** 400 MHz ^1^H NMR of **2k** (*D*% = 75%). **Supporting Fig. S35:** 101 MHz ^13^C NMR of **2k** (*D*% = 75%). **Supporting Fig. S36:** 400 MHz ^1^H NMR of **2k** (*D*% = 84%). **Supporting Fig. S37:** 101 MHz ^13^C NMR of **2k** (*D*% = 84%). **Supporting Fig. S38:** 400 MHz ^1^H NMR of **2l**. **Supporting Fig. S39:** 151 MHz ^13^C NMR of **2l**. **Supporting Fig. S40:** 400 MHz ^1^H NMR of **2m**. **Supporting Fig. S41:** 101 MHz ^13^C NMR of **2m**. **Supporting Fig. S42:** 400 MHz ^1^H NMR of **2n**. **Supporting Fig. S43:** 101 MHz ^13^C NMR of **2n**. **Supporting Fig. S44:** 400 MHz ^1^H NMR of **2o**. **Supporting Fig. S45:** 101 MHz ^13^C NMR of **2o**. **Supporting Fig. S46:** 400 MHz ^1^H NMR of **2p**. **Supporting Fig. S47:** 101 MHz ^13^C NMR of **2p**. **Supporting Fig. S48:** 600 MHz ^1^H NMR of **2q**. **Supporting Fig. S49:** 151 MHz ^13^C NMR of **2q**. **Supporting Fig. S50:** 400 MHz ^1^H NMR of **2r**. **Supporting Fig. S51:** 101 MHz ^13^C NMR of **2r**. **Supporting Fig. S52:** 400 MHz ^1^H NMR of **2s**—E isomer. **Supporting Fig. S53:** 101 MHz ^13^C NMR of **2s**—E‐isomer. **Supporting Fig. S54:** 400 MHz ^1^H NMR of **2s**—Z isomer. **Supporting Fig. S55:** 101 MHz ^13^C NMR of **2s**—Z‐isomer. **Supporting Fig. S56:** 400 MHz ^1^H NMR of **2t**. **Supporting Fig. S57:** 101 MHz ^13^C NMR of **2t**. **Supporting Fig. S58:** 400 MHz ^1^H NMR of **2u**. **Supporting Fig. S59:** 101 MHz ^13^C NMR of **2u**. **Supporting Fig. S60:** 600 MHz ^1^H NMR of **2v**. **Supporting Fig. S61:** 151 MHz ^13^C NMR of **2v**. **Supporting Fig. S62:** 400 MHz ^1^H NMR of **3a**. **Supporting Fig. S63:** 101 MHz ^13^C NMR of **3a**. **Supporting Fig. S64:** 400 MHz ^1^H NMR of **3b**. **Supporting Fig. S65:** 101 MHz ^13^C NMR of **3b**. **Supporting Fig. S66:** 400 MHz ^1^H NMR of **3c**. **Supporting Fig. S67:** 101 MHz ^13^C NMR of **3c**. **Supporting Fig. S68:** 400 MHz ^1^H NMR of **2b′**. **Supporting Fig. S69:** 101 MHz ^13^C NMR of **2b′**. **Supporting Fig. S70:** 400 MHz ^1^H NMR of **2f′**. **Supporting Fig. S71:** 101 MHz ^13^C NMR of **2f′**. **Supporting Table S1:** Screening of Liquid‐Assisted Grinding (LAG) agents. ^a^Yields determined by ^1^H‐NMR spectroscopy, using 3,4,5‐trichloropyridine as internal standard. **Supporting Table S2:** Screening of the amount of phosphonium bromide and the base. ^a^Yields determined by ^1^H‐NMR spectroscopy, using 3,4,5‐trichloropyridine as internal standard. **Supporting Table S3:** Screening of the base. ^a^Yields determined by ^1^H‐NMR spectroscopy, using 3,4,5‐trichloropyridine as internal standard. **Supporting Table S4:** Screening of the phosphonium counterion. ^a^Yields determined by ^1^H‐NMR spectroscopy, using 3,4,5 trichloropyridine as internal standard. **Supporting Table S5:** Screening of milling frequencies and grinding media (grinding ball purchased commercially, Ø: 12 mm). ^a^Yields determined by ^1^H‐NMR spectroscopy, using 3,4,5‐trichloropyridine as internal standard. **Supporting Table S6:** Screening of milling time. ^a^Yields determined by ^1^H‐NMR spectroscopy, using 3,4,5‐trichloropyridine as internal standard. **Supporting Table S7:** Screening of best conditions for benzyl‐protected D‐mannose. ^a^Yields determined by ^1^H‐NMR spectroscopy, using 3,4,5‐trichloropyridine as internal standard. **Supporting Table S8:** Screening of best conditions for benzyl‐protected d‐glucose. ^a^Yields determined by ^1^H‐NMR spectroscopy, using 3,4,5‐trichloropyridine as internal standard. **Supporting Table S9:** Screening of best conditions for benzyl‐protected D‐galactose. ^a^Yields determined by ^1^H‐NMR spectroscopy, using 3,4,5‐trichloropyridine as internal standard. **Supporting Table S10:** Screening of best conditions for benzyl‐protected d‐xylose. ^a^Yields determined by ^1^H‐NMR spectroscopy, using 3,4,5‐trichloropyridine as internal standard. **Supporting Table S11:** Screening of best conditions for benzyl‐protected d‐arabinose. ^a^Yields determined by ^1^H‐NMR spectroscopy, using 3,4,5‐trichloropyridine as internal standard. **Supporting Table S12:** Green metrics calculation for the model reaction (on left), and comparison with the solution approach (on right).^7^ **Supporting Table S13:** Green metrics calculation for the synthesis of **3a** (on left) and comparison with the solution approach (on right).^8^ **Supporting Table S14:** Green metrics calculation for the synthesis of **3b** (on left) and comparison with the solution approach (on right).^9^ **Supporting Table S15:** Green metrics calculation for the synthesis of **3c** (on left) and comparison with the solution approach (on right).^8^ **Supporting Table S16:** Comparison between mechanochemical and solution‐phase conditions for the Wittig olefination of sugars.

## Funding

This research was funded in whole or in part by the Austrian Science Fund (FWF) [10.55776/P33064]. For open access purposes, the author has applied a CC BY public copyright license to any author accepted manuscript version arising from this submission.

## Conflicts of Interest

The authors declare no conflicts of interest.

## Supporting information

Supplementary Material
