# Supplementary material for: Mechanochemistry Enables Rapid and Solvent‐Free Wittig Reactions on Sugars
Source: ChemSusChem. 2025 Dec 16;19(1):e202502026. doi: 10.1002/cssc.202502026 (PMC12767755; doi:10.1002/cssc.202502026)
Supplement: Supplementary file 1 — Supplementary Material [file CSSC-19-e202502026-s001.pdf]

SUPPORTING INFORMATION

Mechanochemistry Enables Rapid and Solvent-Free Wittig Reactions on Carbohydrates

FRANCESCO MELE, NINA BIEDERMANN, CHRISTOPH SUSTER, JOHANNA TEMPL, CHRISTIAN STANETTY*, MICHAEL SCHNÜRCH*

GENERAL INFORMATION **2**

A. OPTIMIZATION OF REACTION CONDITIONS **3**

A.1. REACTION OPTIMIZATION OF ACETONIDE-PROTECTED D-MANNOSE DERIVATIVE 3
A.2. SELECTION OF BASE FOR BENZYL-PROTECTED SUGARS 7

B. OVERVIEW OF STARTING MATERIALS **10**

B.1. CARBOHYDRATES 10
B.2. PHOSPHONIUM SALTS 10

C. EXPERIMENTAL PROCEDURES **11**

C.1. GENERAL PROCEDURE A1 – BENZYL PROTECTION (1E-1G) 11
C.2. GENERAL PROCEDURE A2 – SYNTHESIS OF 2,3-*O*-ISOPROPYLIDENE-D-*RIBO*-FURANOSE (1I) 11
C.3. GENERAL PROCEDURE A3 – SYNTHESIS OF 2,3-*O*-ISOPROPYLIDENE-L-*ERYTHRO*-FURANOSE (1J) 12
C.4. DEUTERATION OF THE METHYLTRIPHENYLPHOSPHONIUM BROMIDE 12
C.5. SYNTHESIS OF THE WITTIG SALT V 13
C.6. GENERAL PROCEDURE B1 – MECHANOCHEMICAL WITTIG REACTION OF ACETAL-PROTECTED SUGARS 13
C.7. GENERAL PROCEDURE B2 – MECHANOCHEMICAL WITTIG REACTION OF BENZYL-PROTECTED SUGARS 14
C.8. MECHANOCHEMICAL WITTIG REACTION OF 1A ON GRAM SCALE 14
C.9. MECHANOCHEMICAL TOSYLATION OF 2J 15
C.10. MECHANOCHEMICAL MITSUNOBU REACTION OF 2J 15
C.11. MECHANOCHEMICAL APPEL REACTION OF 2J 16

D. GREEN METRICS CALCULATION **17**

D.1. GREEN METRICS CALCULATION FOR MODEL REACTION 17
D.2. GREEN METRICS CALCULATION FOR 3A 17
D.3. GREEN METRICS CALCULATION FOR 3B 18
D.4. GREEN METRICS CALCULATION FOR 3C 18

E. GENERAL COMPARISON WITH SOLUTION APPROACH **19**

F. CHARACTERISATION DATA **20**

F.1. STARTING MATERIALS 20
F.2. OLEFINATION PRODUCTS 22
F.3. UTILIZATION PRODUCTS 30
F.4. DIENE-PRODUCTS 31

G. REFERENCES **32**

H. NMR SPECTRA **33**

H.1. STARTING MATERIALS 33
H.2. OLEFINATION PRODUCTS 38
H.3. UTILIZATION PRODUCTS 62
H.4. DIENE-PRODUCTS 65

General Information

Reagents and Consumables

Unless noted otherwise, all chemicals were purchased from commercial sources and used without further purification. Column chromatography was performed on standard manual glass columns using Merck silica gel 60 (40 – 63 µm). Thin Layer Chromatography (TLC) was performed on aluminium-backed unmodified Merck silica gel 60 F_{254} plates. Spots were visualized via UV irradiation at 254 nm or anis aldehyde staining solution (180 mL EtOH, 10 mL *p*-anisaldehyde, 10 mL H_2SO_4 conc., 2 mL acetic acid).

NMR Spectroscopy

All NMR spectra were recorded on either a Bruker Avance UltraShield 400 NMR ("400 MHz machine") at ambient temperature or on a Bruker Avance III HD 600 spectrometer equipped with a prodigy N_2-cryogenic probe head. Spectra were referenced to the residual solvent signal. Coupling constants (*J*) are given in Hz and multiplicities are assigned as s (singlet), d (doublet), t (triplet), q (quartet), m (multiplet) or combinations thereof. Product mixtures of *E-/Z*-isomers were determined from ^{1}H-NMR spectra *via* comparison of the coupling constants of defined multiplet signals and with literature spectra. The assignment of *E-/Z*-isomers to ^{13}C-NMR spectra signals was conducted *via* 2D NMR experiments (HSQC and HMBC) and by comparison with literature spectra.

Mechanochemistry Equipment

Mechanochemical reactions were conducted in a Retsch MM500 vario horizontal movement Shaker Mill. This mill has an adjustable frequency from 3 Hz to 35 Hz with an adjustable timer. Unless otherwise indicated, the reactions were conducted in a PTFE milling jar (8.2 mL), charged with one hardened steel milling ball (Ø: 12 mm, 7.5 g).

High Resolution Mass Spectrometry (HRMS)

HRMS analysis was performed using HTC PAL system auto sampler, an Agilent 1100/1200 HPLC and Agilent 6230 AJS ESI-TOF mass spectrometer. Samples were dissolved in methanol, acetonitrile or mixtures thereof. Data evaluation was performed using Agilent MassHunter Qualitative Analysis B.07.00. Identification was based on peaks obtained from extracted ion chromatograms (extraction width ± 20 ppm).

Melting points:

Melting points were measured on a Büchi Melting Point B-545 system, with 40%/90% threshold detection and a heating rate of 0.5 °C/min.

A. Optimization of Reaction Conditions

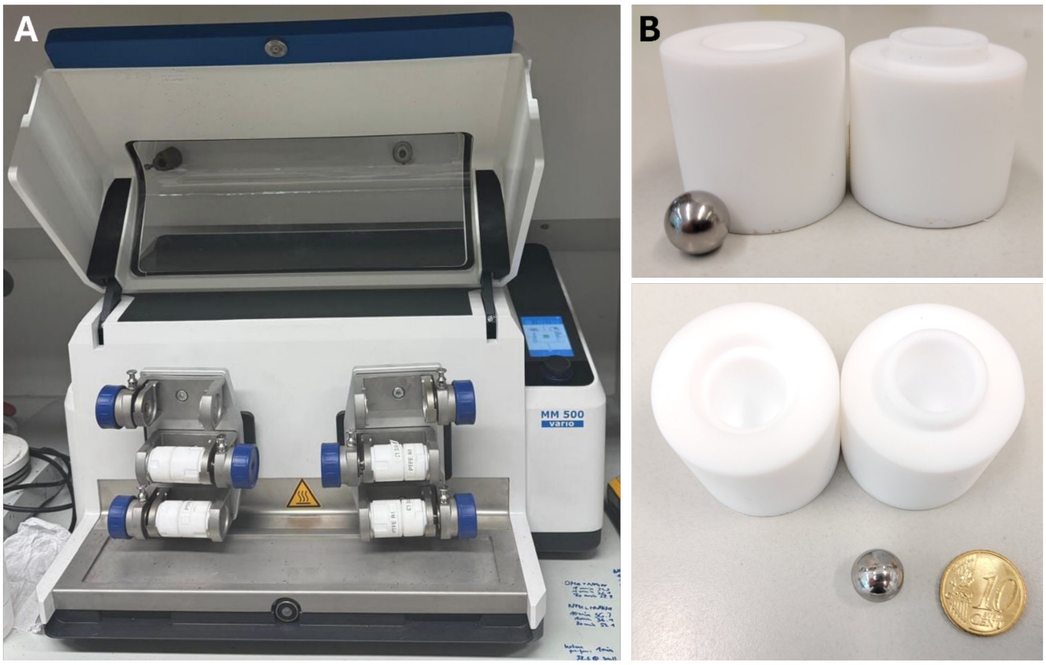

Figure S1. Setup for the mechanochemical reaction: A) Retsch MM500 Vario vibratory ball mill. B) PTFE jar of 8.2 mL and stainless-steel milling ball (Ø: 12 mm, 7.5 g); a 0.1 € coin for comparison.

A.1. Reaction Optimization of Acetonide-Protected D-Mannose Derivative

The reaction conditions were optimized on the reaction of D-*mannose* configured substrate **1a** and methyltriphenylphosphonium bromide to give corresponding olefin **2a** on a 0.2 mmol scale (See Table S1 to Table S6)

A 8.2 mL PTFE milling jar equipped with one 12 mm hardened steel ball (unless stated otherwise) was charged with the selected methyltriphenylphosphonium halide (*x* equiv.) and base (*x* equiv.). The vessel was mounted onto the holding station of a Retsch MM500 mixer mill, and milling was performed at a frequency of *x* Hz for 1 minute. Subsequently, the jar was opened, and the sugar **1a** (52.1 mg, 0.20 mmol, 1.0 equiv.) and the LAG agent (if necessary) were added. The jar was then resealed, and milling was continued at *x* Hz for an additional 1 hour. Upon completion, the crude reaction mixture was recovered using dichloromethane (DCM, 10 mL), quenched with saturated aqueous NH_4Cl (10 mL), and extracted with DCM (3 × 10 mL). The combined organic layers were dried over Na_2SO_4, filtered, concentrated under reduced pressure, and the sample was analyzed via ^1H NMR spectroscopy ($CDCl_3$, 3,4,5-trichloropyridine as internal standard).

A.1.1. Liquid Assisted Grinding (LAG) screening

Table S1. *Screening of Liquid-Assisted Grinding (LAG) agents. [a]Yields determined by ^{1}H-NMR spectroscopy, using 3,4,5-trichloropyridine as internal standard.*

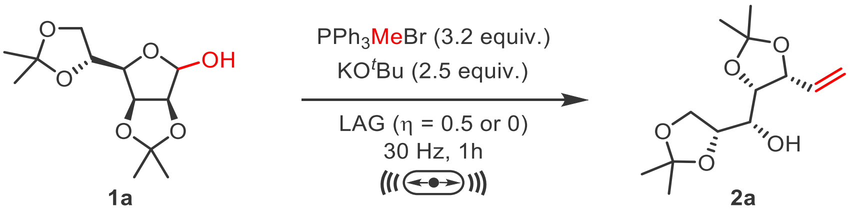

Entry	LAG	Yield (%)[a]
1	Toluene	85
2	^{t}BuOH	52
3	THF	91
**4**	**Neat**	**92**

A.1.2. Reagent amount

Table S2. *Screening of the amount of phosphonium bromide and the base. [a]Yields determined by ^{1}H-NMR spectroscopy, using 3,4,5-trichloropyridine as internal standard.*

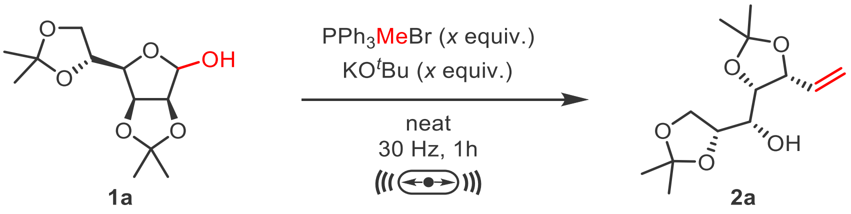

Entry	PPh_3MeBr	KO^{t}Bu	Yield (%)[a]
1	5 equiv.	4 equiv.	94
**2**	**3.2 equiv.**	**2.5 equiv.**	**92**
3	1.5 equiv.	1.2 equiv.	46

A.1.3. Base screening

Table S3. Screening of the base. [a]Yields determined by ^{1}H-NMR spectroscopy, using 3,4,5-trichloropyridine as internal standard.

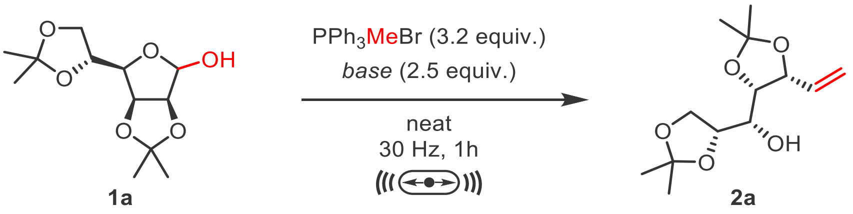

Entry	base	Yield (%)[a]
**1**	**KO^{t}Bu**	**92**
2	Cs_2CO_3	66
3	NaOH	-
4	DBU	-
5	LiHMDS	46
6	NaHMDS	73

A.1.4. Screening of phosphonium salts

Table S4. Screening of the phosphonium counterion. [a]Yields determined by ^{1}H-NMR spectroscopy, using 3,4,5-trichloropyridine as internal standard.

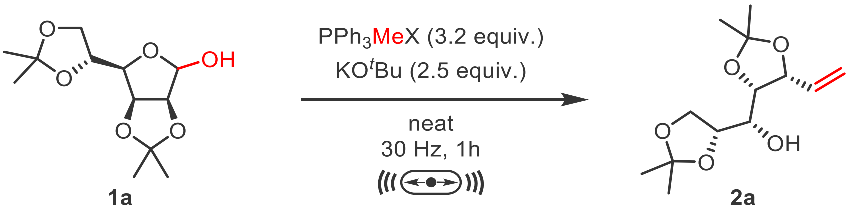

Entry	X	Yield (%)[a]
1	Cl	8
**2**	**Br**	**92**
3	I	21

A.1.5. Screening of milling parameters

Table S5. *Screening of milling frequencies and grinding media (grinding ball purchased commercially, Ø: 12 mm).* [a]*Yields determined by* ^{1}*H-NMR spectroscopy, using 3,4,5-trichloropyridine as internal standard.*

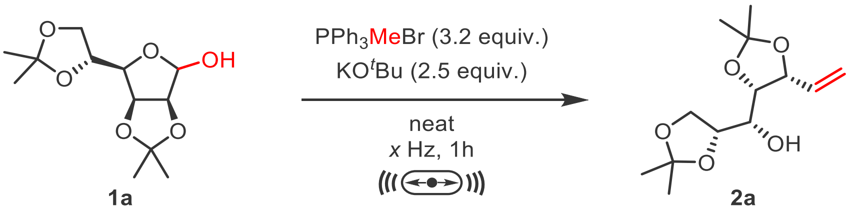

Entry	Frequency (Hz)	Grinding Media	Yield (%)[a]
1	20	stainless-steel	51
2	30	stainless-steel	92
**3**	**35**	**stainless-steel**	**96**
4	30	ZrO_2	87

A.1.6. Screening of milling time

Table S6. *Screening of milling time.* [a]*Yields determined by* ^{1}*H-NMR spectroscopy, using 3,4,5-trichloropyridine as internal standard.*

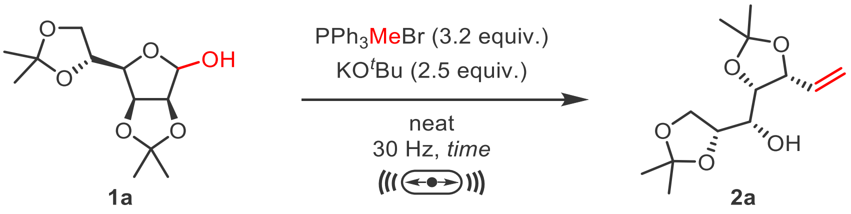

Entry	time (min)	Yield (%)[a]
1	1	28
2	5	49
3	15	72
4	30	87
5	45	89
**6**	**60**	**92**

A.2. Selection of Base for Benzyl-Protected Sugars

After investigating the reactivity of acetonide-protected D-mannose, we turned to benzyl-protected sugars. In this case, KO^{t}Bu was no longer the optimal base in terms of yield and selectivity, prompting us to test some HMDS bases to identify the conditions that afforded the best reaction yields.

A.2.1. Screening of bases for D-mannose

The benzyl-protected D-mannose **1b** showed a divergent reactivity when KO^{t}Bu was used as the base. Instead of the expected Wittig product, the reaction led selectively to the formation of the conjugated diene **2b'** via elimination of benzylic alcohol (Table S8, entry 1). This outcome is consistent with literature reports by Luo et al.,[1] describing similar reactivity patterns for benzyl-protected sugars in solution. To address this issue, we followed their strategy and employed HMDS-type bases to suppress the undesired elimination. Under our solvent-free conditions, these bases proved effective, with LiHMDS providing the best results in terms of both yield and selectivity for product **2b** (Table S8, entry 3).

Table S7. *Screening of best conditions for benzyl-protected D-mannose. [a]Yields determined by ^{1}H-NMR spectroscopy, using 3,4,5-trichloropyridine as internal standard.*

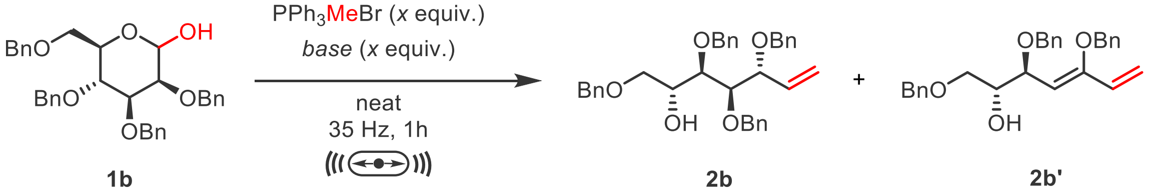

Entry	PPh₃MeBr	base	Yield (%) of 2b[a]	Yield (%) of 2b'[a]
1	3.2 equiv.	KO^{t}Bu (2.5 equiv.)	-	73
2	3.2 equiv.	NaHMDS (2.5 equiv.)	26	52
**3**	**3.2 equiv.**	**LiHMDS (2.5 equiv.)**	**82**	**-**

A.2.2. Screening of bases for D-glucose

Table S8. *Screening of best conditions for benzyl-protected D-glucose. [a]Yields determined by ^{1}H-NMR spectroscopy, using 3,4,5-trichloropyridine as internal standard.*

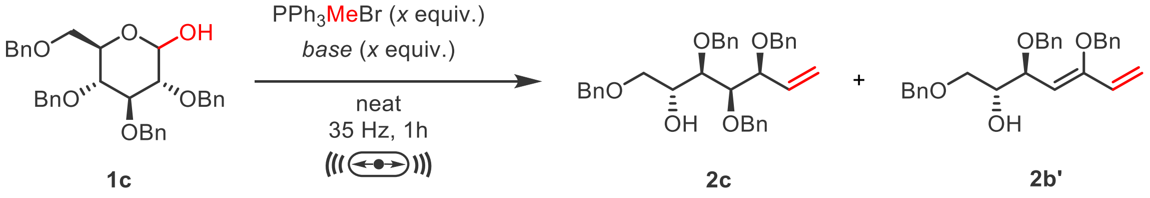

Entry	PPh₃MeBr	base	Yield (%) of 2c[a]	Yield (%) of 2b'[a]
1	3.2 equiv.	KO^{t}Bu (2.5 equiv.)	-	91
**2**	**3.2 equiv.**	**NaHMDS (2.5 equiv.)**	**90**	**-**
3	3.2 equiv.	LiHMDS (2.5 equiv.)	82	-

A.2.3. Screening of bases for D-galactose

Table S9. *Screening of best conditions for benzyl-protected* D*-galactose.* [a]*Yields determined by* [1]*H-NMR spectroscopy, using 3,4,5-trichloropyridine as internal standard.*

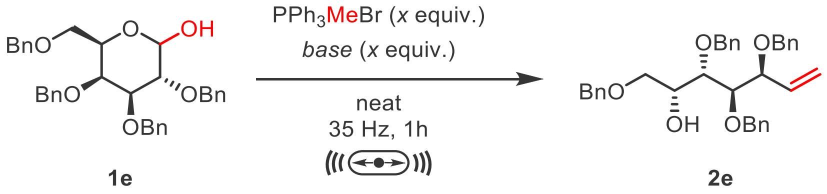

Entry	PPh_3MeBr	base	Yield (%)[a]
1	3.2 equiv.	LiHMDS (2.5 equiv.)	10
**2**	**3.2 equiv.**	**NaHMDS (2.5 equiv.)**	**81**

A.2.4. Screening of conditions for D-xylose

Table S10. *Screening of best conditions for benzyl-protected D-xylose. [a]Yields determined by ^{1}H-NMR spectroscopy, using 3,4,5-trichloropyridine as internal standard.*

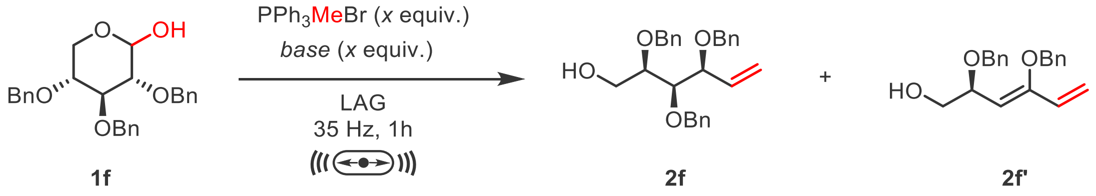

Entry	P salt	Base	LAG (η = 0.5)	time	Yield (%) of 2f[a]	Yield (%) of 2f'[a]
1	1.4 equiv.	KO^tBu (1.2 equiv.)	-	1h	.	84
2	5 equiv.	NaHMDS (4 equiv.)	-	30 min	42	9
3	5 equiv.	NaHMDS (4 equiv.)	Toluene	30 min	38	-
4	5 equiv.	NaHMDS (4 equiv.)	THF	30 min	37	11
5	5 equiv.	LiHMDS (4 equiv.)	-	30 min	62	-
**6**	**3.2 equiv.**	**LiHMDS (2.5 equiv.)**	**-**	**1 h**	**64**	-
7	3 equiv.	LiHMDS (3.5 equiv.)	-	1 h	61	-

A.2.5. Screening of bases for D-arabinose

Table S11. *Screening of best conditions for benzyl-protected D-arabinose. [a]Yields determined by ^{1}H-NMR spectroscopy, using 3,4,5-trichloropyridine as internal standard.*

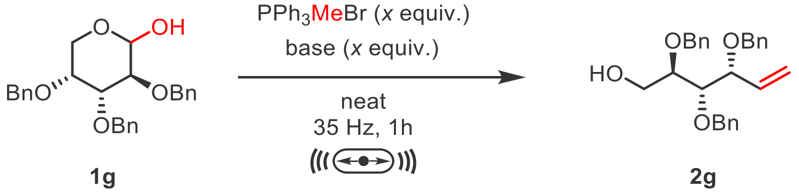

Entry	PPh_3MeBr	base	Yield (%)[a]
1	3.25 equiv.	LiHMDS (2.5 equiv.)	32
**2**	**3.2 equiv.**	**NaHMDS (2.5 equiv.)**	**57**

B. Overview of starting materials

B.1. Carbohydrates

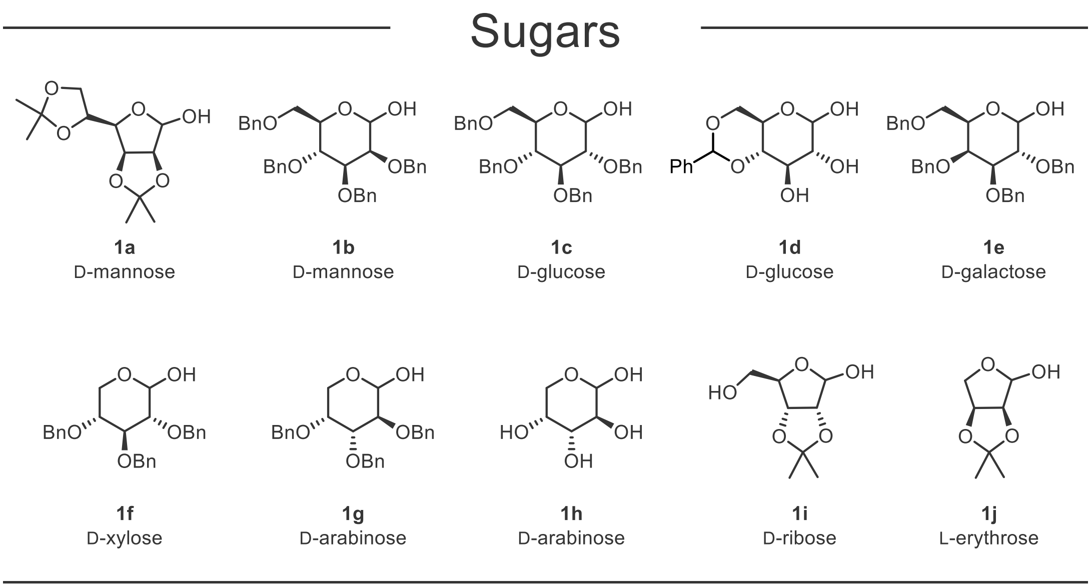

Figure S2. *Starting materials investigated in this study.*

B.2. Phosphonium salts

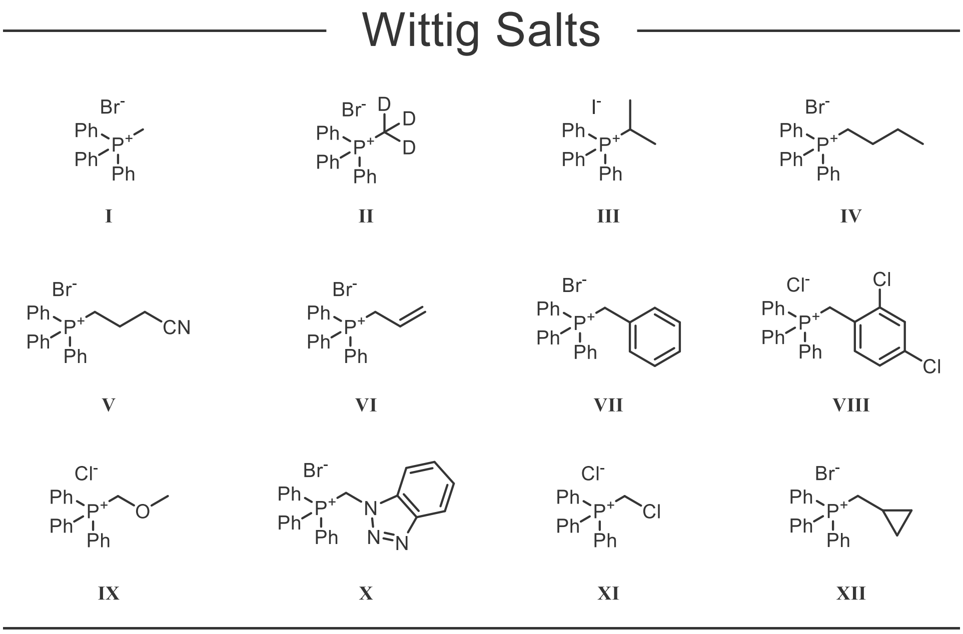

Figure S3. *Phosphonium salts used in this study as reagents for olefin diversification.*

C. Experimental procedures

C.1. General procedure A1 – Benzyl Protection (1e-1g)

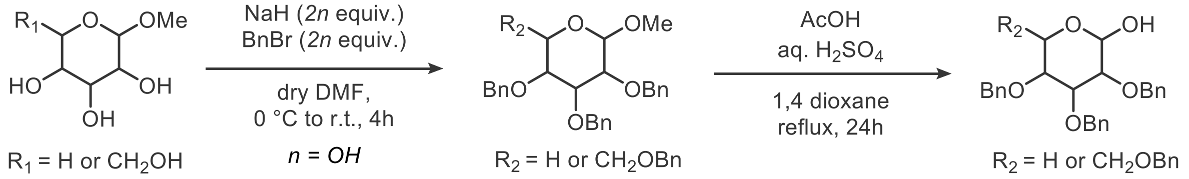

Step 1 (Benzyl protection): The desired methyl-β-D-pyranoside (3 mmol, 1.0 equiv.) was transferred into a 100 mL three-neck-flask equipped with a dropping funnel and a magnetic stirrer. The flask was set under argon atmosphere and 35 mL dry DMF were added via transfer canula. The solution was cooled to 0 °C with an ice-bath. Upon reaching the desired temperature, NaH as 60% dispersion in paraffin oil (2.0 equiv. per OH group) was added under vigorous stirring, in three equal portions over 5 minutes. The solution was stirred for 30 min at 0 °C. Then, benzyl bromide (2.0 equiv. per OH group) was added dropwise over 10 minutes. Upon complete addition, the ice bath was removed, and the mixture was allowed to warm to room temperature. After 3 h, the reaction was cooled to 0 °C, before 10 mL of MeOH were added slowly to quench the excess of NaH. The mixture was stirred for further 10 min, before 200 mL EtOAc were added, and phases were separated, and the organic layer was washed 5 times with 200 mL H_2O each. The combined organic layers were dried over Na_2SO_4, filtered and evaporated (twice co-evaporated from toluene) to give a colourless residue. The residue was purified by flash chromatography (hexane/EtOAc 8:1) to give the corresponding methyl *O*-benzyl-β-D-pyranoside that was directly used in the next step.

Step 2 (Hydrolysis): The desired methyl *O*-benzyl-β-D-pyranoside (3 mmol, 1.0 equiv.) was suspended in 45 mL of a 1:1:1 mixture of acetic acid, dioxane and 1M H_2SO_4. The mixture was refluxed for 24h. The reaction was allowed to cool to room temperature, and the crude mixture was extracted with EtOAc (3 × 100 mL). Then, the combined organic layers were neutralised with sat. $NaHCO_3$ solution, dried over Na_2SO_4, filtered and evaporated, to give crude material as a colourless oil. The desired target material was obtained *via* crystallization from cold hexane or by flash chromatography (hexane/EtOAc in different ratio).

*Reference: Adapted from the literature procedure described by I. S. Kim et al., Org. Lett., 2006, **8**, 4101–4104.*[2]

C.2. General Procedure A2 – Synthesis of 2,3-*O*-isopropylidene-D-*ribo*-furanose (1i)

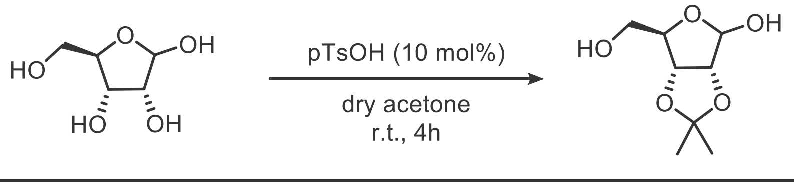

p-Toluenesulfonic acid (172 mg, 1 mmol, 0.1 equiv.) was added to a suspension of D-ribose (1.5 g, 10 mmol, 1.0 equiv.) in dry acetone. After stirring for 4 h, Et_3N (0.45 mL) was added, and the resulting mixture was stirred for an additional 10 minutes. The residue was concentrated and purified by flash chromatography (hexane/EtOAc 2:1) to afford the title compound **1i** as a colourless oil (1.48 g, 78%).

*Reference: N. A. Ivanova et al., Russ J Org Chem, 2008, **44**, 335–339.*[3]

C.3. General Procedure A3 – Synthesis of 2,3-*O*-isopropylidene-L-*erythro*-furanose (1j)

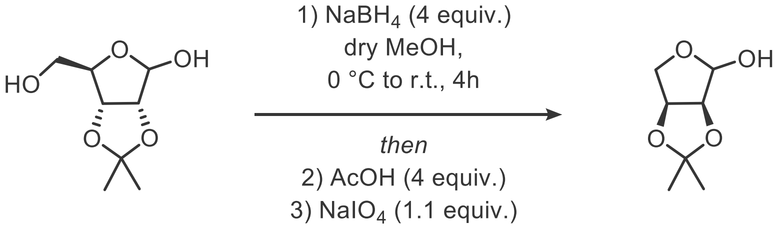

$NaBH_4$ (454 mg, 12 mmol, 4.0 equiv.) was added cautiously to a solution of 2,3-*O*-Isopropylidene-D-ribofuranose (**1i**) (570 mg, 3 mmol, 1.0 equiv.) in dry methanol (6 mL) at 0 °C. After stirring for 4 h at ambient temperature, acetic acid (0.7 mL, 12 mmol, 4.0 equiv.) was added dropwise, and the mixture was stirred for 10 minutes until the excess borohydride had decomposed. Sodium periodate (706 mg, 3.3 mmol, 1.1 equiv.) was added in portions over 10 min, and the reaction was then stirred for 1 h at ambient temperature. The resulting mixture was filtered through Celite®, which was rinsed with DCM (50 mL). The mixture was washed with brine and extracted with DCM (3 × 25 mL), dried over Na_2SO_4, filtered and concentrated to give a colourless residue. The residue was purified by flash chromatography (hexane/EtOAc 2:1) to give the title compound **1j** as a colourless oil (184 mg, 38%).

*Reference: M. Heinrich et al., J. Am. Chem. Soc., 2020, **142**, 6409–6422.*[4]

C.4. Deuteration of the Methyltriphenylphosphonium Bromide

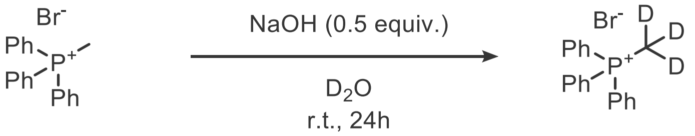

I II

To a flask with a magnetic stir bar was added Ph_3PMeBr (530mg, 1.5 mmol, 1.0 equiv), D_2O (1.5 mL), and NaOH (30 mg, 0.75 mmol, 0.5 equiv). The reaction mixture was stirred under nitrogen atmosphere, and after 24 h, DCM (5 mL) was added to the solution and organic layer was collected, dried with Na_2SO_4, and concentrated to give **II** as a colourless solid (436 mg, 81% yield).

*Reference: Z. Zhao et al., Angew. Chem. Int. Ed. 2017, **56**, 11620.*[5]

C.5. Synthesis of the Wittig salt V

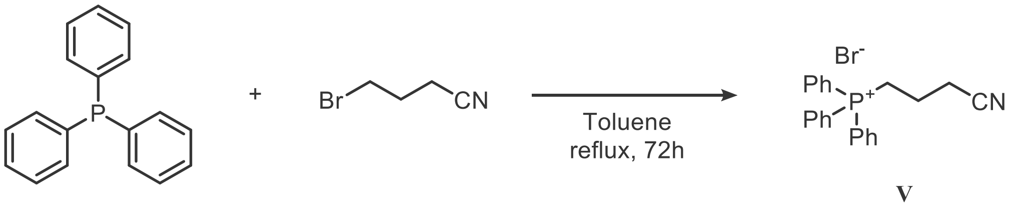

A solution of triphenylphosphine (1.0 equiv., 2 mmol, 525 mg) and 4-bromobutyronitrile (1.0 equiv., 2 mmol, 296 mg, 0.20 mL) in anhydrous toluene (10 mL) was refluxed under argon for 72 hours, observing the formation of a white precipitate. After the reaction mixture was cooled, the product was collected by filtration, washed with cold toluene, and dried under vacuum. The phosphonium salt **V** was obtained as a colourless solid (612 mg, 75% yield).

*Reference: P. Bonilla et al., Angew. Chem. Int. Ed. 2018, **57**, 12819.*[6]

C.6. General Procedure B1 – Mechanochemical Wittig Reaction of Acetal-Protected Sugars

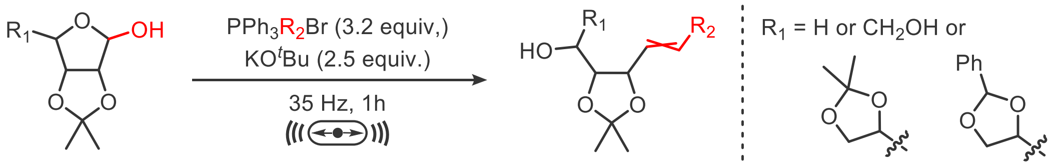

A 8.2 mL PTFE milling jar equipped with one 12 mm hardened steel ball was charged with the respective phosphonium salt (0.64 mmol, 3.2 equiv.) and KO^{t}Bu (56.1 mg, 0.5 mmol, 2.5 equiv.). The vessel was mounted onto the holding station of a Retsch MM500 mixer mill, and milling was performed at a frequency of 35 Hz for 1 minute. Subsequently, the jar was opened, and the sugar substrate (0.20 mmol, 1.0 equiv.) was added. The jar was then resealed, and milling was continued at 35 Hz for an additional 1 hour (unless stated otherwise). Upon completion, the crude reaction mixture was recovered using dichloromethane (DCM, 10 mL), quenched with saturated aqueous NH_4Cl (10 mL), and extracted with DCM (3 × 10 mL). The combined organic layers were dried over Na_2SO_4, filtered, and concentrated under reduced pressure. The residue was purified by flash chromatography (hexane/EtOAc in different ratio) to yield the target compound.

C.7. General Procedure B2 – Mechanochemical Wittig Reaction of Benzyl-Protected Sugars

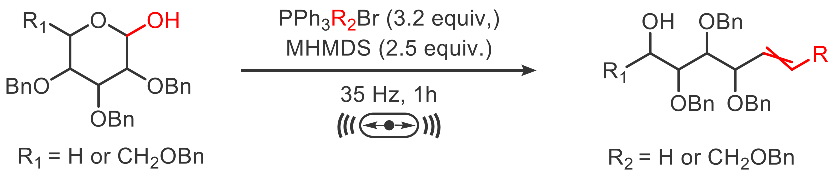

A 8.2 mL PTFE milling jar equipped with one 12 mm hardened steel ball was charged with the respective phosphonium salt (0.64 mmol, 3.2 equiv.) and the HMDS base (NaHMDS (91.7 mg, 0.5 mmol, 2.5 equiv.) or LiHMDS (83.7 mg, 0.5 mmol, 2.5 equiv.)). The vessel was mounted onto the holding station of a Retsch MM500 mixer mill, and milling was performed at a frequency of 35 Hz for 1 minute. Subsequently, the jar was opened and the sugar substrate (0.20 mmol, 1.0 equiv.) was added. The jar was then resealed and milling was continued at 35 Hz for an additional 1 hour (unless stated otherwise). Upon completion, the crude reaction mixture was recovered using dichloromethane (DCM, 10 mL), quenched with saturated aqueous NH_4Cl (10 mL), and extracted with DCM (3 × 10 mL). The combined organic layers were dried over Na_2SO_4, filtered, and concentrated under reduced pressure. The residue was purified by flash chromatography (hexane/EtOAc in different ratio) to yield the target compound.

C.8. Mechanochemical Wittig Reaction of 1a on Gram Scale

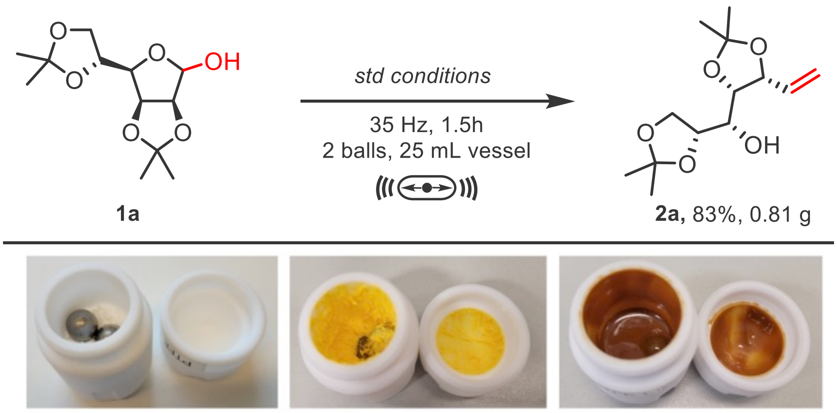

Before milling *Ylide pre-formation* *After milling*

A 25 mL PTFE milling jar equipped with two 12 mm hardened steel balls was charged with methyltriphenylphosphonium bromide (4.2 g, 11.4 mmol, 3 equiv.) and KO^{t}Bu (1.1 g, 9.5 mmol, 2.5 equiv.). The vessel was mounted onto the holding station of a Retsch MM500 mixer mill, and milling was performed at a frequency of 35 Hz for 5 minutes. Subsequently, the jar was opened, and **1a** (1.0 g, 3.8 mmol, 1.0 equiv.) was added. The jar was then resealed, and milling was continued at 35 Hz for 1.5 hours. Upon completion, the crude reaction mixture was recovered using dichloromethane (DCM, 30 mL), quenched with saturated aqueous NH_4Cl (30 mL), and extracted with DCM (3 × 20 mL). The combined organic layers were dried over Na_2SO_4, filtered, and concentrated under reduced pressure. The residue was purified by flash chromatography (hexane/EtOAc 10:1) to yield **2a** (815 mg, 83% yield).

C.9. Mechanochemical Tosylation of 2j

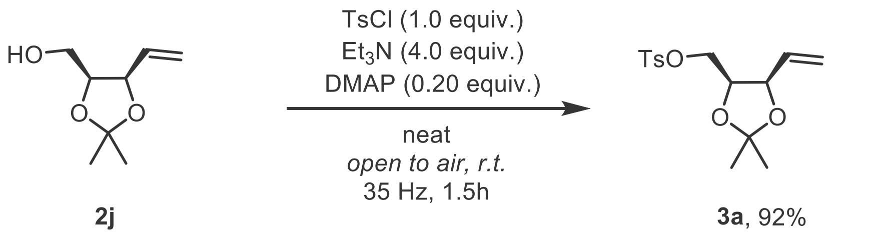

A 8.2 mL PTFE milling jar equipped with one 12 mm hardened steel ball was charged with tosyl chloride (38.5 mg, 0.2 mmol, 1.0 equiv.), DMAP (5.0 mg, 0.04 mmol, 0.20 equiv.), Et_3N (112 µL, 0.8 mmol, 4.0 equiv.), and **2j** (31.6 mg, 0.2 mmol, 1.0 equiv.). The vessel was mounted onto the holding station of a Retsch MM500 mixer mill, and milling was performed at a frequency of 35 Hz for 1.5 h. Upon completion, the crude reaction mixture was recovered using dichloromethane (DCM, 10 mL), washed with water and extracted with DCM (3 × 10 mL). The organic phases were sequentially washed with aq. HCl 1.0 M (10 mL) and sat. aq. $NaHCO_3$ (10 mL). The combined organic layers were dried over Na_2SO_4, filtered, and concentrated under reduced pressure. The residue was purified by flash chromatography (hexane/EtOAc 9:1) to yield **3a** (57.5 mg, 92% yield).

C.10. Mechanochemical Mitsunobu Reaction of 2j

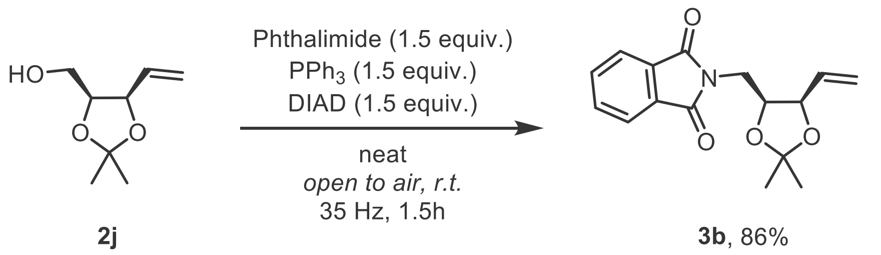

A 8.2 mL PTFE milling jar equipped with one 12 mm hardened steel ball was charged with phthalimide (45.0 mg, 0.3 mmol, 1.5 equiv.), PPh_3 (78.7 mg, 0.3 mmol, 1.5 equiv.), **2j** (31.6 mg, 0.2 mmol, 1.0 equiv.), and DIAD (63 µL, 0.3 mmol, 1.5 equiv.). The vessel was mounted onto the holding station of a Retsch MM500 mixer mill, and milling was performed at a frequency of 35 Hz for 1.5 h. Upon completion, the crude reaction mixture was recovered using dichloromethane and directly purified by flash chromatography (hexane/EtOAc 12:1) to yield **3b** (49.4 mg, 86% yield).

C.11. Mechanochemical Appel Reaction of 2j

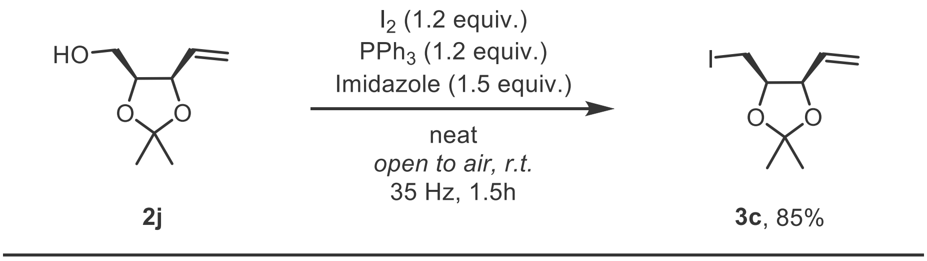

A 8.2 mL PTFE milling jar equipped with one 12 mm hardened steel ball was charged with imidazole (20.6 mg, 0.3 mmol, 1.5 equiv.), PPh_3 (62.9 mg, 0.24 mmol, 1.2 equiv.), **2j** (31.6 mg, 0.2 mmol, 1.0 equiv.), and iodine (60.9 mg, 0.24 mmol, 1.2 equiv.). The vessel was mounted onto the holding station of a Retsch MM500 mixer mill, and milling was performed at a frequency of 35 Hz for 1.5 h. Upon completion, the crude reaction mixture was recovered using ethyl acetate (15 mL), and was sequentially washed with 10% aq. $Na_2S_2O_3$ (10 mL) and water (10 mL). The organic layer was dried over Na_2SO_4, filtered, and concentrated under reduced pressure. The residue was purified by flash chromatography (hexane/EtOAc 80:1) to yield **3c** (45.6 mg, 85% yield).

D. Green Metrics Calculation

D.1. Green Metrics Calculation for Model Reaction

***Table S12**. Green metrics calculation for the model reaction (on left), and comparison with the solution approach (on right).*[7]

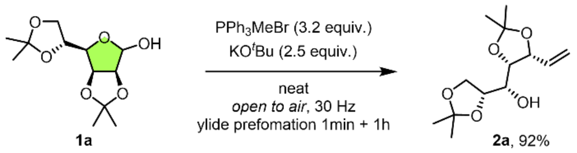

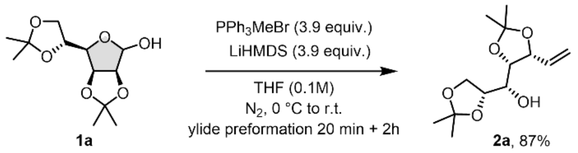

Substance	MW	n (mmol)	weight (mg)
Substrate **1a**	260.1	0.2	52.0
PPh_3MeBr	356	0.64	227.8
KO^tBu	112	0.5	56.0
**Solvent**	**0**	**0**	**0**
Product **2a**	258.1	0.184	47.5
**E-factor** = $m_{waste}/m_{product}$			**6.1**
**PMI** = $(m_{reactans}+m_{reagents}+m_{solvent})/m_{product}$			**7.1**

Substance	MW	n (mmol)	weight (mg)
Substrate **1a**	260.1	0.2	52.0
PPh_3MeBr	356	0.78	277.7
LiHMDS	167.3	0.78	130.5
**THF**	72.1	24.7	1780.0
Product **2a**	258.1	0.174	44.9
**E-factor** = $m_{waste}/m_{product}$			**48.9**
**PMI** = $(m_{reactans}+m_{reagents}+m_{solvent})/m_{product}$			**49.9**

Additionally, the PMI of the model reaction on gram scale was calculated according to Equation S1, including the work-up and purification procedure.

***Equation S1.** PMI of the model reaction on gram scale.*

$$PMI = \frac{m_{starting\ material} + m_{Wittig\ Salt} + m_{base} + m_{solvent\ extration} + m_{solvent\ chromatography} + m_{silica}}{m_{product}} = 342$$

Assuming that solvents used for extraction and chromatography can be recycled, the adjusted PMI was recalculated according to Equation S2.

***Equation S2.** PMI of the model reaction on gram scale with solvent recovery.*

$$PMI = \frac{m_{starting\ material} + m_{Wittig\ Salt} + m_{base} + m_{NH4Cl\ aq.} + m_{silica}}{m_{product}} = 57$$

D.2. Green Metrics Calculation for 3a

***Table S13**. Green metrics calculation for the synthesis of **3a** (on left) and comparison with the solution approach (on right).*[8]

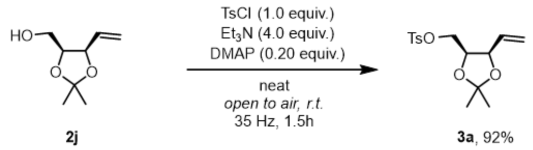

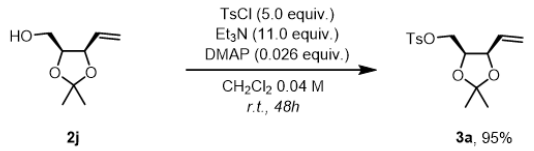

Substance	MW	n (mmol)	weight (mg)
Substrate **2j**	260.1	0.2	52.0
Tosyl Chloride	190.6	0.2	38.1
DMAP	122.2	0.04	4.9
Et_3N	101.2	0.8	81.0
**Solvent**	**0**	**0**	**0**
Product **3a**	312.4	0.184	57.5
**E-factor** = $m_{waste}/m_{product}$			**2.1**
**PMI** = $(m_{reactans}+m_{reagents}+m_{solvent})/m_{product}$			**3.1**

Substance	MW	n (mmol)	weight (mg)
Substrate **2j**	260.1	0.2	52.0
Tosyl Chloride	190.6	1	190.6
DMAP	122.2	0.5	61.1
Et_3N	101.2	2.3	232.8
**CH_2Cl_2**	**84.93**	**15.0**	**1276**
Product **3a**	312.4	0.19	59.4
**E-factor** = $m_{waste}/m_{product}$			**29.5**
**PMI** = $(m_{reactans}+m_{reagents}+m_{solvent})/m_{product}$			**30.5**

D.3. Green Metrics Calculation for 3b

***Table S14**. Green metrics calculation for the synthesis of **3b** (on left) and comparison with the solution approach (on right).*[9]

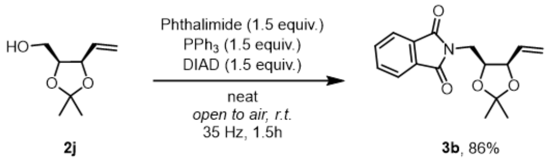

Substance	MW	n (mmol)	weight (mg)
Substrate **2j**	260.1	0.2	52.0
Phthalimide	147.1	0.3	44.1
PPh_3	262.3	0.3	78.7
DIAD	202.2	0.3	60.7
Solvent	0	0	0
Product **3b**	287.3	0.172	49.4
**E-factor** = $m_{waste}/m_{product}$			3.8
**PMI** = $(m_{reactans}+m_{reagents}+m_{solvent})/m_{product}$			4.8

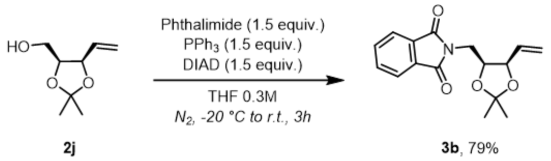

Substance	MW	n (mmol)	weight (mg)
Substrate **2j**	260.1	0.2	52.0
Phthalimide	147.1	0.3	44.1
PPh_3	262.3	0.3	78.7
DIAD	202.2	0.3	60.7
THF	72.1	7.8	560
Product **3b**	287.3	0.158	45.4
**E-factor** = $m_{waste}/m_{product}$			16.5
**PMI** = $(m_{reactans}+m_{reagents}+m_{solvent})/m_{product}$			17.5

D.4. Green Metrics Calculation for 3c

***Table S15**. Green metrics calculation for the synthesis of 3c (on left) and comparison with the solution approach (on right).*[8]

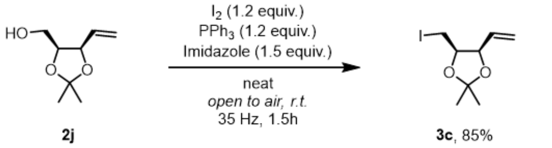

Substance	MW	n (mmol)	weight (mg)
Substrate **2j**	260.1	0.2	52.0
I_2	253.8	0.24	60.9
PPh_3	262.3	0.24	63.0
Imidazole	68.1	0.3	20.4
Solvent	0	0	0
Product **3c**	268.1	0.17	45.6
**E-factor** = $m_{waste}/m_{product}$			3.3
**PMI** = $(m_{reactans}+m_{reagents}+m_{solvent})/m_{product}$			4.3

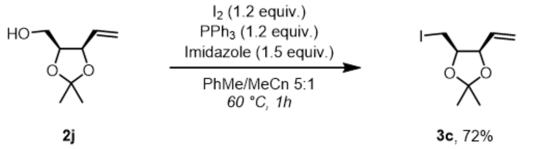

Substance	MW	n (mmol)	weight (mg)
Substrate **2j**	260.1	0.2	52.0
I_2	253.8	0.24	60.9
PPh_3	262.3	0.24	63.0
Imidazole	68.1	0.3	20.4
PhMe/MeCN			548
Product **3c**	268.1	0.144	38.6
**E-factor** = $m_{waste}/m_{product}$			18.3
**PMI** = $(m_{reactans}+m_{reagents}+m_{solvent})/m_{product}$			19.3

E. General Comparison with Solution Approach

Table S16. *Comparison between mechanochemical and solution-phase conditions for the Wittig olefination of sugars.*

Product	Yield (%) via Ball milling	Yield (%) via Solution	Solution conditions	Reference
**2a**	96	87	N_2, ylide pre-formation, 2h	[7]
**2b**	82	30	N_2, ylide pre-formation, -20°C to r.t., 24h	[10]
**2c**	90	90	N_2, 24h	[11]
**2e**	81	78	N_2, ylide pre-formation, 55h	[10]
**2f**	64	80	ylide pre-formation (-78 °C, 1h), then r.t. 16h + reflux 2h	[12]
**2g**	57	95	N_2, ylide pre-formation (2h), 0 °C to r.t., 8h	[13]
**2i**	59	89	ylide pre-formation, 0 °C to r.t., 14h	[14]
**2j**	68	70	N_2, ylide pre-formation, 0 °C to r.t., 12h	[15]
**2m**	74	98	Ar, ylide pre-formation (90 min), then r.t., 24 h	[16]

F. Characterisation Data

F.1. Starting materials

F.1.1. 2,3,4,6-Tetra-*O*-benzyl-D-*galacto*-pyranose (1e)

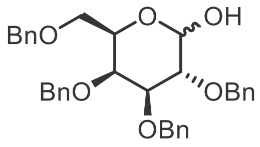

1e

Obtained 1.66 g (74%) of an anomeric mixture α:β 0.7:1 as colourless solid following general procedure A1.

^{1}H NMR (400 MHz, Chloroform-*d*) δ 7.70 – 6.87 (m, 34H), 5.32 (dd, *J* = 3.7, 2.3 Hz, 1H), 5.03 – 4.92 (m, 2.4H), 4.90 – 4.72 (m, 6.1H), 4.69 (dd, *J* = 7.5, 6.4 Hz, 0.7H), 4.66 – 4.59 (m, 1.7H), 4.51 (dd, *J* = 11.9, 2.0 Hz, 1.7H), 4.44 (dd, *J* = 11.9, 3.5 Hz, 1.7H), 4.20 (td, *J* = 6.4, 1.3 Hz, 1H), 4.07 (dd, *J* = 9.8, 3.6 Hz, 1H), 4.01 – 3.98 (m, 1H), 3.96 (dd, *J* = 9.8, 2.8 Hz, 1H), 3.91 (d, *J* = 2.9 Hz, 0.7H), 3.80 (ddd, *J* = 10.2, 7.6, 2.6 Hz, 0.7H), 3.66 – 3.59 (m, 1.4H), 3.59 – 3.49 (m, 3.4H), 3.22 (bs, 1H).

^{13}C NMR (101 MHz, Chloroform-*d*) δ 138.74, 138.71, 138.67, 138.6, 138.5, 138.4, 138.0, 137.9, 128.6, 128.5, 128.44, 128.40, 128.36, 128.35, 128.3, 128.12, 128.10, 128.05, 128.0, 127.90, 127.87, 127.74, 127.68, 127.6, 97.9, 92.0, 82.3, 80.8, 78.9, 76.7, 75.2, 74.82, 74.78, 74.7, 73.71, 73.67, 73.63, 73.58, 73.1, 73.0, 69.6, 69.1, 69.0.

Spectral data in accordance with literature.[17]

F.1.2. 2,3,4-Tri-*O*-benzyl-D-*xylo*-pyranose (1f)

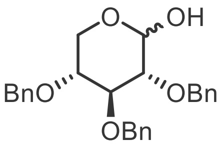

1f

Obtained 858 mg (68%) of an anomeric mixture α:β 1:0.5 as colourless solid following general procedure A1.

^{1}H NMR (600 MHz, Chloroform-*d*) δ 7.38 – 7.27 (m, 22.5H), 5.12 (d, *J* = 3.5 Hz, 1H), 4.92 – 4.87 (m, 0.5H), 4.88 – 4.83 (m, 3H), 4.79 – 4.76 (m, 1.5H), 4.72 (dd, *J* = 11.7, 1.5 Hz, 1.5H), 4.69 – 4.66 (m, 1.5H), 4.64 (d, *J* = 11.7 Hz, 1H), 4.63 (d, *J* = 11.6 Hz, 0.5H), 3.98 – 3.93 (m, 0.5H), 3.87 (t, *J* = 8.7 Hz, 1H), 3.80 (dd, *J* = 11.3, 10.1 Hz, 1H), 3.67 (dd, *J* = 11.2, 5.3 Hz, 1H), 3.65 – 3.58 (m, 1H), 3.55 (ddd, *J* = 10.3, 8.5, 5.3 Hz, 1H), 3.49 (dd, *J* = 8.9, 3.5 Hz, 1H), 3.35 – 3.25 (m, 1H), 3.21 (d, *J* = 5.5 Hz, 0.5H), 2.98 – 2.95 (m, 1H).

^{13}C NMR (151 MHz, Chloroform-*d*) δ 138.75, 138.60, 138.44, 138.34, 138.19, 137.93, 128.66, 128.62, 128.59, 128.57, 128.53, 128.23, 128.19, 128.16, 128.14, 128.11, 128.03, 127.97, 127.95, 127.92, 127.84, 127.82, 97.86, 91.62, 83.28, 82.39, 80.58, 79.55, 77.63, 77.57, 75.65, 75.63, 74.92, 73.59, 73.41, 73.35, 63.86, 60.54.

Spectral data in accordance with literature.[18]

F.1.3. 2,3,4-Tri-*O*-benzyl-D-*arabino*-pyranose (1g)

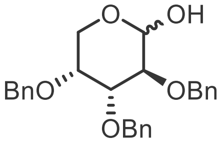

1g

Obtained 896 mg (71%) of an anomeric mixture α:β 1:0.8 as colourless, amorphous solid following general procedure A1.

^{1}H NMR (600 MHz, Chloroform-*d*) δ 7.22 (ddd, *J* = 29.7, 19.1, 5.9 Hz, 27H), 5.09 (s, 1H), 4.79 (s, 0.8H), 4.70 (d, *J* = 11.9 Hz, 0.8H), 4.64 (d, *J* = 11.5 Hz, 2H), 4.62 – 4.57 (m, 2H), 4.54 (m, 6.8H), 4.46 (dd, *J* = 19.0, 11.9 Hz, 2H), 3.99 – 3.91 (m, 1H), 3.83 – 3.77 (m, 2H), 3.75 (d, *J* = 7.9 Hz, 2H), 3.69 (d, *J* = 10.7 Hz, 2H), 3.57 – 3.51 (m, 2H).

^{13}C NMR (151 MHz, Chloroform-*d*) δ 138.4, 138.2, 138.1, 137.9, 137.7, 137.5, 128.56, 128.54, 128.51, 128.49, 128.44, 128.42, 128.10, 128.05, 128.03, 128.01, 127.97, 127.9, 127.8, 127.8, 127.7, 127.7, 93.8, 92.1, 76.7, 76.3, 76.2, 75.5, 73.8, 73.7, 73.0, 72.9, 72.7, 72.1, 71.6, 61.0, 58.4.

Spectral data in accordance with literature.[19]

F.1.4. 2,3-*O*-Isopropylidene-D-*ribo*-furanose (1i)

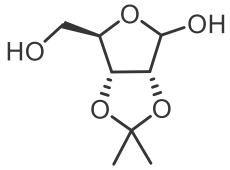

1i

Obtained 1.48 g (78%) of an anomeric mixture α:β 0.1:1 as colourless oil following general procedure A2.

^{1}H NMR (400 MHz, Chloroform-*d*) δ 5.42 (d, *J* = 4.2 Hz, 0.1H), 5.39 (d, *J* = 5.8 Hz, 1H), 5.25 – 5.09 (m, 1H), 4.80 (d, *J* = 6.0 Hz, 1H), 4.71 (dd, *J* = 6.6, 2.3 Hz, 0.1H), 4.63 (dd, *J* = 6.7, 4.2 Hz, 0.1H), 4.56 (d, *J* = 5.9 Hz, 1H), 4.40 – 4.35 (m, 1H), 4.18 – 4.13 (m, 0.2H), 3.93 (bs, 1H), 3.79 – 3.59 (m, 2.2H), 2.75 – 2.56 (m, 0.1H), 2.30 (bs, 0.1H), 1.56 (s, 0.3H), 1.47 (s, 3H), 1.38 (s, 0.3H), 1.30 (s, 3H).

^{13}C NMR (101 MHz, Chloroform-*d*) δ 114.4, 112.3, 103.0, 97.1, 87.9, 86.9, 81.8, 81.6, 81.2, 79.6, 63.7, 63.4, 26.5, 26.3, 24.85, 24.83.

Spectral data in accordance with literature.[4]

F.1.5. 2,3-*O*-Isopropylidene-L-*erythro*-furanose (1j)

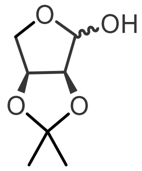

1j

Obtained 183.5 mg (38%) of an anomeric mixture α:β 0.15:1 as colourless oil following general procedure A3.

^{1}H NMR (400 MHz, Chloroform-*d*) δ 5.40 (d, *J* = 2.4 Hz, 1H), 4.98 (ddd, *J* = 11.5, 3.6, 0.7 Hz, 0.15H), 4.82 (dd, *J* = 5.9, 3.5 Hz, 1H), 4.75 (ddd, *J* = 6.2, 3.8, 1.0 Hz, 0.15H), 4.56 (d, *J* = 5.9 Hz, 1H), 4.48 (dd, *J* = 6.2, 3.6 Hz, 0.15H), 4.08 – 4.03 (m, 1H), 4.00 (dt, *J* = 10.3, 0.7 Hz, 1H), 3.98 – 3.95 (m, 0.15H), 3.93 (d, *J* = 11.8 Hz, 0.15H), 3.53 (dd, *J* = 11.0, 3.7 Hz, 0.15H), 3.15 (dt, *J* = 3.2, 1.6 Hz, 1H), 1.53 (d, *J* = 0.8 Hz, 0.45H), 1.45 (d, *J* = 0.8 Hz, 3H), 1.37 (d, *J* = 0.8 Hz, 0.45H), 1.31 (d, *J* = 0.8 Hz, 3H).

^{13}C NMR (101 MHz, Chloroform-*d*) δ 113.6, 112.4, 101.9, 97.6, 85.3, 80.1, 79.7, 78.4, 72.0, 67.7, 26.3, 26.1, 25.0, 24.8.

Spectral data in accordance with literature.[20]

F.2. Olefination products

F.2.1. 1,2-Dideoxy-3,4;6,7-di-*O*-isopropylidene-D-*manno*-hept-1-enitol (2a)

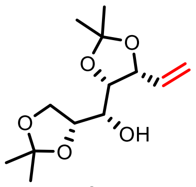

2a

Obtained 48.6 mg (96%) as colourless oil from **1a** following general procedure B1.

HRMS calcd. for $[C_{13}H_{22}O_5]Na$: 281.1359, found 281.1365.

^{1}H NMR (400 MHz, Chloroform-*d*) δ 6.08 (ddd, *J* = 17.3, 10.3, 7.8 Hz, 1H), 5.39 (dt, *J* = 17.3, 1.3 Hz, 1H), 5.32 (dq, *J* = 10.2, 0.8 Hz, 1H), 4.69 (t, *J* = 7.6 Hz, 1H), 4.38 (dd, *J* = 7.4, 1.3 Hz, 1H), 4.12 – 4.04 (m, 1H), 4.03 – 3.95 (m, 2H), 3.44 (td, *J* = 8.0, 1.2 Hz, 1H), 2.21 (d, *J* = 8.1 Hz, 1H), 1.52 (s, 3H), 1.40 (s, 3H), 1.37 (s, 3H), 1.33 (s, 3H).

^{13}C NMR (101 MHz, Chloroform-*d*) δ 134.3, 119.8, 109.4, 108.8, 79.3, 76.8, 76.2, 70.7, 67.2, 26.9, 26.8, 25.4, 24.6.

Spectral data in accordance with literature.[7]

F.2.2. 1,2-Dideoxy-3,4,5,7-tetra-*O*-benzyl-D-*manno*-hept-1-enitol (2b)

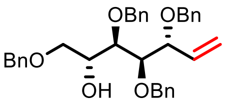

2b

Obtained 79.8 mg (82%) as colourless oil from **1b** following slightly modified general procedure B2, using 3.5 equiv. of methyltriphenylphosphonium bromide and 3.2 equiv. of LiHMDS and milling for 1 h 30 min.

HRMS calcd. for $[C_{35}H_{38}O_5]Cl^-$: 573.2413, found 573.2394.

^{1}H NMR (400 MHz, Chloroform-*d*) δ 7.35 – 7.13 (m, 20H), 6.01 – 5.87 (m, 1H), 5.46 – 5.34 (m, 2H), 4.69 (d, *J* = 11.2 Hz, 1H), 4.61 (d, *J* = 7.2 Hz, 1H), 4.58 (d, *J* = 7.7 Hz, 1H), 4.52 (d, *J* = 11.5 Hz, 1H), 4.50 – 4.42 (m, 3H), 4.21 (d, *J* = 11.6 Hz, 1H), 4.12 – 4.05 (m, 1H), 4.03 – 3.94 (m, 1H), 3.88 – 3.80 (m, 2H), 3.61 (dd, *J* = 9.6, 3.4 Hz, 1H), 3.54 (dd, *J* = 9.7, 5.4 Hz, 1H), 2.62 (d, *J* = 5.4 Hz, 1H).

^{13}C NMR (101 MHz, Chloroform-*d*) δ 138.6, 138.51, 138.49, 138.2, 136.4, 128.6, 128.5, 128.4, 128.3, 128.02, 127.98, 127.9, 127.8, 127.7, 127.65, 127.63, 119.9, 81.0, 80.4, 78.7, 74.5, 74.1, 73.5, 71.4, 70.3, 70.1.

Spectral data in accordance with literature.[10]

F.2.3. 1,2-Dideoxy-3,4,5,7-tetra-*O*-benzyl-D-*gluco*-hept-1-enitol (2c)

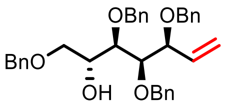

2c

Obtained 87 mg (81%) as colourless oil from **1c** following general procedure B2, using NaHMDS.

HRMS calcd. for $[C_{35}H_{38}O_5]Na$: 561.2611, found 561.2633.

^{1}H NMR (400 MHz, Chloroform-*d*) δ 7.31 – 7.15 (m, 20H), 5.80 (ddd, *J* = 17.3, 10.3, 7.9 Hz, 1H), 5.30 – 5.20 (m, 2H), 4.68 (s, 2H), 4.58 (d, *J* = 11.8 Hz, 1H), 4.42 (d, *J* = 12.0 Hz, 1H), 4.38 – 4.25 (m, 4H), 4.08 – 3.98 (m, 2H), 3.76 – 3.70 (m, 2H), 3.43 (qd, *J* = 9.4, 6.3 Hz, 2H), 2.95 (d, *J* = 5.4 Hz, 1H).

^{13}C NMR (101 MHz, Chloroform-*d*) δ 138.4, 138.34, 138.30, 138.2, 135.9, 128.48, 128.46, 128.2, 128.14, 128.13, 127.9, 127.85, 127.81, 127.7, 119.3, 82.3, 80.9, 76.7, 75.4, 73.3, 73.2, 71.4, 70.5, 69.8.

Spectral data in accordance with literature.[21]

F.2.4. 1,2-Dideoxy-5,7-*O*-(phenylmethyl)-D-*gluco*-hept-1-enitol (2d)

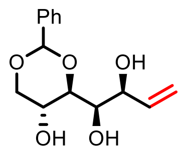

2d

Obtained 22 mg (41%) as colourless solid (m.p.: 136.1–138.5 °C) from **1d** following slightly modified general procedure B1, using 7 equiv. of methyltriphenylphosphonium bromide and 6 equiv. of KO^{t}Bu.

HRMS calcd. for $[C_{14}H_{18}O_5]$Na: 289.1046, found 289.1058.

^{1}H NMR (400 MHz, Methanol-d_4) δ 7.51 – 7.45 (m, 2H), 7.38 – 7.31 (m, 3H), 5.94 (ddd, *J* = 17.3, 10.4, 7.0 Hz, 1H), 5.45 (s, 1H), 5.33 (ddd, *J* = 17.2, 1.9, 1.2 Hz, 1H), 5.22 (ddd, *J* = 10.4, 1.9, 1.0 Hz, 1H), 4.29 – 4.21 (m, 2H), 3.97 (ddd, *J* = 10.2, 9.5, 5.4 Hz, 1H), 3.76 (dd, *J* = 7.9, 1.9 Hz, 1H), 3.65 (dd, *J* = 9.4, 1.9 Hz, 1H), 3.58 (t, *J* = 10.5 Hz, 1H).

^{13}C NMR (101 MHz, Methanol-d_4) δ 139.5, 138.9, 129.8, 129.0, 127.3, 117.4, 102.2, 82.6, 75.3, 73.1, 72.5, 61.6.

F.2.5. 1,2-Dideoxy-3,4,5,7-tetra-*O*-benzyl-D-*galacto*-hept-1-enitol (2e)

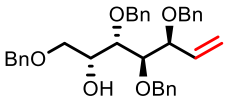

2e

Obtained 97 mg (90%) as colourless oil from **1e** following general procedure B2, using NaHMDS.

HRMS calcd. for $[C_{35}H_{38}O_5]$Na: 561.2611, found 561.2614.

^{1}H NMR (400 MHz, Chloroform-*d*) δ 7.58 – 6.92 (m, 20H), 5.75 (ddd, *J* = 17.2, 10.4, 7.7 Hz, 1H), 5.24 – 5.12 (m, 2H), 4.73 (d, *J* = 11.4 Hz, 1H), 4.59 (d, *J* = 11.4 Hz, 1H), 4.53 (d, *J* = 11.4 Hz, 1H), 4.49 (d, *J* = 11.6 Hz, 1H), 4.44 (d, *J* = 11.6 Hz, 1H), 4.39 (d, *J* = 1.9 Hz, 2H), 4.30 (d, *J* = 11.7 Hz, 1H), 4.10 (dd, *J* = 7.7, 6.0 Hz, 1H), 3.94 (p, *J* = 5.3 Hz, 1H), 3.71 – 3.61 (m, 2H), 3.53 – 3.47 (m, 2H), 2.78 (d, *J* = 5.5 Hz, 1H).

^{13}C NMR (101 MHz, Chloroform-d) δ 138.52, 138.44, 138.37, 138.15, 135.45, 128.59, 128.51, 128.48, 128.42, 128.37, 128.02, 127.95, 127.90, 127.81, 127.75, 127.62, 127.01, 119.18, 81.52, 81.47, 78.46, 74.83, 73.42, 73.30, 71.28, 70.75, 70.5.

Spectral data in accordance with literature. [10]

F.2.6. 1,2-Dideoxy-3,4,5-tri-*O*-benzyl-D-*xylo*-hex-1-enitol (2f)

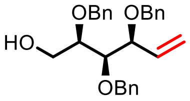

2f

Obtained 54 mg (64%) as colourless oil from **1f** following the slightly modified general procedure B2, using LiHMDS.

HRMS calcd. for $[C_{27}H_{30}O_4]$Na: 441.2036, found 441.2047.

^{1}H NMR (400 MHz, Chloroform-*d*) δ 7.40 – 7.28 (m, 15H), 5.91 (dddd, *J* = 17.1, 10.8, 7.4, 1.4 Hz, 1H), 5.39 – 5.27 (m, 2H), 4.76 (s, 2H), 4.68 – 4.62 (m, 3H), 4.40 (dd, *J* = 11.7, 1.4 Hz, 1H), 4.17 – 4.08 (m, 1H), 3.79 – 3.64 (m, 3H), 3.61 – 3.52 (m, 1H), 2.21 (d, *J* = 4.5 Hz, 1H).

^{13}C NMR (101 MHz, Chloroform-d) δ 138.6, 138.4, 138.1, 135.2, 128.53, 128.50, 128.46, 128.1, 128.0, 127.9, 127.81, 127.79, 119.0, 81.8, 80.6, 79.7, 74.9, 72.9, 70.8, 61.6

Spectral data in accordance with literature.[17]

F.2.7. 1,2-Dideoxy-3,4,5-tri-*O*-benzyl-D-*arabino*-hex-1-enitol (2g)

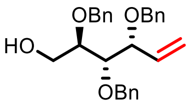

2g

Obtained 48 mg (57%) as colourless oil from **1g** following general procedure B2, using NaHMDS.

HRMS calcd. for $[C_{27}H_{30}O_4]Na$: 441.2036, found 441.2040.

^{1}H NMR (400 MHz, Chloroform-*d*) δ 7.47 – 7.16 (m, 15H), 5.92 (ddd, *J* = 17.3, 10.3, 7.8 Hz, 1H), 5.41 – 5.26 (m, 2H), 4.74 (d, *J* = 1.1 Hz, 2H), 4.66 (d, *J* = 11.8 Hz, 1H), 4.48 (d, *J* = 11.4 Hz, 1H), 4.37 (dd, *J* = 11.6, 8.5 Hz, 2H), 4.05 (ddt, *J* = 7.9, 4.2, 1.0 Hz, 1H), 3.81 (ddd, *J* = 6.8, 3.9, 2.5 Hz, 2H), 3.78 – 3.69 (m, 2H), 2.20 (dd, *J* = 6.8, 5.9 Hz, 1H).

^{13}C NMR (101 MHz, Chloroform-*d*) δ 138.4, 138.32, 138.25, 128.6, 128.48, 128.47, 128.4, 128.2, 127.91, 127.88, 127.8, 81.7, 80.8, 79.0, 75.2, 71.9, 70.6, 61.0.

Spectral data in accordance with literature.[13]

F.2.8. 1,2-Dideoxy-D-*arabino*-hex-1-enitol (2h)

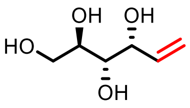

2h

Obtained 17.5 mg (59%) as colourless solid (m.p.: 145.0–147.1 °C) from **1h** following general procedure B1, using KO^{t}Bu (7.5 equiv.) and PPh_3MeBr (8.2 equiv.). Analytical sample repurified via acetylation and deacetylation.

HRMS calcd. for $[C_6H_{12}O_4][HCOO]^-$: 193.0712, found 193.0716.

^{1}H NMR (600 MHz, Methanol-*d*$_4$) δ 6.00 (ddd, *J* = 17.3, 10.6, 5.9 Hz, 1H), 5.32 (dt, *J* = 17.2, 1.7 Hz, 1H), 5.17 (dt, *J* = 10.6, 1.7 Hz, 1H), 4.33 (ddt, *J* = 5.8, 2.8, 1.5 Hz, 1H), 3.77 (dd, *J* = 11.3, 3.5 Hz, 1H), 3.69 (ddd, *J* = 7.8, 6.0, 3.5 Hz, 1H), 3.61 (dd, *J* = 11.3, 6.0 Hz, 1H), 3.45 (dd, *J* = 7.8, 2.7 Hz, 1H).

^{13}C NMR (151 MHz, Methanol-*d*$_4$) δ 138.7, 114.4, 73.9, 71.8, 71.6, 63.5.

Spectral data in accordance with literature.[22]

F.2.9. 1,2-Dideoxy-3,4-*O*-(1-methylethylidene)-D-*ribo*-hex-1-enitol (2i)

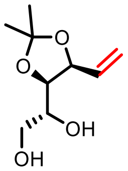

2i

Obtained 22 mg (59%) as colourless oil from **1i** following general procedure B1, using KO^{t}Bu (3.5 equiv.) and PPh_3MeBr (4.2 equiv.).

HRMS calcd. for $[C_9H_6O_4]Na$: 211.0941, found 211.0939.

^{1}H NMR (400 MHz, Chloroform-*d*) δ 6.01 (ddd, *J* = 17.3, 10.4, 7.1 Hz, 1H), 5.47 (dt, *J* = 17.2, 1.4 Hz, 1H), 5.34 (dt, *J* = 10.4, 1.3 Hz, 1H), 4.74 – 4.68 (m, 1H), 4.14 – 4.07 (m, 1H), 3.86 – 3.78 (m, 1H), 3.76 – 3.68 (m, 2H), 1.98 (bs, 2H), 1.47 (s, 3H), 1.37 (d, J = 0.5 Hz, 3H).

^{13}C NMR (101 MHz, Chloroform-*d*) δ 133.9, 118.8, 109.2, 78.7, 78.3, 69.9, 64.5, 27.9, 25.4.

Spectral data in accordance with literature.[14]

F.2.10. 1,2-Dideoxy-3,4-*O*-(1-methylethylidene)-L-*erythro*-pent-1-enitol (2j)

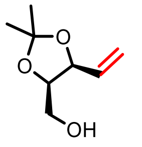

2j

Obtained 22 mg (68%) as colourless oil from **1j** following general procedure B1.

HRMS calcd. for [$C_9H_6O_4$]Na: 211.0941, found 211.0939.

^{1}H NMR (400 MHz, Chloroform-*d*) δ 5.87 (ddd, *J* = 17.6, 10.4, 7.4 Hz, 1H), 5.39 (dt, *J* = 17.2, 1.3 Hz, 1H), 5.28 (dt, *J* = 10.3, 1.1 Hz, 1H), 4.65 (t, *J* = 7.1 Hz, 1H), 4.26 (dd, *J* = 11.8, 6.2 Hz, 1H), 3.58 (t, *J* = 5.7 Hz, 2H), 1.93 – 1.83 (m, 1H), 1.51 (s, 3H), 1.39 (s, 3H).

^{13}C NMR (101 MHz, Chloroform-*d*) δ 133.1, 119.1, 109.0, 78.4, 78.4, 62.2, 27.9, 25.4.

Spectral data in accordance with literature.[15]

F.2.11. (1,1-$^2H_2$)-1,2-Dideoxy-3,4;6,7-di-*O*-isopropylidene-D-*manno*-hept-1-enitol (2k)

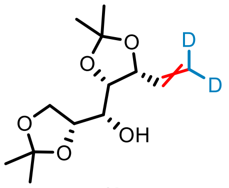

2k

Obtained 48 mg (93%) as colourless oil from **1a** following general procedure B1 with D% = 75%. For D% = 84%: starting material **1a** was evaporated from CD_3OD (3×) prior to use.

With the available HRMS equipment, no HRMS data for compound **2k** could be obtained.

^{1}H NMR (400 MHz, Chloroform-*d*) δ 6.08 – 5.97 (m, 1H), 5.32 (dd, *J* = 17.2, 1.0 Hz, 1H), 5.27 – 5.23 (m, 1H), 4.64 (t, *J* = 7.6 Hz, 1H), 4.32 (dd, *J* = 7.5, 1.3 Hz, 1H), 4.07 – 3.98 (m, 1H), 3.98 – 3.90 (m, 2H), 3.44 – 3.34 (m, 1H), 2.14 (d, *J* = 8.1 Hz, 1H), 1.46 (s, 3H), 1.34 (s, 3H), 1.32 (s, 3H), 1.28 (s, 3H). *D% = 75%

^{1}H NMR (400 MHz, Chloroform-*d*) δ 6.14 – 6.04 (m, 1H), 5.38 (dd, *J* = 17.2, 1.0 Hz, 0.16H), 5.31 (dd, *J* = 10.3, 0.9 Hz, 0.16H), 4.70 (t, *J* = 7.6 Hz, 1H), 4.38 (dd, *J* = 7.4, 1.3 Hz, 1H), 4.11 – 4.05 (m, 1H), 4.03 – 3.96 (m, 2H), 3.50 – 3.40 (m, 1H), 2.20 (d, *J* = 8.1 Hz, 1H), 1.52 (s, 3H), 1.40 (s, 3H), 1.38 (s, 3H), 1.34 (s, 3H). *D% = 84%

^{13}C NMR (101 MHz, Chloroform-*d*) δ 134.2, 134.1, 119.9 – 119.3 (m), 109.5, 108.8, 79.2, 76.8, 76.2, 70.7, 67.3, 26.9, 26.8, 25.4, 24.6.

F.2.12. (*R*)-[(*R*)-2,2-Dimethyl-1,3-dioxolan-4-yl][(4*S*,5*R*)-2,2-dimethyl-5-(2-methylprop-1-enyl)-1,3-dioxolan-4-yl]methanol (*manno*) (2l)

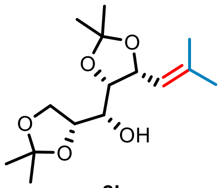

2l

Obtained 26.3 mg (46%) as colourless oil from **1a** following slightly modified general procedure B2, using NaHMDS.

HRMS calcd. for [$C_{15}H_{26}O_5$]Na: 309.1672, found 309.1680.

^{1}H NMR (400 MHz, Chloroform-*d*) δ 5.47 (ddt, *J* = 8.7, 2.7, 1.3 Hz, 1H), 5.02 (dd, *J* = 8.5, 7.5 Hz, 1H), 4.32 (dd, *J* = 7.3, 1.3 Hz, 1H), 4.12 – 4.05 (m, 1H), 4.04 – 3.97 (m, 2H), 3.44 (t, *J* = 6.9 Hz, 1H), 2.18 (d, *J* = 7.8 Hz, 1H), 1.80 (d, *J* = 1.1 Hz, 3H), 1.71 (d, *J* = 1.3 Hz, 3H), 1.51 (s, 3H), 1.41 (s, 3H), 1.38 (s, 3H), 1.35 (s, 3H).

^{13}C NMR (101 MHz, Chloroform-*d*) δ 139.3, 120.3, 109.3, 108.1, 76.4, 76.3, 74.5, 70.9, 67.1, 27.0, 26.8, 26.2, 25.4, 24.6, 18.5.

F.2.13. (2*R*,3*R*,4*R*,5*R*)-3-Hydroxy-1,2;4,5-di-*O*-isopropylidene-6-decene (*manno*) (2m)

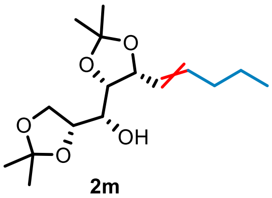

2m
E:Z (1:19)

Obtained 45 mg (74%) as colourless oil from **1a** following general procedure B1.

HRMS calcd. for $[C_{16}H_{28}O_5]Na$: 323.1829, found 323.1838.

^{1}H NMR (400 MHz, Chloroform-*d*) δ 5.76 – 5.62 (m, 2H), 5.09 (t, *J* = 7.4 Hz, 1H), 4.37 (dd, *J* = 7.5, 1.2 Hz, 1H), 4.12 – 4.05 (m, 1H), 4.04 – 3.97 (m, 2H), 3.43 (td, *J* = 8.1, 1.2 Hz, 1H), 2.16 (d, *J* = 8.4 Hz, 1H), 2.14 – 1.98 (m, 2H), 1.52 (s, 3H), 1.47-1.32 (m, 2H), 1.42 (s, 3H), 1.40 – 1.37 (m, 3H), 1.34 (s, 3H), 0.91 (t, *J* = 7.3 Hz, 3H). *Only *Z* isomer data

^{13}C NMR (101 MHz, Chloroform-*d*) δ 133.1, 119.1, 109.0, 78.4, 78.4, 62.2, 27.9, 25.4. *Only *Z* isomer data

Spectral data in accordance with literature.[16]

F.2.14. (*Z*)-5-[(4*R*,5*S*)-5-{(*R*)-[(*R*)-2,2-dimethyl-1,3-dioxolan-4-yl]hydroxymethyl}-2,2-dimethyl-1,3-dioxolan-4-yl]pent-4-enenitrile (*manno*) (2n)

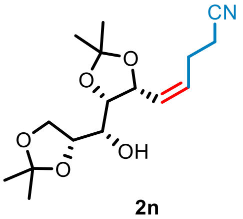

2n

Obtained 53.5 mg (90%) as colourless oil from **1a** following general procedure B1.

HRMS calcd. for $[C_{16}H_{25}O_5]Na$: 334.1625, found 334.1637.

^{1}H NMR (400 MHz, Chloroform-*d*) δ 5.95 – 5.86 (m, 1H), 5.75 – 5.64 (m, 1H), 5.06 – 4.98 (m, 1H), 4.39 (d, *J* = 7.5 Hz, 1H), 4.11 – 4.03 (m, 1H), 4.03 – 3.94 (m, 2H), 3.42 (t, *J* = 8.1 Hz, 1H), 2.58 – 2.34 (m, 4H), 2.17 (d, *J* = 8.7 Hz, 1H), 1.51 (s, 3H), 1.41 (s, 3H), 1.37 (s, 3H), 1.33 (s, 3H).

^{13}C NMR (101 MHz, Chloroform-*d*) δ 130.3, 129.3, 118.9, 109.4, 108.9, 76.7, 76.2, 73.1, 70.7, 67.1, 26.9, 26.7, 25.4, 24.5, 23.9, 17.5.

F.2.15. (*R*)-((4*S*,5*R*)-5-((*E*)-Buta-1,3-dien-1-yl)-2,2-dimethyl-1,3-dioxolan-4-yl)((*R*)-2,2-dimethyl-1,3-dioxolan-4-yl)methanol (*manno*) (2o)

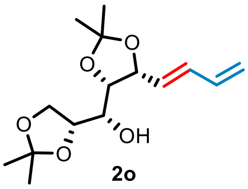

2o
E:Z (9:1)

Obtained 50 mg (88%) as colourless oil as *E*/*Z* mixture > 9:1 from **1a** following general procedure B1.

HRMS calcd. for $[C_{15}H_{24}O_5]Na$: 307.1516, found 307.1511.

^{1}H NMR (400 MHz, Chloroform-*d*) δ 6.48 – 6.28 (m, 2H), 5.93 (dd, *J* = 14.5, 8.3 Hz, 1H), 5.31 – 5.19 (m, 1H), 5.16 (dd, *J* = 9.5, 1.8 Hz, 1H), 4.74 (t, *J* = 7.9 Hz, 1H), 4.37 (dd, *J* = 7.4, 1.4 Hz, 1H), 4.13 – 4.04 (m, 1H), 4.04 – 3.94 (m, 2H), 3.53 – 3.31 (m, 1H), 2.20 (d, *J* = 8.3 Hz, 1H), 1.52 (s, 3H), 1.40 (s, 3H), 1.38 (s, 3H), 1.34 (s, 3H). *Only *E* isomer data

^{13}C NMR (101 MHz, Chloroform-*d*) δ 136.1, 136.0, 128.8, 119.0, 109.5, 108.8, 78.66, 77.0, 76.2, 70.8, 67.2, 26.9, 26.8, 25.4, 24.6. *Only *E* isomer data

F.2.16. 1,2-Dideoxy-3,4;6,7-di-*O*-isopropylidene-1-phenyl-D-*manno*-hept-1-enitol (2p)

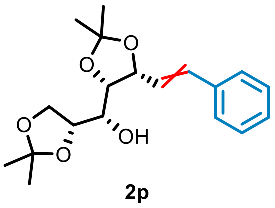

2p

Obtained 62 mg (92%) as colourless oil as *E/Z* mixture 1:1.2 from **1a** following general procedure B1.

HRMS calcd. for $[C_{19}H_{26}O_5]Na$: 357.1672, found 357.1680.

^{1}H NMR (400 MHz, Chloroform-*d*) δ 7.33 – 7.06 (m, 11H), 6.69 (d, *J* = 11.7 Hz, 1.2H), 6.59 (d, *J* = 15.9 Hz, 1H), 6.35 (dd, *J* = 15.9, 8.2 Hz, 1H), 5.89 (dd, *J* = 11.7, 8.9 Hz, 1.2H), 5.02 (ddd, *J* = 8.8, 7.7, 1.1 Hz, 1.2H), 4.81 – 4.74 (m, 1H), 4.43 (dd, *J* = 7.7, 0.7 Hz, 1.2H), 4.33 (dd, *J* = 7.5, 1.2 Hz, 1H), 4.02 – 3.96 (m, 2.2H), 3.95 – 3.84 (m, 4.4H), 3.47 – 3.35 (m, 2.2H), 2.19 (d, *J* = 9.1 Hz, 1.2H), 2.16 (d, *J* = 8.4 Hz, 1H), 1.46 (s, 3H), 1.44 (s, 3.6H), 1.34 (s, 3H), 1.28 – 1.22 (m, 16.8H). **E/Z* isomer mixture in ratio 1:1.2

^{13}C NMR (101 MHz, Chloroform-*d*) δ 136.3, 136.2, 135.2, 134.0, 128.7, 128.51, 128.49, 128.3, 127.9, 127.7, 127.0, 125.1, 109.5, 109.4, 108.8, 79.2, 77.14, 77.08, 76.29, 76.26, 74.1, 70.9, 70.8, 67.25, 67.19, 27.0, 26.9, 26.84, 26.76, 25.42, 25.39, 24.6, 24.3. **E/Z* isomer mixture

F.2.17. 1-(2,4-Dichlorophenyl)-1,2-dideoxy-3,4;6,7-di-*O*-isopropylidene-D-*manno*-hept-1-enitol (2q)

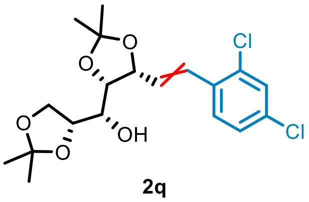

2q

Obtained 18.5 mg (23%) as colourless oil as *E/Z* mixture > 1:3 from **1a** following general procedure B2.

HRMS calcd. for $[C_{19}H_{24}Cl_2O_5]Na$: 425.0893, found 425.0915.

^{1}H NMR (600 MHz, Chloroform-*d*) δ 7.52 (d, *J* = 8.5 Hz, 1H), 7.43 (d, *J* = 2.1 Hz, 2.8H), 7.37 (d, *J* = 2.1 Hz, 1H), 7.22 (td, *J* = 8.1, 2.1 Hz, 3.8H), 7.14 (d, *J* = 8.2 Hz, 2.8H), 7.00 (d, *J* = 15.8 Hz, 1H), 6.79 (d, *J* = 11.6 Hz, 2.8H), 6.44 (dd, *J* = 15.9, 8.2 Hz, 1H), 6.16 (dd, *J* = 11.6, 9.4 Hz, 2.8H), 4.93 – 4.89 (m, 1H), 4.87 (ddd, *J* = 9.1, 7.7, 1.1 Hz, 2.8H), 4.48 (dd, *J* = 7.5, 1.1 Hz, 1H), 4.44 – 4.40 (m, 2.8H), 4.13 – 4.08 (m, 3.8H), 4.05 – 3.97 (m, 7.6H), 3.56 – 3.50 (m, 2.8H), 3.49 – 3.44 (m, 1H), 2.29 (d, *J* = 9.2 Hz, 2.8H), 2.26 (d, *J* = 8.8 Hz, 1H), 1.57 (s, 3H), 1.54 (s, 8.4H), 1.45 (s, 3H), 1.38 (s, 11.4H), 1.35 (s, 19.8H). **E/Z* isomer mixture in ratio 1:2.8

^{13}C NMR (151 MHz, Chloroform-*d*) δ 134.7, 134.4, 134.3, 133.9, 133.1, 130.82, 130.76, 129.92, 129.86, 129.7, 129.6, 129.1, 128.3, 127.5, 126.9, 109.58, 109.56, 109.10, 109.06, 78.9, 76.88, 76.87, 76.3, 76.2, 73.8, 70.8, 70.7, 67.24, 67.22, 26.99, 26.96, 26.9, 26.8, 25.41, 25.37, 24.6, 24.4. **E/Z* isomer mixture

F.2.18. (*R*)-((*R*)-2,2-Dimethyl-1,3-dioxolan-4-yl)((4*S*,5*R*)-5-(2-methoxyvinyl)-2,2-dimethyl-1,3-dioxolan-4-yl)methanol (*manno*) (2r)

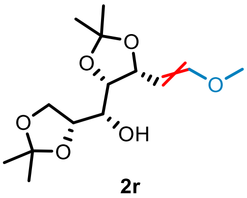

2r
E:Z (1:2)

Obtained 56 mg (97%) as colourless oil as E/Z mixture > 1:2 from **1a** following general procedure B1.

HRMS calcd. for $[C_{14}H_{24}O_6]Na$: 311.1465, found 311.1470.

^{1}H NMR (400 MHz, Chloroform-*d*) δ 6.64 (d, *J* = 12.7 Hz, 0.5H, Z), 6.13 (dd, *J* = 6.3, 1.1 Hz, 1H, E), 5.36 – 5.20 (m, 1H, E), 5.05 (dd, *J* = 12.7, 9.6 Hz, 0.5H, Z), 4.74 (dd, *J* = 8.7, 6.3 Hz, 1H, E), 4.66 (dd, *J* = 9.6, 7.4 Hz, 0.5H, Z), 4.34 (dd, *J* = 7.3, 1.4 Hz, 1H,E), 4.29 (dd, *J* = 7.5, 1.2 Hz, 0.5H, Z), 4.13 – 3.96 (m, 4.5H, E/Z), 3.63 (d, *J* = 0.6 Hz, 3H, E), 3.60 (s, 1.5H, Z), 3.52 – 3.44 (m, 1.5H, E/Z), 2.20 (d, *J* = 8.7 Hz, 0.5H, Z), 2.16 (d, *J* = 8.0 Hz, 1H, E), 1.50 (s, 4.5H), 1.39 (s, 9H), 1.34 (s, 4.5H). **E/Z* isomer mixture in ratio 1:2

^{13}C NMR (101 MHz, Chloroform-*d*) δ 153.2, 150.3, 109.4, 109.3, 108.0, 107.9, 102.1, 98.1, 76.34, 76.29, 76.26, 76.1, 71.4, 71.0, 70.9, 67.2, 67.0, 60.2, 56.3, 27.0, 26.9, 26.8, 25.5, 25.4, 24.5, 24.4. **E/Z* isomer mixture

F.2.19. (*R*)-((4*S*,5*R*)-5-(2-(1*H*-benzo[*d*][1,2,3]triazol-1-yl)vinyl)-2,2-dimethyl-1,3-dioxolan-4-yl)((*R*)-2,2-dimethyl-1,3-dioxolan-4-yl)methanol (*manno*) (2s)

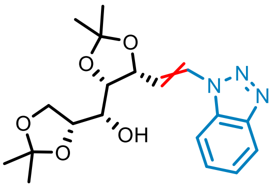

2s, 81%
E:Z (1:1.2)

Obtained 61 mg (81%) (separable isomers) from **1a** following general procedure B1.

***E*-Isomer**

Pale yellow solid (m.p.: 115.4–117.9 °C). HRMS calcd. for $[C_{19}H_{25}N_3O_6]Na$: 398.1686, found 398.1693.

^{1}H NMR (400 MHz, Chloroform-*d*) δ 8.10 – 8.06 (m, 1H), 7.61 – 7.54 (m, 2H), 7.43 (ddd, *J* = 8.1, 6.2, 1.8 Hz, 1H), 7.21 (dd, *J* = 9.3, 1.5 Hz, 1H), 6.10 (dd, *J* = 9.3, 7.7 Hz, 1H), 5.63 (td, *J* = 7.8, 1.5 Hz, 1H), 4.98 – 4.93 (m, 1H), 4.09 – 3.99 (m, 3H), 3.62 (bs, 1H), 2.23 (bs, 1H), 1.57 (s, 3H), 1.41 (s, 3H), 1.34 (s, 3H), 1.33 (s, 3H).

^{13}C NMR (101 MHz, Chloroform-*d*) δ 145.6, 132.5, 128.5, 124.9, 123.0, 120.7, 120.3, 109.5, 109.5, 109.1, 77.7, 76.7, 74.5, 70.8, 66.8, 26.9, 26.7, 25.5, 24.2.

***Z*-Isomer**

Colourless solid (m.p.: 112.7–114.9 °C). HRMS calcd. for $[C_{19}H_{25}N_3O_6]Na$: 398.1686, found 398.1689.

^{1}H NMR (400 MHz, Chloroform-*d*) δ 8.09 (d, *J* = 8.4 Hz, 1H), 7.77 (d, *J* = 8.4 Hz, 1H), 7.69 (d, *J* = 14.5 Hz, 1H), 7.46 – 7.40 (m, 1H), 6.74 (dd, *J* = 14.5, 8.2 Hz, 1H), 5.01 – 4.95 (m, 1H), 4.55 (dd, *J* = 7.5, 1.5 Hz, 1H), 4.12 (dd, *J* = 8.0, 6.1 Hz, 1H), 4.06 (dt, *J* = 8.1, 5.4 Hz, 1H), 3.99 (dd, *J* = 8.1, 5.2 Hz, 1H), 3.57 (t, *J* = 7.8 Hz, 1H), 2.42 – 2.30 (m, 1H), 1.60 (s, 3H), 1.48 (s, 3H), 1.37 (s, 3H), 1.35 (s, 3H).

^{13}C NMR (101 MHz, Chloroform-*d*) δ 146.6, 131.5, 128.7, 127.3, 124.9, 120.5, 116.8, 110.7, 109.7, 109.3, 77.1, 76.8, 76.3, 70.9, 67.4, 26.9, 25.4, 24.7.

F.2.20. (*E*)-1-Chloro-1,2-dideoxy-3,4;6,7-di-*O*-isopropylidene-D-*manno*-hept-1-enitol (2t)

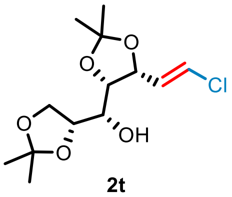

2t

Obtained 19 mg (32%) as colourless oil from **1a** following general procedure B1.

HRMS calcd. for $[C_{13}H_{21}ClO_5]Na$: 315.0970, found 315.0973.

^{1}H NMR (400 MHz, Chloroform-*d*) δ 6.34 (dd, *J* = 13.4, 0.6 Hz, 1H), 6.23 (dd, *J* = 13.3, 8.4 Hz, 1H), 4.70 (t, *J* = 7.8 Hz, 1H), 4.38 (dd, *J* = 7.4, 1.7 Hz, 1H), 4.13 – 4.07 (m, 1H), 4.03 – 3.96 (m, 2H), 3.43 (td, *J* = 8.2, 1.5 Hz, 1H), 2.19 (d, *J* = 8.6 Hz, 1H), 1.52 (s, 3H), 1.41 – 1.38 (m, 6H), 1.36 – 1.33 (m, 3H).

^{13}C NMR (101 MHz, Chloroform-*d*) δ 129.8, 123.6, 109.6, 109.1, 76.74, 76.69, 76.3, 70.6, 67.3, 26.9, 26.8, 25.4, 24.6.

F.2.21. (2*R*,3*R*,4*S*)-2,3,4-Tris(benzyloxy)non-5-en-1-ol (*xylo*) (2u)

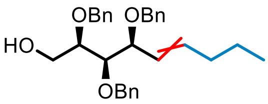

2u
E:Z (1:2)

Obtained 61 mg (66%) as colourless oil as E/Z mixture > 1:2 from **1f** following slightly modified general procedure B2, using LiHMDS.

HRMS calcd. for $[C_{30}H_{36}O_4]Na$: 483.2506, found 483.2516.

^{1}H NMR (400 MHz, Chloroform-*d*) δ 7.39 – 7.27 (m, 24H), 5.74 – 5.64 (m, 1.6H), 5.55 (ddt, *J* = 11.0, 9.4, 1.6 Hz, 1H, *Z*), 5.48 (ddt, *J* = 15.5, 8.1, 1.3 Hz, 0.6H, *E*), 4.78 – 4.72 (m, 3.2H), 4.67 – 4.59 (m, 4.8H), 4.50 – 4.44 (m, 1H, *Z*), 4.36 (dd, *J* = 11.8, 6.1 Hz, 1.6H), 4.07 (dd, *J* = 8.1, 4.9 Hz, 0.6H, *E*), 3.78 – 3.61 (m, 4.8H), 3.61 – 3.53 (m, 1.6H), 2.32 – 2.25 (m, 1H, *Z*), 2.22 – 2.16 (m, 0.6H, *E*), 2.12 – 1.92 (m, 3.2H), 1.44 – 1.33 (m, 3.2H), 0.93 (t, *J* = 7.4 Hz, 1.8H, *E*), 0.88 (t, *J* = 7.4 Hz, 3H, *Z*). **E*/*Z* isomer mixture in ratio 0.6:1

^{13}C NMR (101 MHz, Chloroform-*d*) δ 138.7, 138.6, 138.53, 138.48, 138.4, 138.3, 136.1, 135.4, 128.6, 128.54, 128.49, 128.47, 128.46, 128.45, 128.4, 128.19, 128.15, 128.0, 127.9, 127.83, 127.77, 127.74, 127.71, 127.2, 127.0, 82.3, 82.1, 80.3, 80.0, 79.7, 75.1, 74.9, 74.1, 73.0, 72.9, 70.33, 70.30, 61.7, 61.7, 34.6, 30.1, 22.9, 22.4, 14.0, 13.9. **E*/*Z* isomer mixture

F.2.22. (2*R*,3*R*,4*S*)-2,3,4-Tris(benzyloxy)-6-cyclopropylhex-5-en-1-ol (*xylo*) (2v)

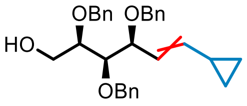

2v
E:Z (1:3)

Obtained 43 mg (47%) as colourless oil as E/Z mixture > 1:3 from **1f** following slightly modified general procedure B2, using LiHMDS.

HRMS calcd. for $[C_{30}H_{34}O_4]Na$: 481.2349, found 483.2358.

^{1}H NMR (600 MHz, Chloroform-*d*) δ 7.55 – 7.07 (m, 21H), 5.48 (dd, *J* = 15.4, 8.1 Hz, 0.4H), 5.37 (ddd, *J* = 10.6, 9.4, 0.8 Hz, 1H), 5.16 (dd, *J* = 15.4, 8.8 Hz, 0.4H), 5.06 – 4.97 (m, 1H), 4.84 – 4.82 (m, 0.4H), 4.78 (d, *J* = 11.4 Hz, 1H), 4.75 – 4.71 (m, 2H), 4.67 (d, *J* = 11.8 Hz, 1.2H), 4.63 – 4.55 (m, 4.4H), 4.41 (d, *J* = 11.8 Hz, 1H), 4.32 (d, *J* = 11.8 Hz, 0.4H), 4.03 – 3.98 (m, 0.4H), 3.72 – 3.64 (m, 4H), 3.58 (t, *J* = 5.2 Hz, 0.4H), 3.56 – 3.48 (m, 1.4H), 1.51 – 1.46 (m, 1H), 1.36 (tq, *J* = 8.6, 4.4 Hz, 0.4H), 0.72 – 0.57 (m, 2.8H), 0.40 – 0.25 (m, 2.8H). **E*/*Z* isomer mixture in ratio 0.4:1

^{13}C NMR (151 MHz, Chloroform-d) δ 139.8, 139.7, 138.63, 138.55, 138.48, 138.41, 138.30, 138.29, 128.55, 128.53, 128.49, 128.47, 128.45, 128.44, 128.40, 128.39, 128.35, 128.33, 128.31, 128.2, 128.10, 128.06, 128.04, 128.00, 127.98, 127.83, 127.82, 127.78, 127.76, 127.71, 127.68, 127.64, 127.61, 127.58, 127.56, 124.7, 124.1, 83.2, 82.1, 82.0, 80.5, 80.2, 79.8, 79.53, 79.46, 75.0, 74.9, 74.8, 72.81, 72.76, 70.2, 70.1, 61.7, 61.6, 31.0, 30.3, 7.40, 7.37, 7.0, 6.9. **E*/*Z* isomer mixture

F.3. Utilization Products

F.3.1. 1,2-Dideoxy-3,4-*O*-(1-methylethylidene)-5-*O*-tosyl-L-*erythro*-pent-1-enitol (3a)

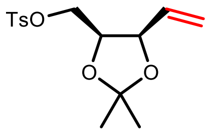

3a

Obtained 57 mg (92%) as colourless oil. The synthesis of **3a** is described in Chapter C.9.

HRMS calcd. for [$C_{15}H_{20}O_5S$]Na: 335.0923, found 335.0928.

^{1}H NMR (400 MHz, Chloroform-*d*) δ 7.82 – 7.75 (m, 2H), 7.34 (d, *J* = 8.0 Hz, 2H), 5.70 (ddd, *J* = 17.2, 10.4, 7.0 Hz, 1H), 5.37 (dt, *J* = 17.1, 1.3 Hz, 1H), 5.22 (dt, *J* = 10.4, 1.3 Hz, 1H), 4.68 – 4.59 (m, 1H), 4.32 (td, *J* = 6.5, 5.2 Hz, 1H), 4.00 (dd, *J* = 10.2, 5.1 Hz, 1H), 3.89 (dd, *J* = 10.2, 6.6 Hz, 1H), 2.44 (s, 3H), 1.38 (s, 3H), 1.33 (s, 3H).

^{13}C NMR (101 MHz, Chloroform-*d*) δ 145.1, 132.9, 131.8, 130.0, 128.2, 119.5, 109.6, 78.0, 75.3, 68.6, 27.7, 25.4, 21.8.

Spectral data in accordance with literature.[8]

F.3.2. 2-{[(4*S*,5*R*)-2,2-Dimethyl-5-vinyl-1,3-dioxolan-4-yl]methyl}isoindoline-1,3-dione (3b)

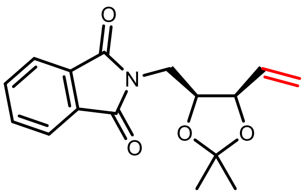

3b

Obtained 49 mg (86%) as colourless solid (m.p.: 82.7–84.9 °C). The synthesis of **3b** is described in Chapter C.10.

HRMS calcd. for [$C_{16}H_{17}NO_5$]Na: 310.1050, found 310.1046.

^{1}H NMR (400 MHz, Chloroform-*d*) δ 7.87 – 7.80 (m, 2H), 7.74 – 7.66 (m, 2H), 5.93 (ddd, *J* = 17.3, 10.4, 6.9 Hz, 1H), 5.51 (d, *J* = 17.2 Hz, 1H), 5.35 (d, *J* = 10.5 Hz, 1H), 4.72 (t, *J* = 6.6 Hz, 1H), 4.60 (ddd, *J* = 9.9, 6.3, 3.1 Hz, 1H), 3.87 (dd, *J* = 13.8, 10.5 Hz, 1H), 3.47 (dd, *J* = 13.8, 3.1 Hz, 1H), 1.56 (s, 3H), 1.32 (s, 3H).

^{13}C NMR (101 MHz, Chloroform-*d*) δ 168.4, 134.0, 132.3, 132.0, 123.4, 119.5, 109.6, 78.2, 74.7, 39.8, 28.0, 25.9.

Spectral data in accordance with literature.[9]

F.3.3. 1,2,5-Trideoxy-3,4-*O*-(1-methylethylidene)-5-iodine-L-*erythro*-pent-1-enitol (3c)

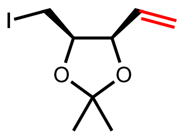

3c

Obtained 46 mg (85%) as colourless oil. The synthesis of **3c** is described in Chapter C.11.

With the available HRMS equipment, no HRMS data for compound **3c** could be obtained.

^{1}H NMR (400 MHz, Chloroform-*d*) δ 5.85 (ddd, *J* = 17.4, 10.4, 7.2 Hz, 1H), 5.43 (dt, *J* = 17.2, 1.3 Hz, 1H), 5.34 (dt, *J* = 10.4, 1.2 Hz, 1H), 4.67 – 4.60 (m, 1H), 4.44 (dt, *J* = 7.6, 6.1 Hz, 1H), 3.15 (dd, *J* = 10.2, 7.6 Hz, 1H), 3.06 (dd, *J* = 10.2, 6.2 Hz, 1H), 1.52 (s, 3H), 1.39 (d, *J* = 0.4 Hz, 3H).

^{13}C NMR (101 MHz, Chloroform-*d*) δ 132.5, 119.6, 109.2, 79.3, 78.7, 28.3, 25.7, 4.0.

Spectral data in accordance with literature.[8]

F.4. Diene-Products

F.4.1. 1,2,4-Trideoxy-3,5,7-tri-*O*-benzyl-D-*gluco*-hept-1,3-dienitol (2b')

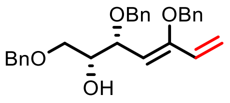

2b'

Obtained 63 mg (73%) or 78 mg (91%) as colorless oil, respectively from **1b** or **1c**, following slightly modified general procedure B2, using KO^{t}Bu (2.5 equiv.) instead of NaHMDS.

^{1}H NMR (400 MHz, Chloroform-*d*) δ 7.34 – 7.18 (m, 15H), 6.20 (dd, *J* = 17.3, 10.8 Hz, 1H), 5.52 (d, *J* = 17.4 Hz, 1H), 5.24 – 5.19 (m, 1H), 5.11 (d, *J* = 9.6 Hz, 1H), 4.77 – 4.67 (m, 1H), 4.48 – 4.42 (m, 3H), 4.41 – 4.36 (m, 1H), 4.23 (d, *J* = 11.9 Hz, 1H), 3.88 – 3.80 (m, 1H), 3.54 – 3.43 (m, 1H), 2.33 – 2.28 (m, 1H).

^{13}C NMR (101 MHz, Chloroform-*d*) δ 157.3, 138.6, 138.2, 137.4, 132.1, 128.6, 128.5, 128.4, 128.1, 128.0, 127.9, 127.8, 127.6, 116.3, 115.4, 73.81, 73.77, 73.5, 72.6, 71.1, 70.4.

F.4.2. 1,2,4-Trideoxy-3,5-di-*O*-benzyl-D-*xylo*-hex-1,3-dienitol (2f')

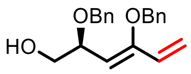

2f'

Obtained 52 mg (84%) as colorless oil from **1f** following slightly modified general procedure B2, using KO^{t}Bu (2.5 equiv.) instead of NaHMDS.

^{1}H NMR (400 MHz, Chloroform-*d*) δ 7.33 – 7.16 (m, 10H), 6.13 (dd, *J* = 17.4, 10.9 Hz, 1H), 5.49 (dd, *J* = 17.4, 1.1 Hz, 1H), 5.23 – 5.17 (m, 1H), 4.94 (d, *J* = 9.3 Hz, 1H), 4.75 (d, *J* = 11.5 Hz, 1H), 4.68 (d, *J* = 11.5 Hz, 1H), 4.44 (d, *J* = 11.7 Hz, 1H), 4.31 (ddd, *J* = 9.3, 7.4, 4.0 Hz, 1H), 4.21 (d, *J* = 11.7 Hz, 1H), 3.43 – 3.28 (m, 2H), 2.01 (s, 1H).

^{13}C NMR (101 MHz, Chloroform-*d*) δ 156.5, 138.5, 137.1, 131.9, 128.69, 128.66, 128.4, 128.38, 128.35, 128.0, 127.77, 127.75, 127.1, 116.6, 115.6, 74.4, 73.7, 70.6, 65.3.

G. References

1 R. C. Sawant, Y.-H. Lih, S.-A. Yang, C.-H. Yeh, H.-J. Tai, C.-L. Huang, H.-S. Lin, S. S. Badsara and S.-Y. Luo, *RSC Adv.*, 2014, **4**, 26524–26534.

2 I. S. Kim, O. P. Zee and Y. H. Jung, *Org. Lett.*, 2006, **8**, 4101–4104.

3 N. A. Ivanova, Z. R. Valiullina, O. V. Shitikova, L. V. Spirikhin and M. S. Miftakhov, *Russ J Org Chem*, 2008, **44**, 335–339.

4 M. Heinrich, J. J. Murphy, M. K. Ilg, A. Letort, J. T. Flasz, P. Philipps and A. Fürstner, *J. Am. Chem. Soc.*, 2020, **142**, 6409–6422.

5 Z. Zhao, L. Racicot and G. K. Murphy, *Angew Chem Int Ed*, 2017, **56**, 11620–11623.

6 P. Bonilla, Y. P. Rey, C. M. Holden and P. Melchiorre, *Angew Chem Int Ed*, 2018, **57**, 12819–12823.

7 Y. Liu, J. Liu, C. Zhao and Y. Du, *Org. Lett.*, 2021, **23**, 3264–3268.

8 S. G. Davies, E. M. Foster, A. B. Frost, J. A. Lee, P. M. Roberts and J. E. Thomson, *Org. Biomol. Chem.*, 2012, **10**, 6186.

9 A. Shaw, S. Ajay, I. Arora and P. Saidhareddy, *Synlett*, 2016, **27**, 2721–2725.

10 S. D. Markad, S. Xia, N. L. Snyder, B. Surana, M. D. Morton, C. M. Hadad and M. W. Peczuh, *J. Org. Chem.*, 2008, **73**, 6341–6354.

11 G. Godin, E. Garnier, P. Compain, O. R. Martin, K. Ikeda and N. Asano, *Tetrahedron Letters*, 2004, **45**, 579–581.

12 O. N. Nadein and A. Kornienko, *Org. Lett.*, 2004, **6**, 831–834.

13 T. Chan, Y. Chang, J. Hsu and W. Cheng, *Eur J Org Chem*, 2010, **2010**, 5555–5559.

14 R. Fürst, C. Lentsch and U. Rinner, *Eur J Org Chem*, 2013, **2013**, 2293–2297.

15 S. Mandal, D. Mahananda, S. Dey, R. S. Bharathavikru and B. Thirupathi, *J. Nat. Prod.*, 2024, **87**, 152–159.

16 H.-Y. Chiu, D.-L. M. Tzou, L. N. Patkar and C.-C. Lin, *J. Org. Chem.*, 2003, **68**, 5788–5791.

17 V. S. Dorokhova, A. G. Gerbst, B. S. Komarova, J. O. Previato, L. M. Previato, A. S. Dmitrenok, A. S. Shashkov, V. B. Krylov and N. E. Nifantiev, *Org. Biomol. Chem.*, 2021, **19**, 2923–2931.

18 S. Choi, S. O. Mansoorabadi, Y. Liu, T.-C. Chien and H. Liu, *J. Am. Chem. Soc.*, 2012, **134**, 13946–13949.

19 I. S. Kim, O. P. Zee and Y. H. Jung, *Org. Lett.*, 2006, **8**, 4101–4104.

20 B. K. Jena, G. S. Reddy and D. K. Mohapatra, *Org. Biomol. Chem.*, 2017, **15**, 1863–1871.

21 P. Pasetto and M. C. Walczak, *Tetrahedron*, 2009, **65**, 8468–8477.

22 Y. Okuyama, M. Kidena, E. Kato, S. Kawano, K. Ishii, K. Maie, K. Miura, S. Simizu, T. Sato and N. Chida, *Angew Chem Int Ed*, 2021, **60**, 5193–5198.

H. NMR Spectra

H.1. Starting materials

H.1.1. NMR of 2,3,4,6-tetra-*O*-benzyl-D-*galacto*-pyranose (1e)

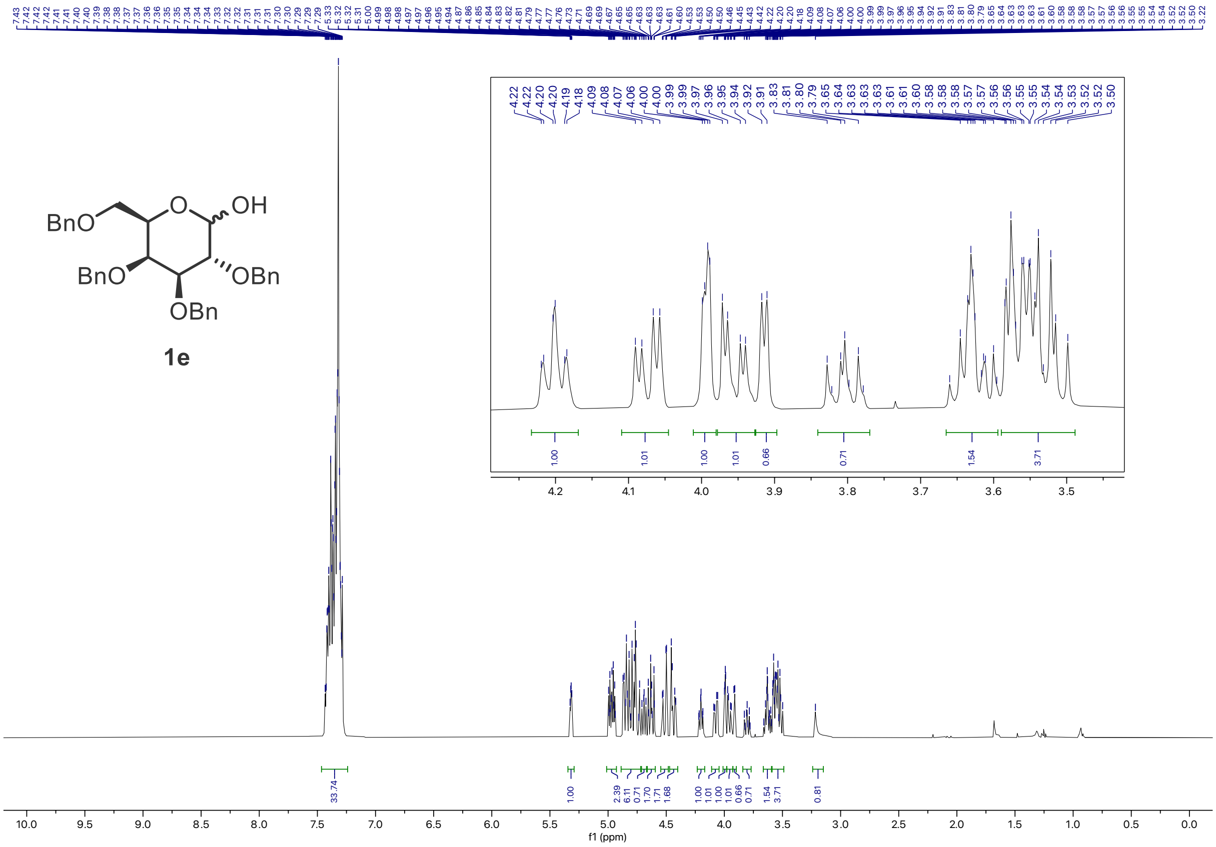

Figure S4. *400 MHz* ^{1}*H NMR of **1e** as anomeric mixture α:β 0.7:1*

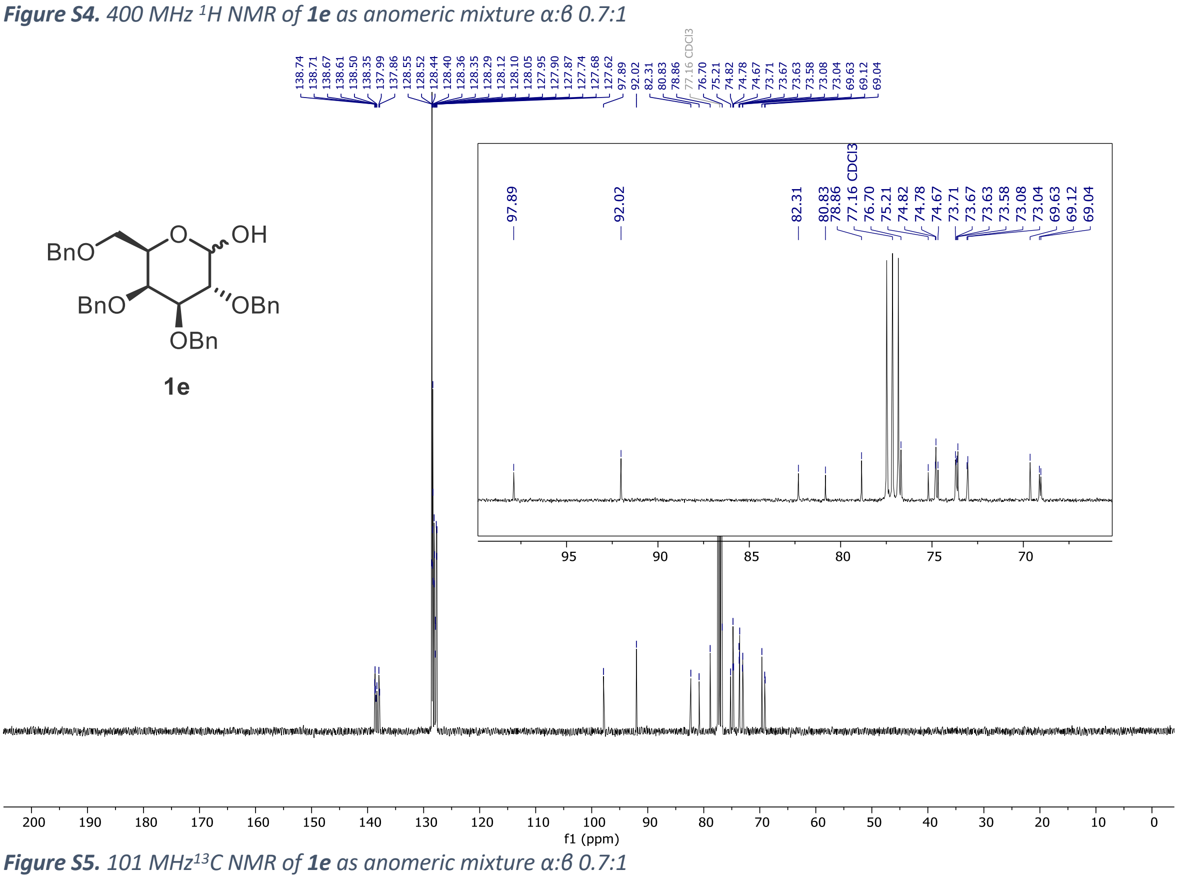

Figure S5. *101 MHz*^{13}*C NMR of **1e** as anomeric mixture α:β 0.7:1*

H.1.2. NMR of 2,3,4-tri-*O*-benzyl-D-*xylo*-pyranose (1f)

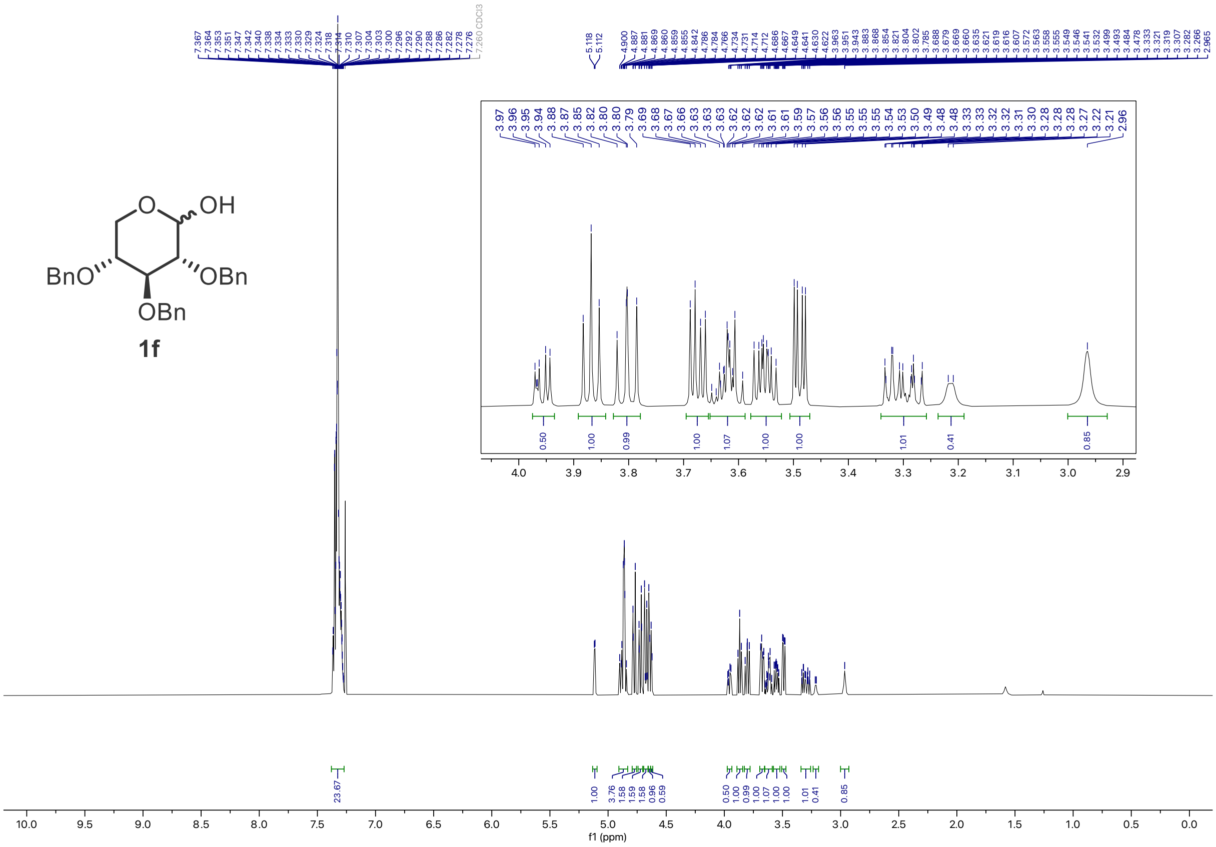

Figure S6. *600 MHz ^{1}H NMR of **1f** as anomeric mixture α:β 2:1*

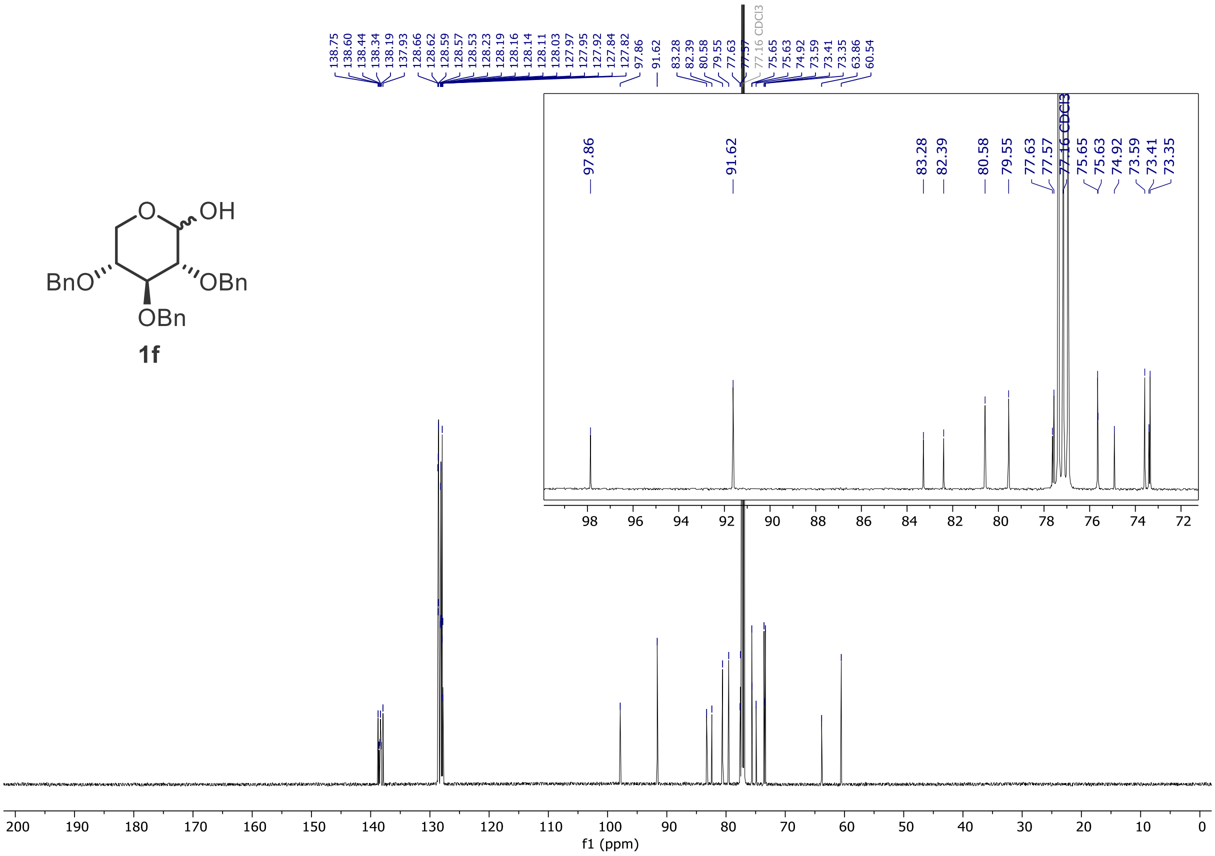

Figure S7. *151 MHz ^{13}C NMR of **1f** as anomeric mixture α:β 2:1*

H.1.3. NMR of 2,3,4-tri-*O*-benzyl-D-*arabino*-pyranose (1g)

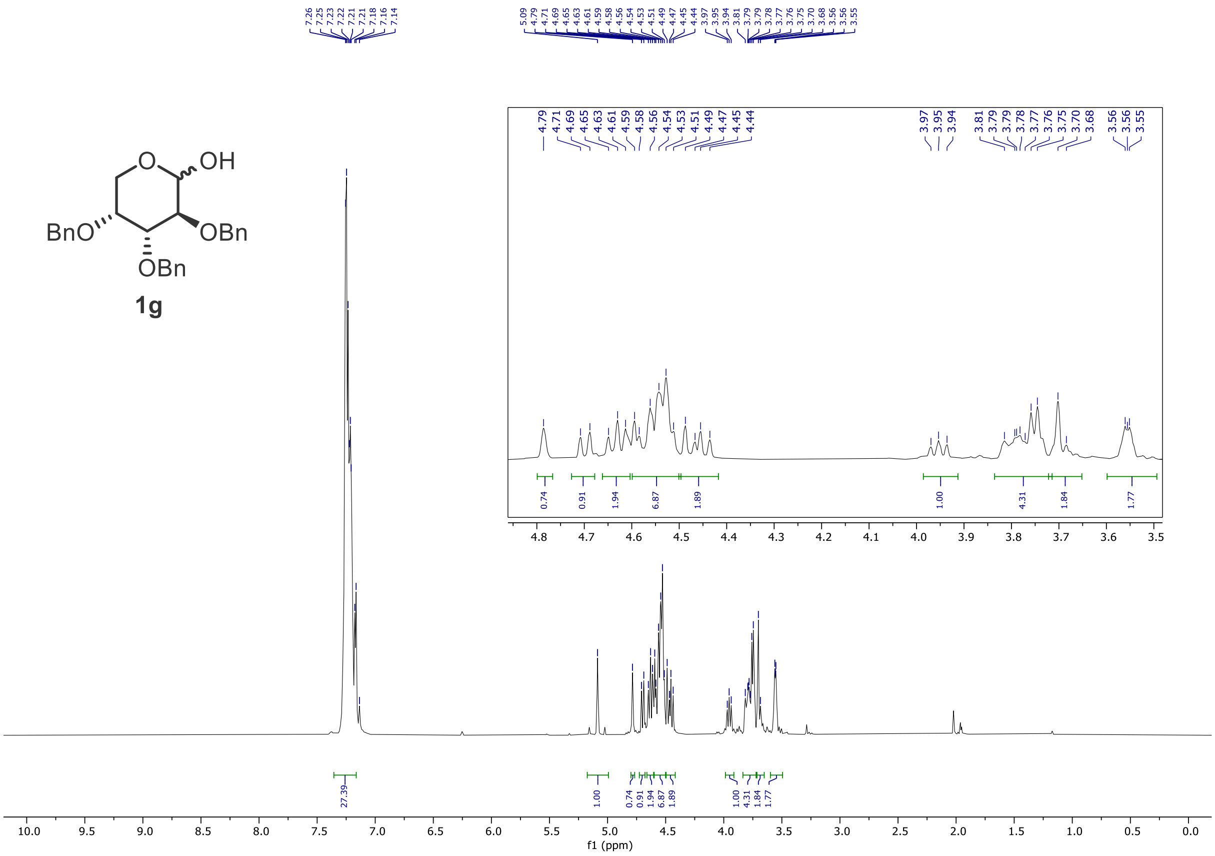

Figure S8. *600 MHz ¹H NMR of* ***1g*** *as anomeric mixture α:β 1:0.8*

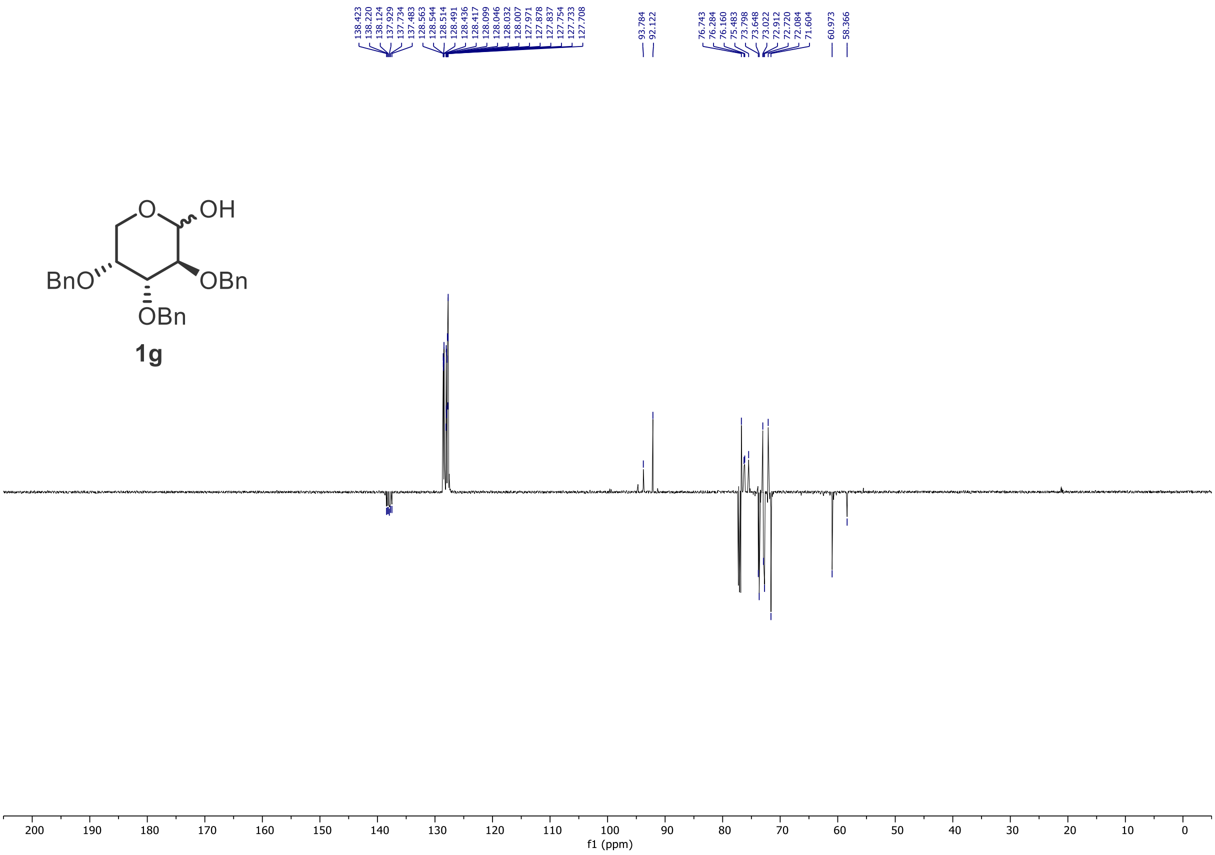

Figure S9. *151 MHz ¹³C NMR of* ***1g*** *as anomeric mixture α:β 1:0.8*

H.1.4. NMR of 2,3-*O*-isopropylidene-D-*ribo*-furanose (1i)

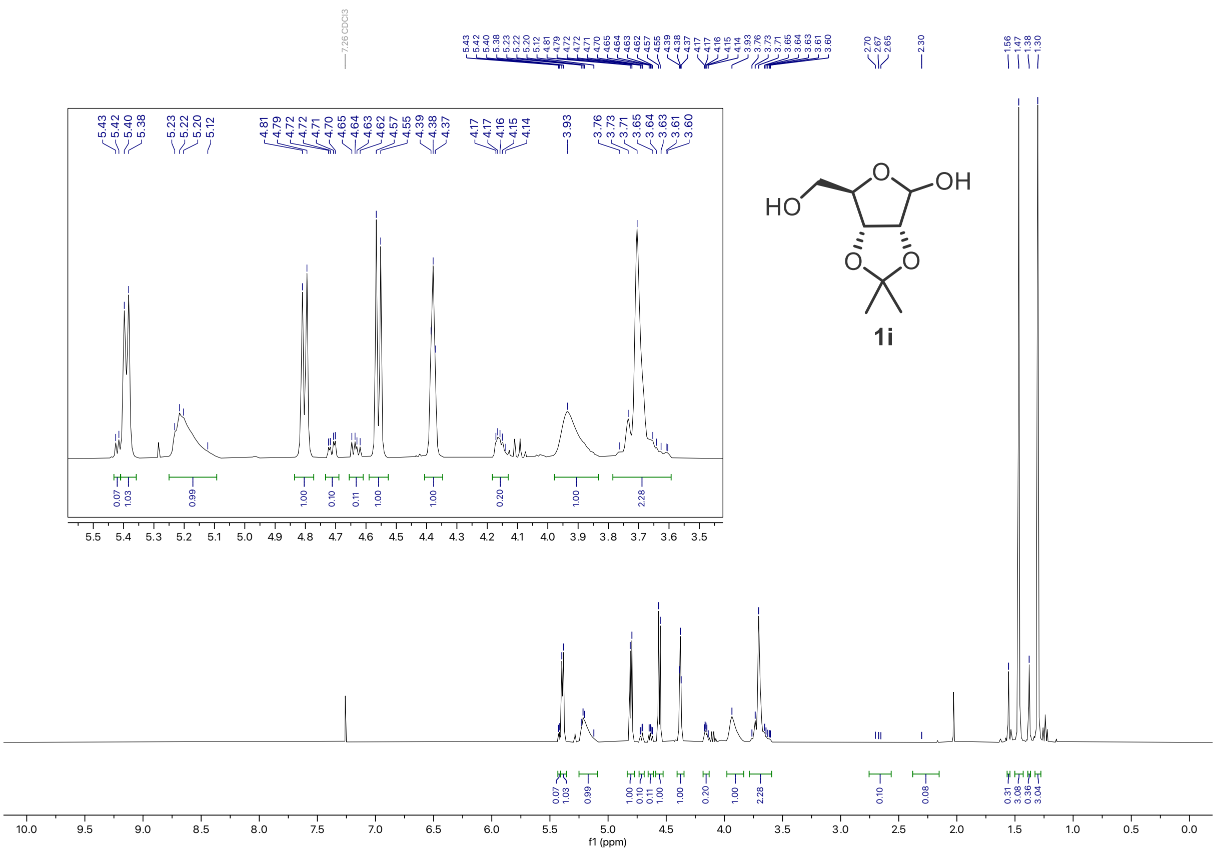

Figure S10. *400 MHz ^{1}H NMR of **1i** as anomeric mixture α:β 0.1:1*

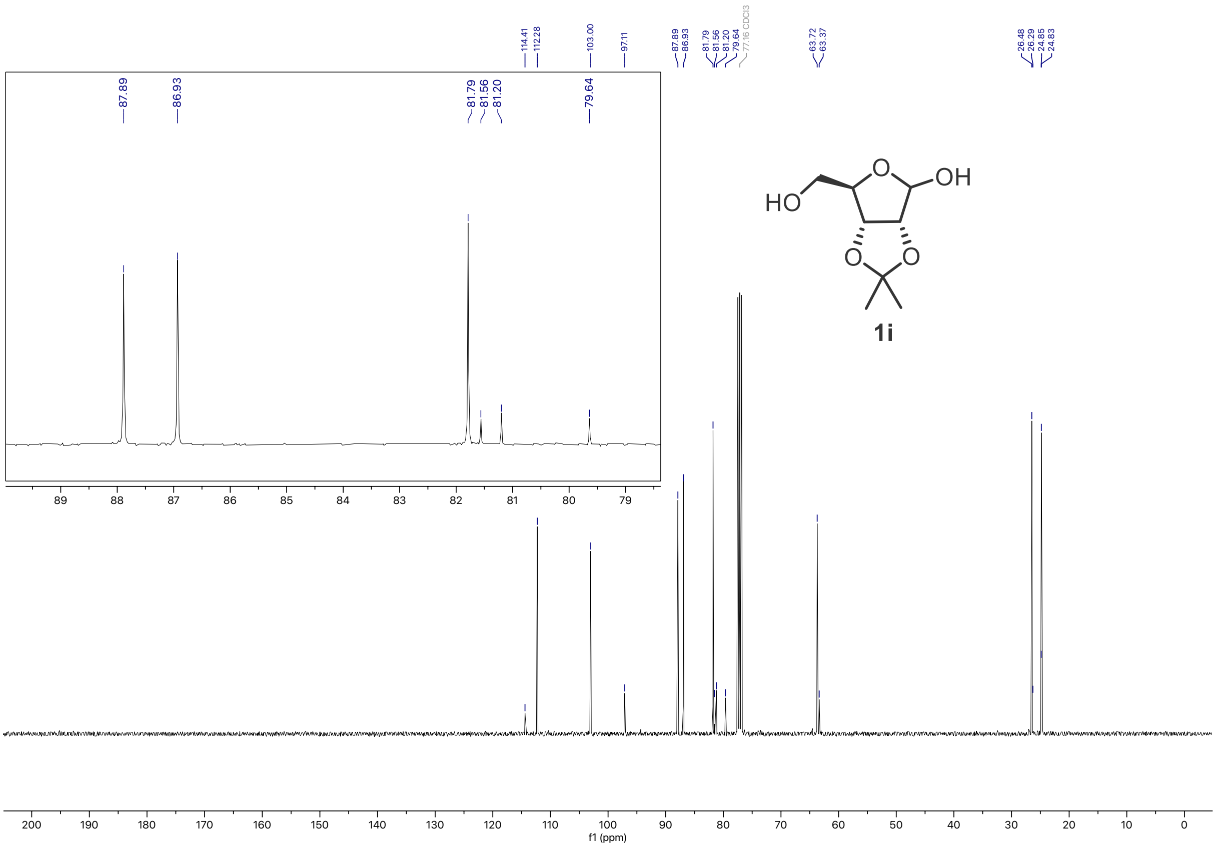

Figure S11. *101 MHz ^{13}C NMR of **1i** as anomeric mixture α:β 0.1:1*

H.1.5. NMR of 3,4-tri-*O*-isopropylidene-L-*erythro*-furanose (1j)

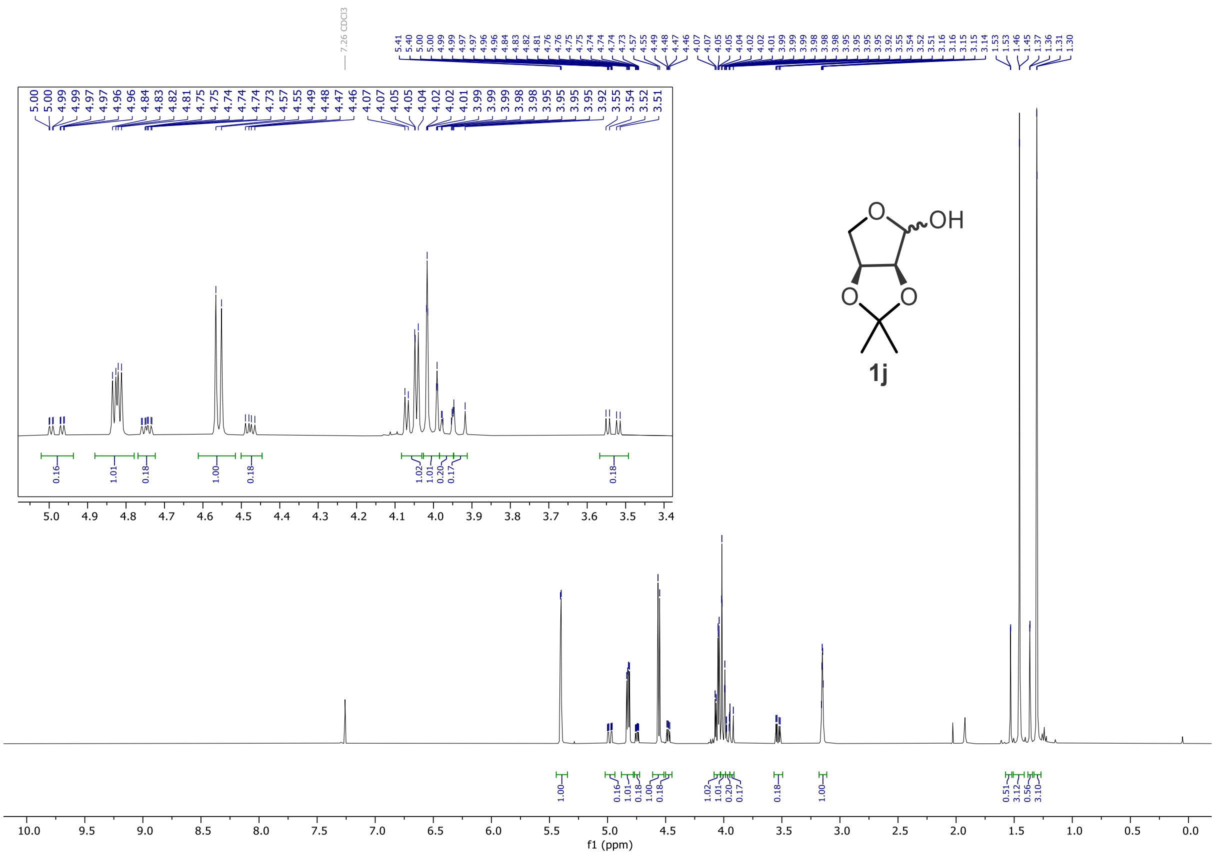

Figure S12. *600 MHz ^{1}H NMR of* ***1j*** *as anomeric mixture α:β 0.15:1*

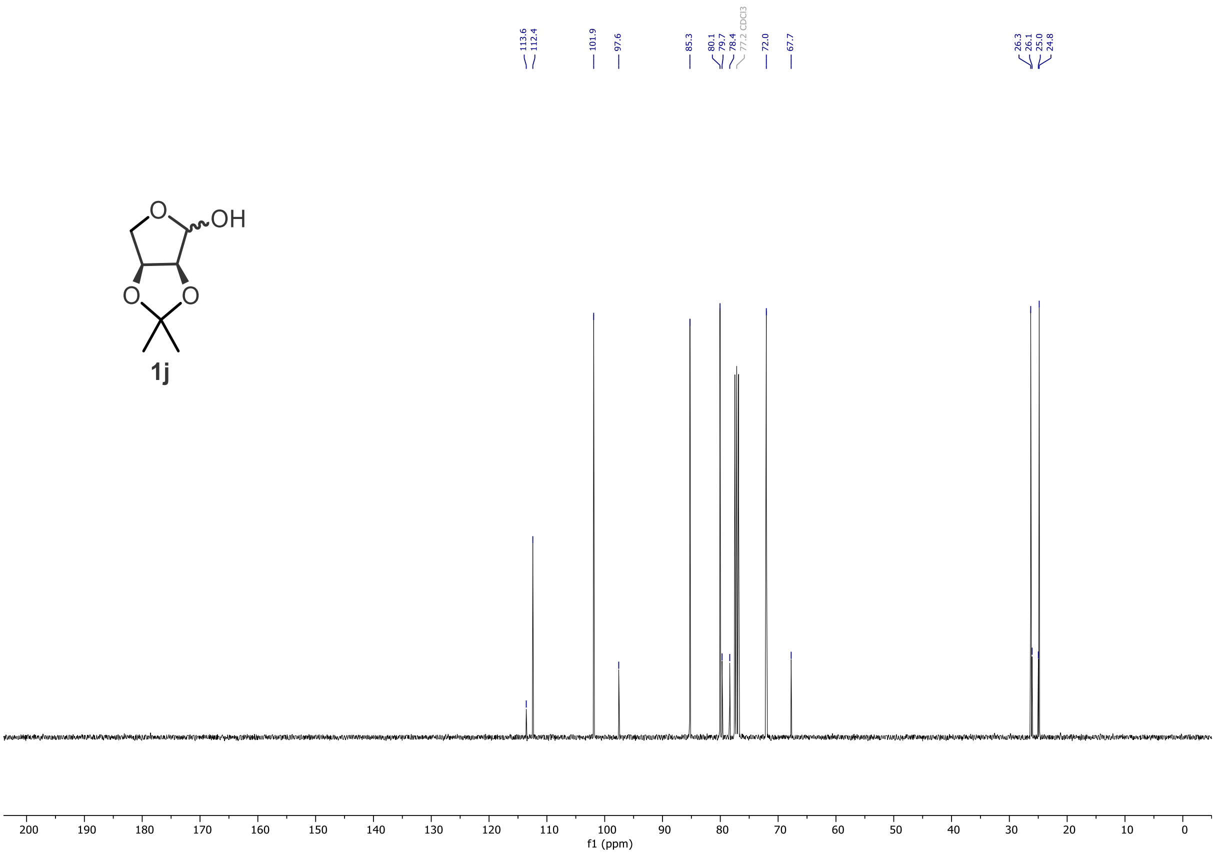

Figure S13. *151 MHz ^{13}C NMR of* ***1j*** *as anomeric mixture α:β 0.15:1*

H.2. Olefination Products

H.2.1. NMR of 1,2-dideoxy-3,4;6,7-di-*O*-isopropylidene-D-*manno*-hept-1-enitol (2a)

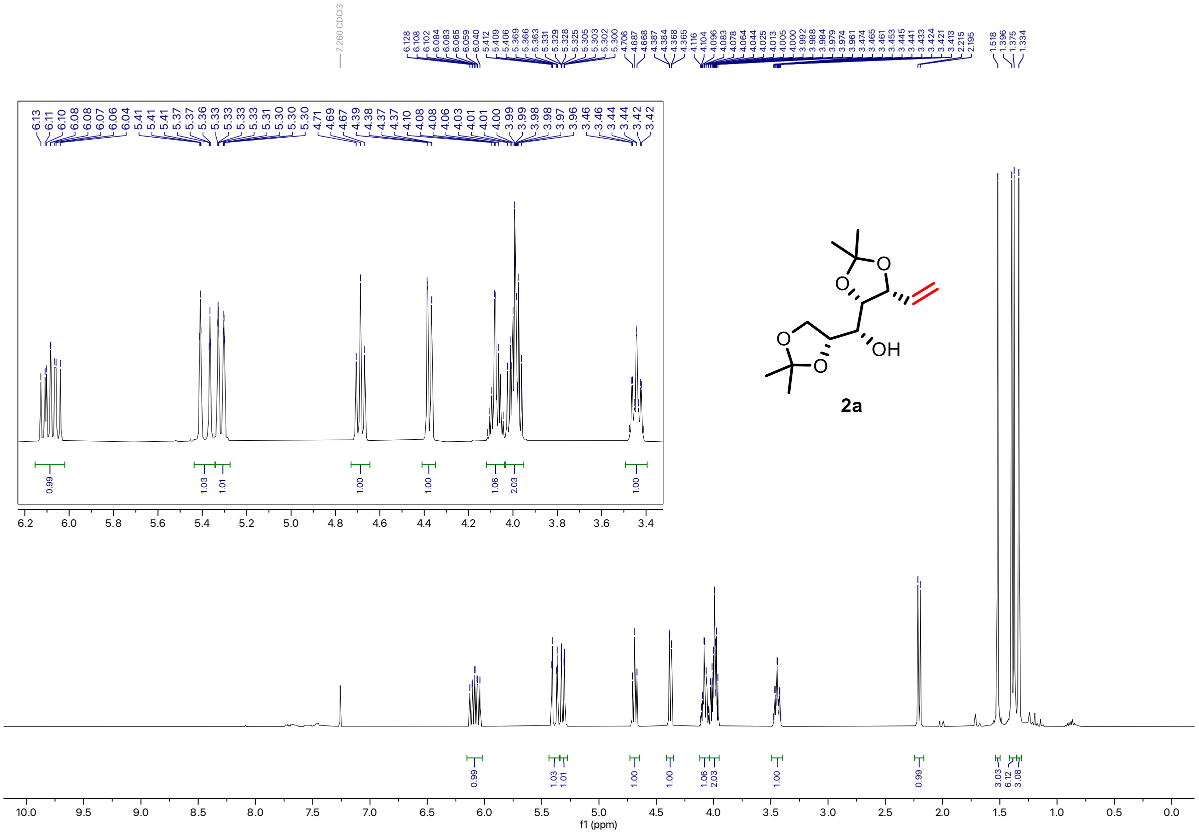

Figure S14. *400 MHz ^{1}H NMR of **2a***

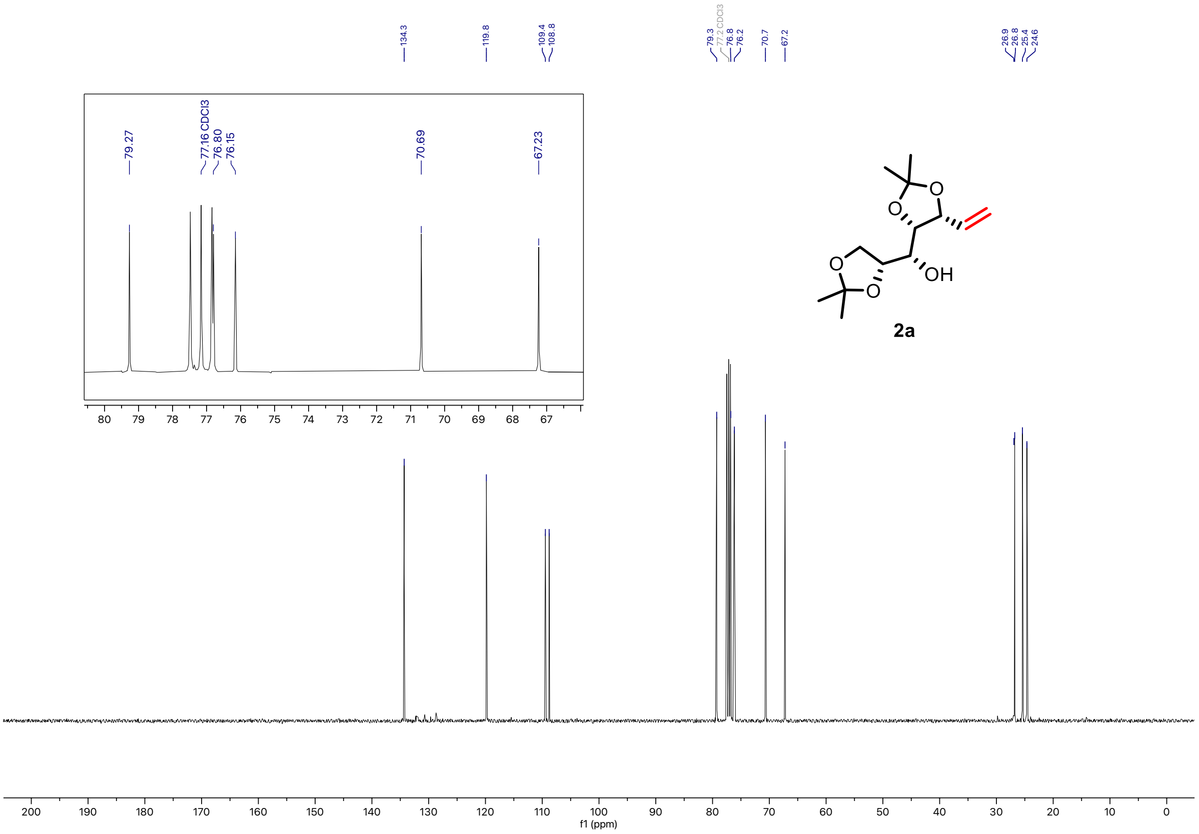

Figure S15. *101 MHz ^{13}C NMR of **2a***

H.2.2. NMR of 1,2-dideoxy-3,4,5,7-tetra-*O*-benzyl-D-*manno*-hept-1-enitol (2b)

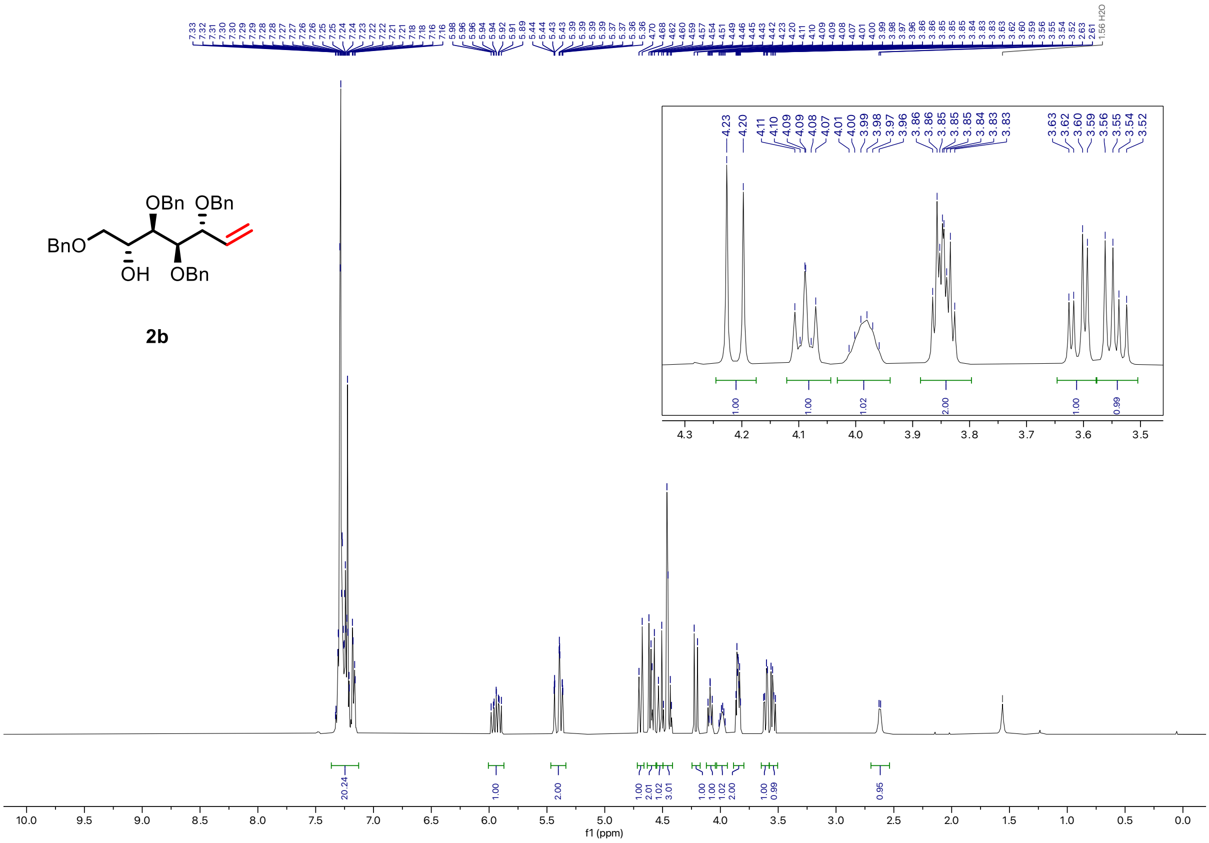

Figure S16. *400 MHz 1H NMR of **2b***

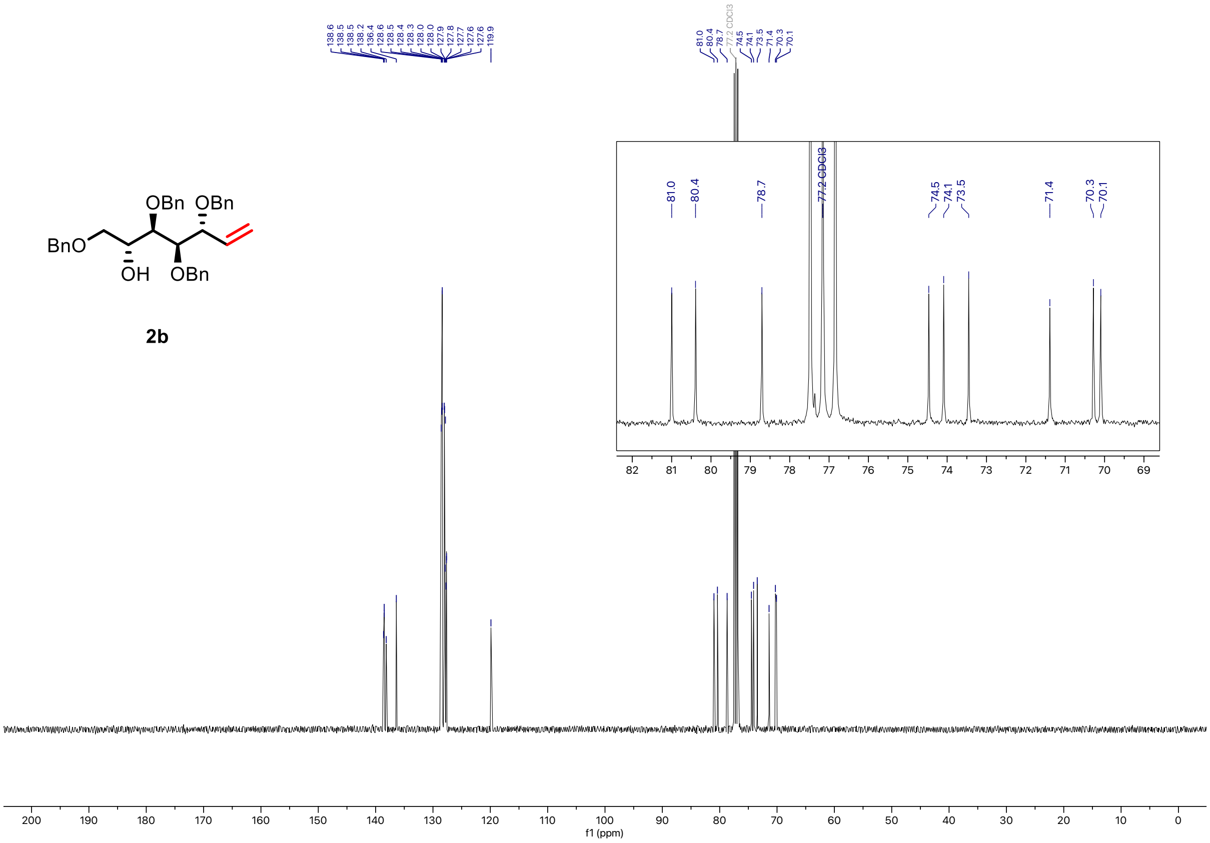

Figure S17. *101 MHz ^{13}C NMR of **2b***

H.2.3. NMR of 1,2-dideoxy-3,4,5,7-tetra-*O*-benzyl-D-*gluco*-hept-1-enitol (2c)

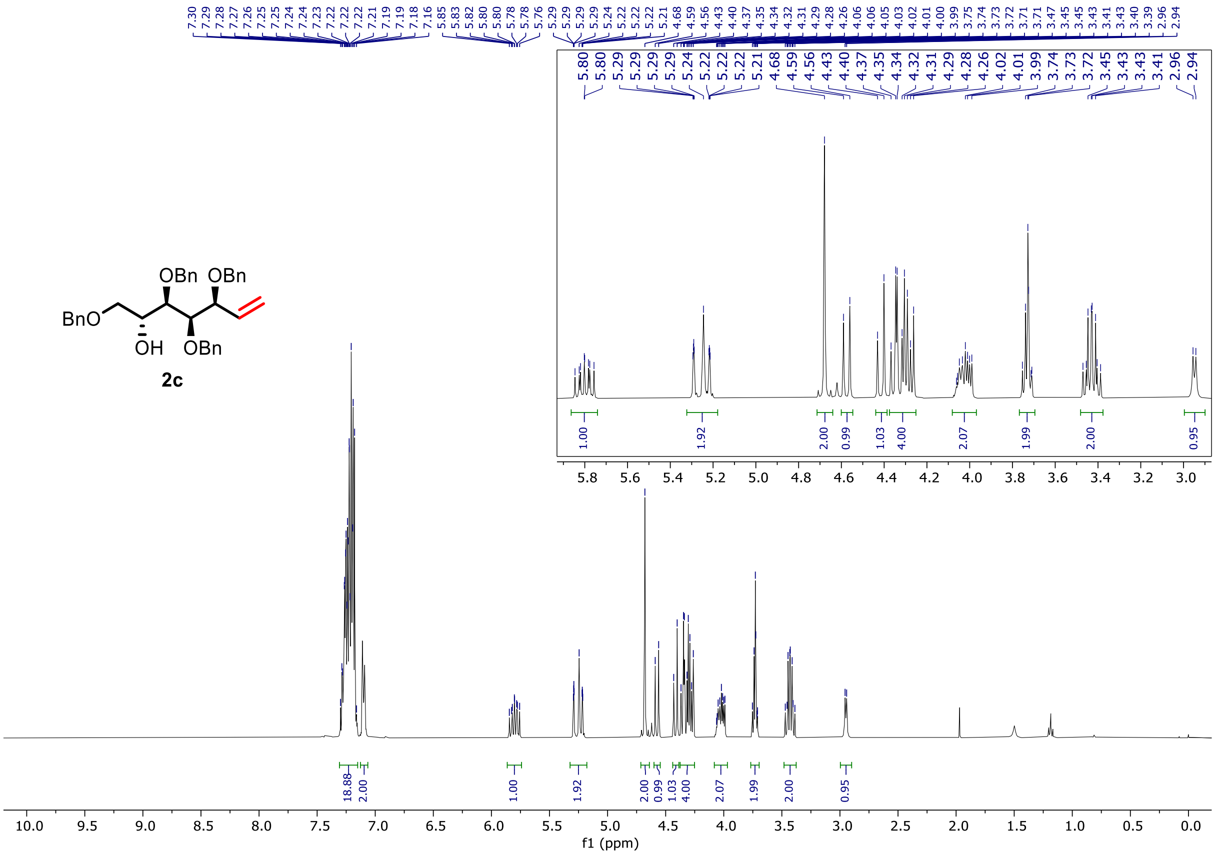

Figure S18. *400 MHz ^{1}H NMR of* ***2c***

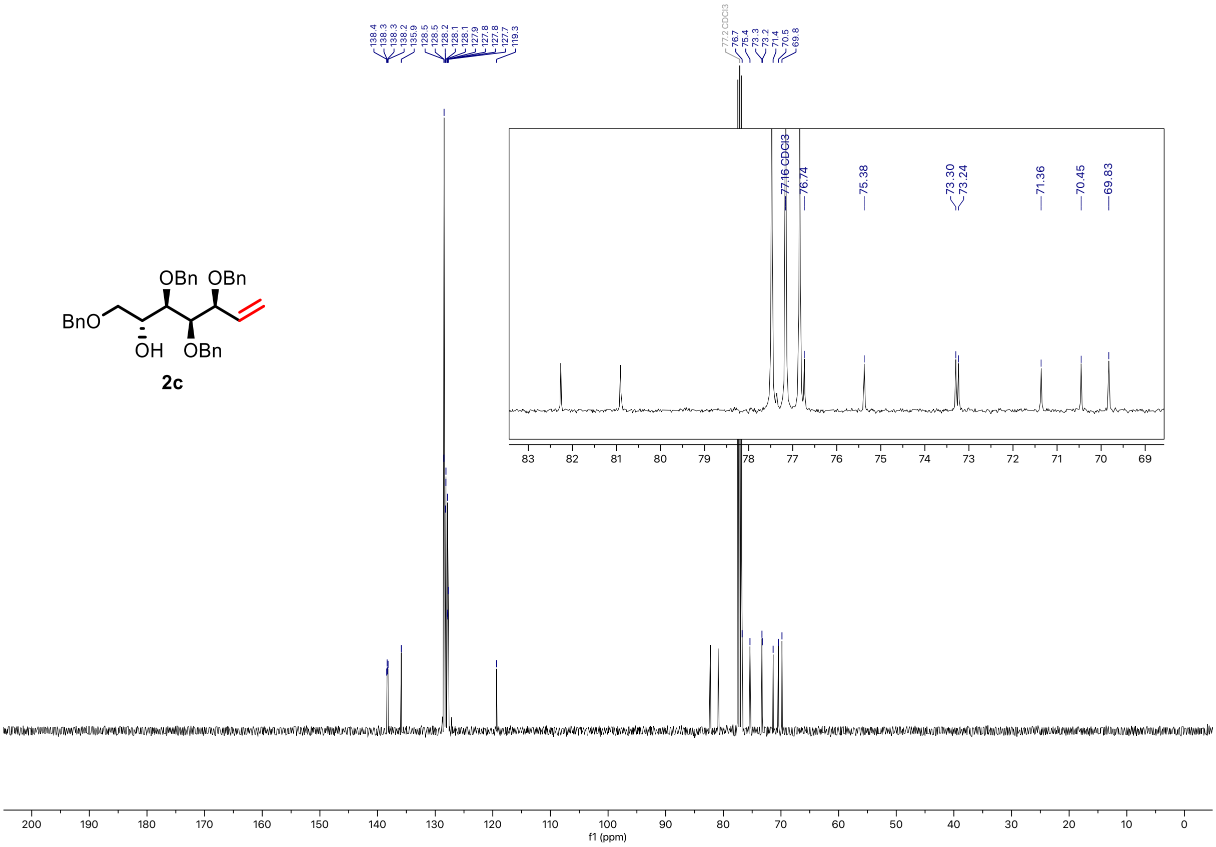

Figure S19. *101 MHz ^{13}C NMR of* ***2c***

H.2.4. NMR of 1,2-dideoxy-5,7-*O*-(phenylmethyl)-d-*gluco*-hept-1-enitol (2d)

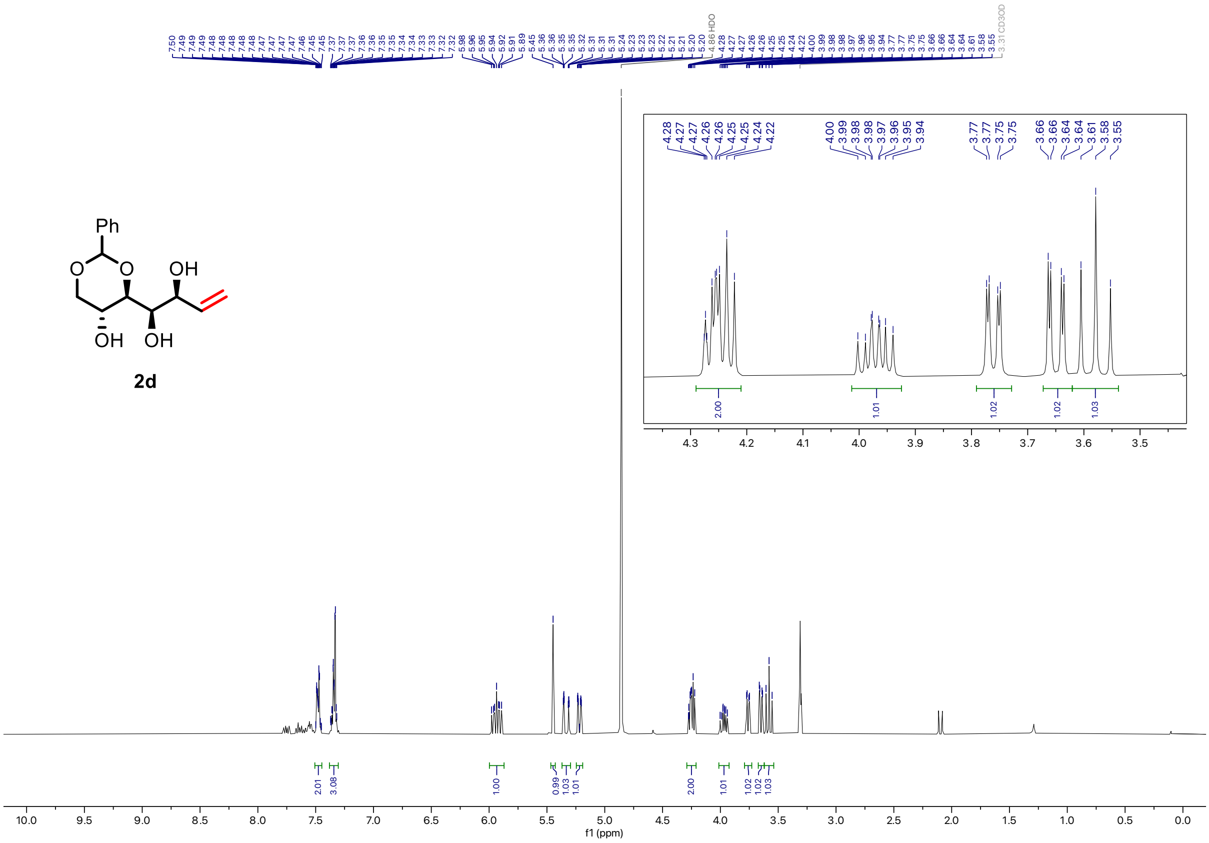

Figure S20. *400 MHz* *^{1}H NMR of* ***2d***

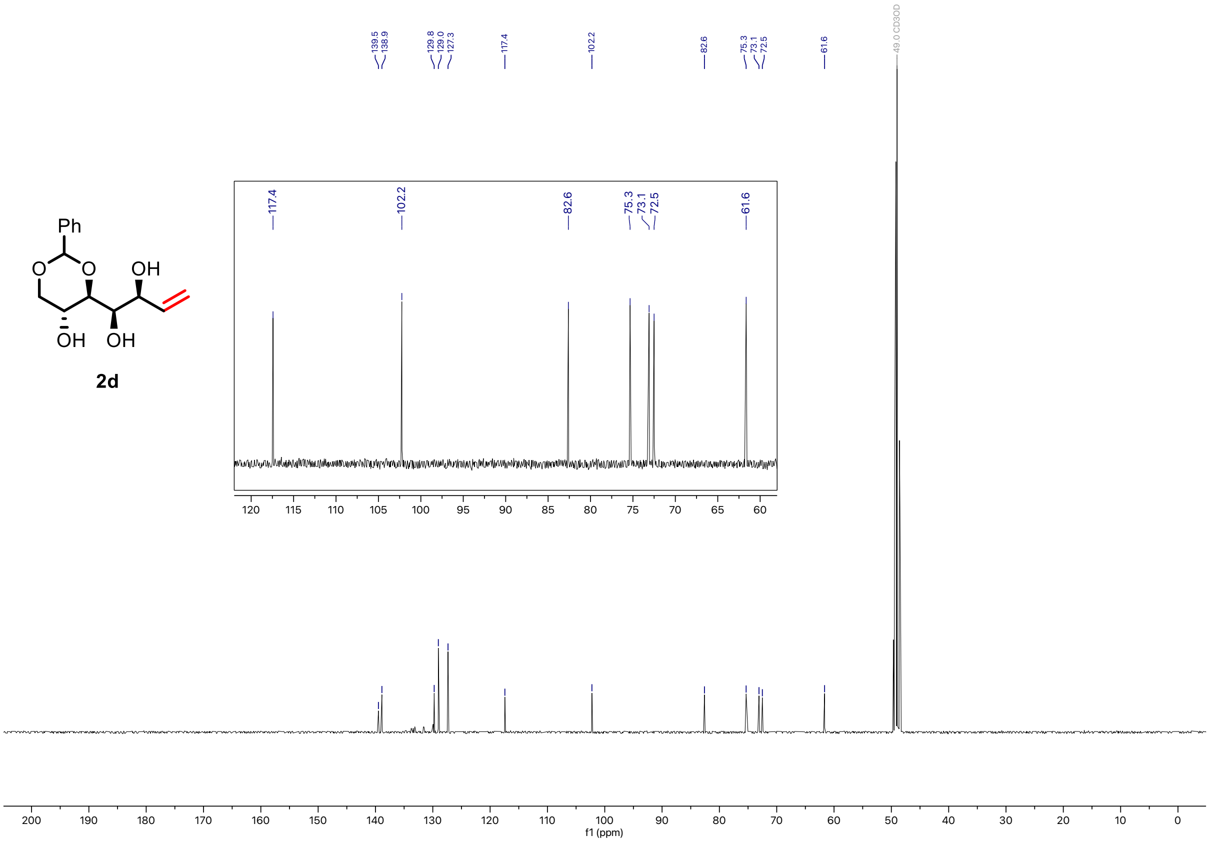

Figure S21. *101 MHz* *^{13}C NMR of* ***2d***

H.2.5. NMR of 1,2-dideoxy-3,4,5,7-tetra-*O*-benzyl-D-*galacto*-hept-1-enitol (2e)

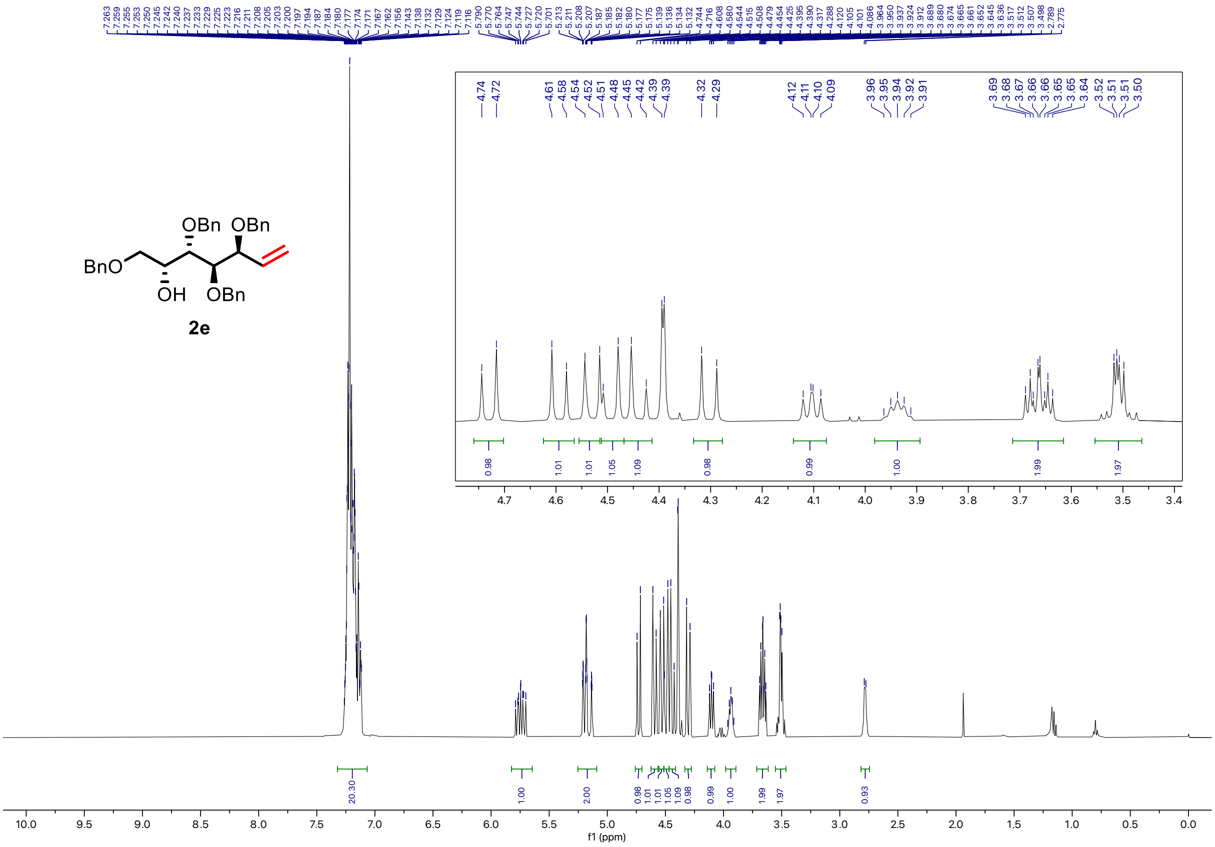

Figure S22. 400 MHz ^{1}H NMR of **2e**

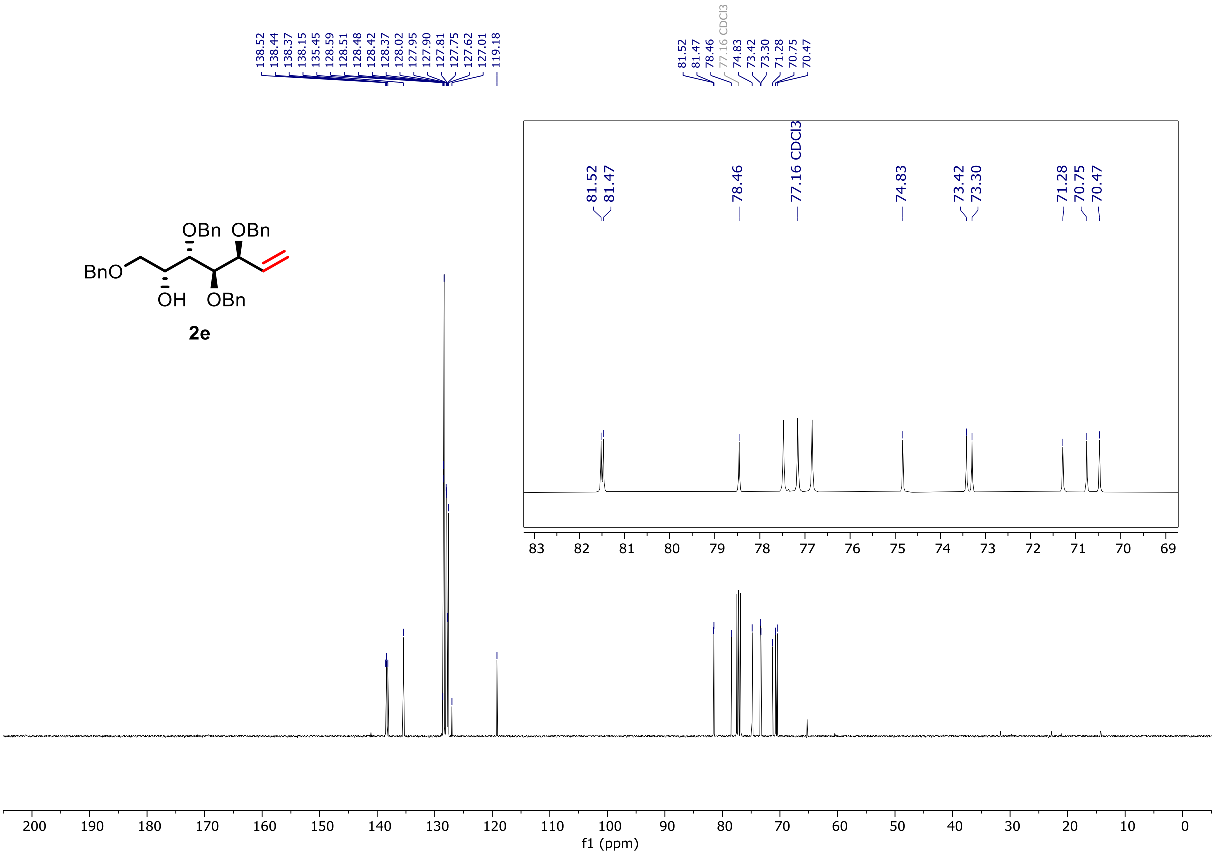

Figure S23. 101 MHz ^{13}C NMR of **2e**

H.2.6. NMR of 1,2-dideoxy-3,4,5-tri-*O*-benzyl-D-*xylo*-hex-1-enitol (2f)

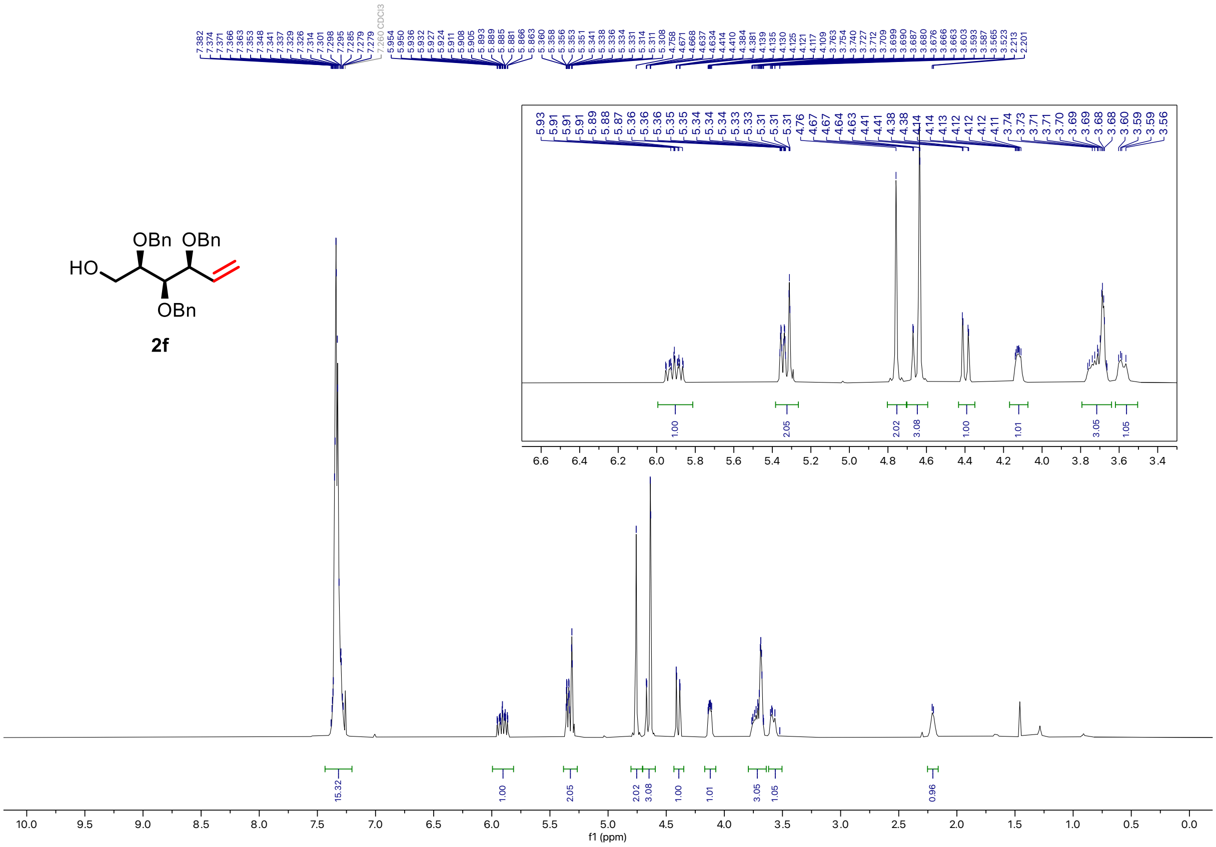

Figure S24. *400 MHz ^{1}H NMR of* ***2f***

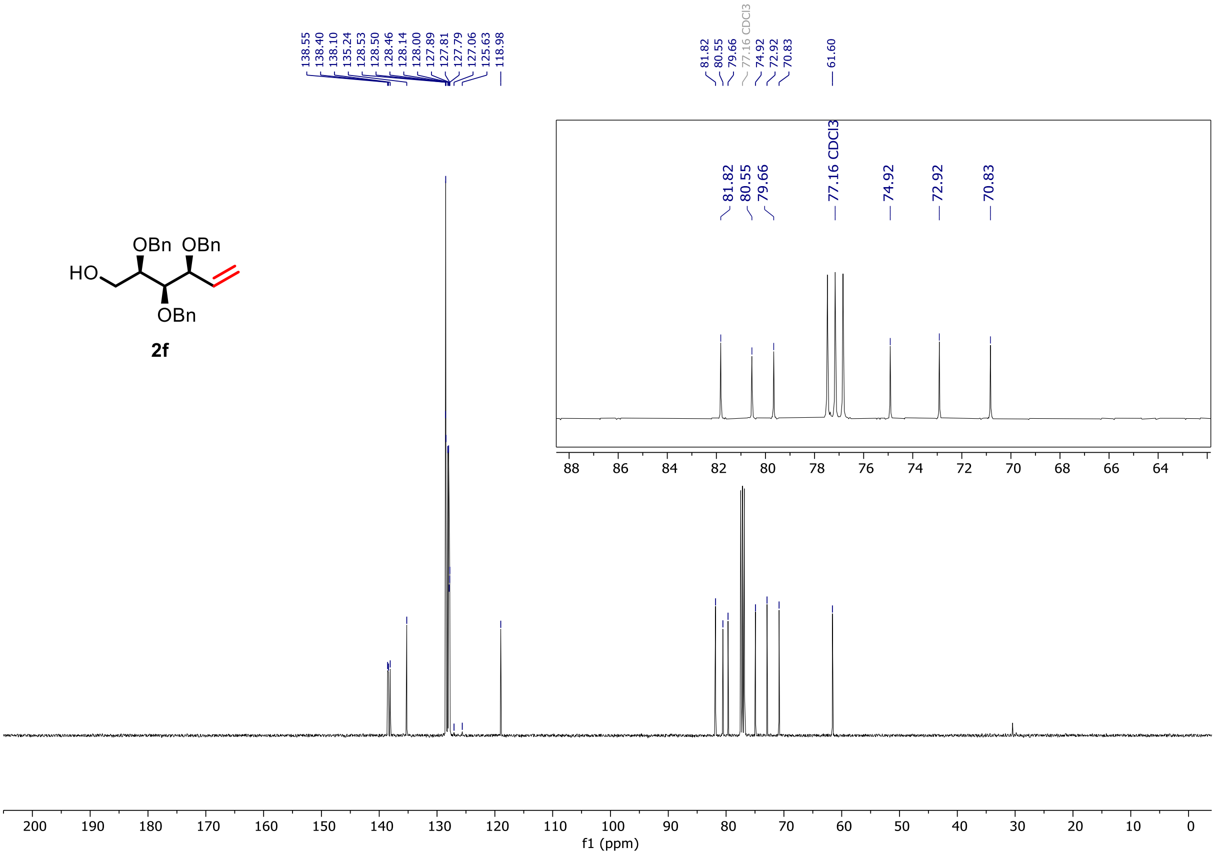

Figure S25. *101 MHz ^{13}C NMR of* ***2f***

H.2.7. NMR of 1,2-dideoxy-3,4,5-tri-*O*-benzyl-D-*arabino*-hex-1-enitol (2g)

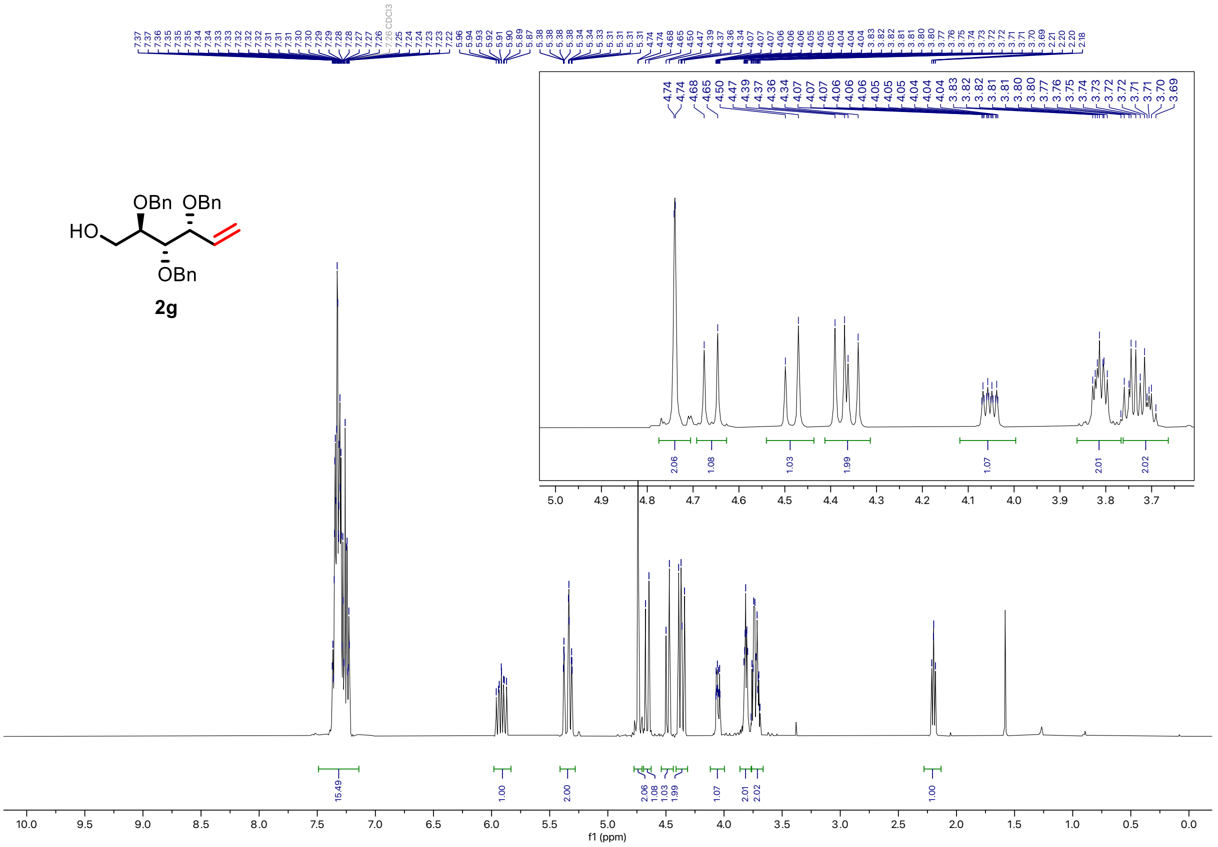

Figure S26. *400 MHz* ^{1}*H NMR of* ***2g***

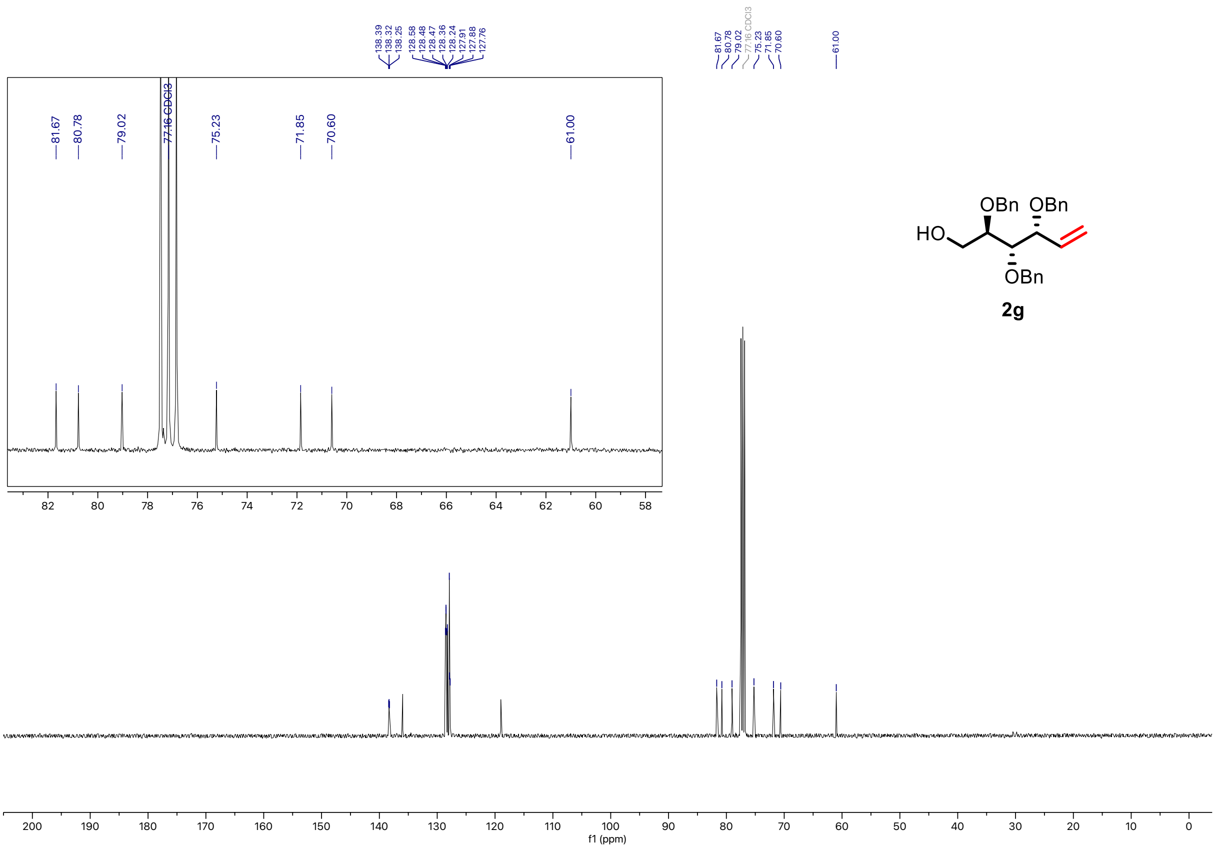

Figure S27. *101 MHz* ^{13}*C NMR of* ***2g***

H.2.8. NMR of 1,2-dideoxy-D-*arabino*-hex-1-enitol (2h)

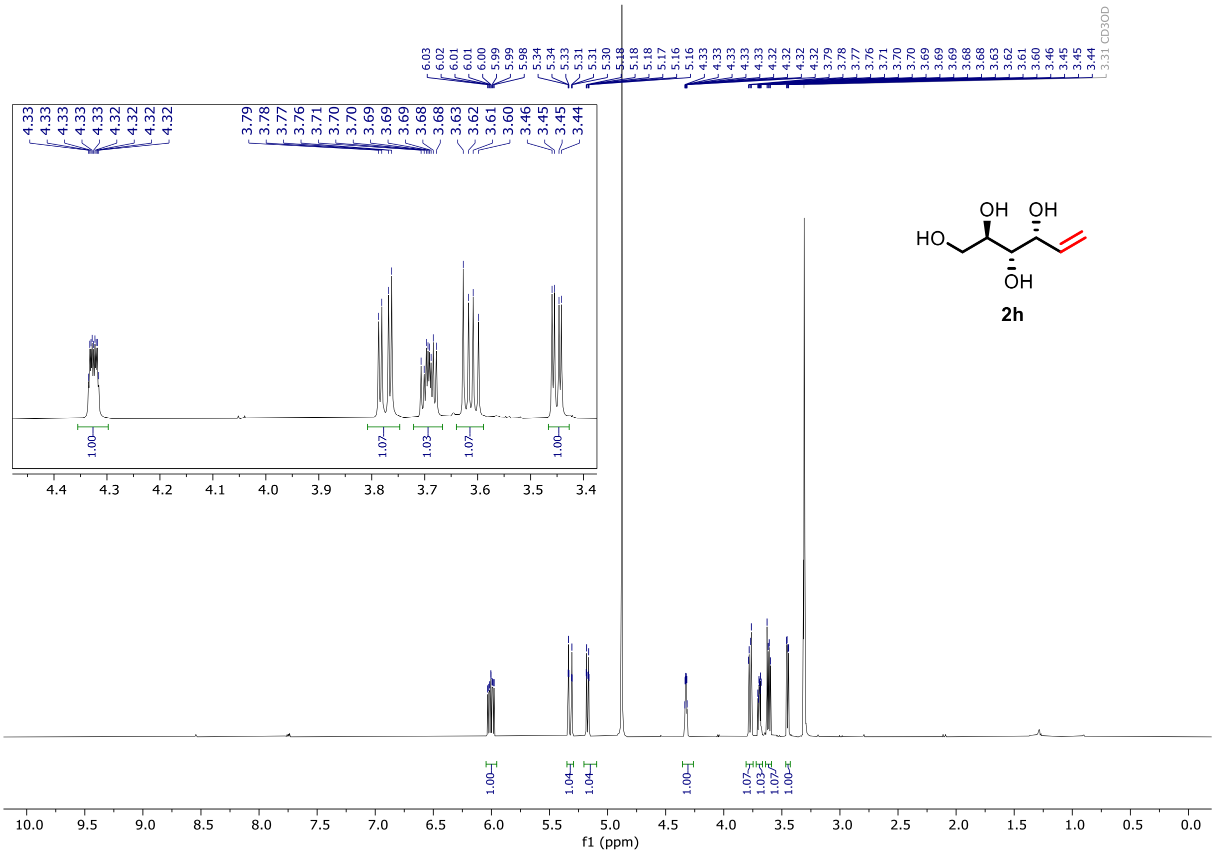

Figure S28. *400 MHz ^{1}H NMR of* ***2h***

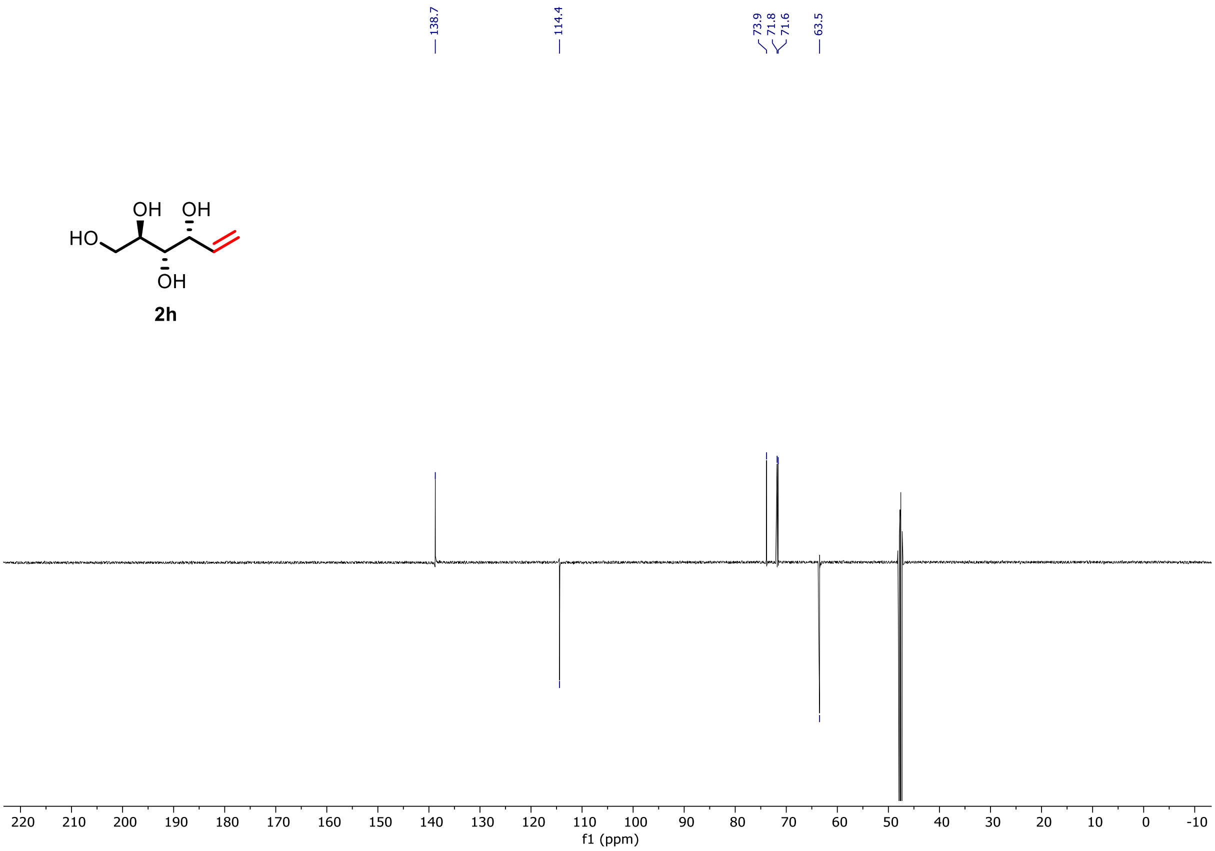

Figure S29. *101 MHz ^{13}C NMR of* ***2h***

H.2.9. NMR of 1,2-dideoxy-3,4-*O*-(1-methylethylidene)-D-*ribo*-hex-1-enitol (2i)

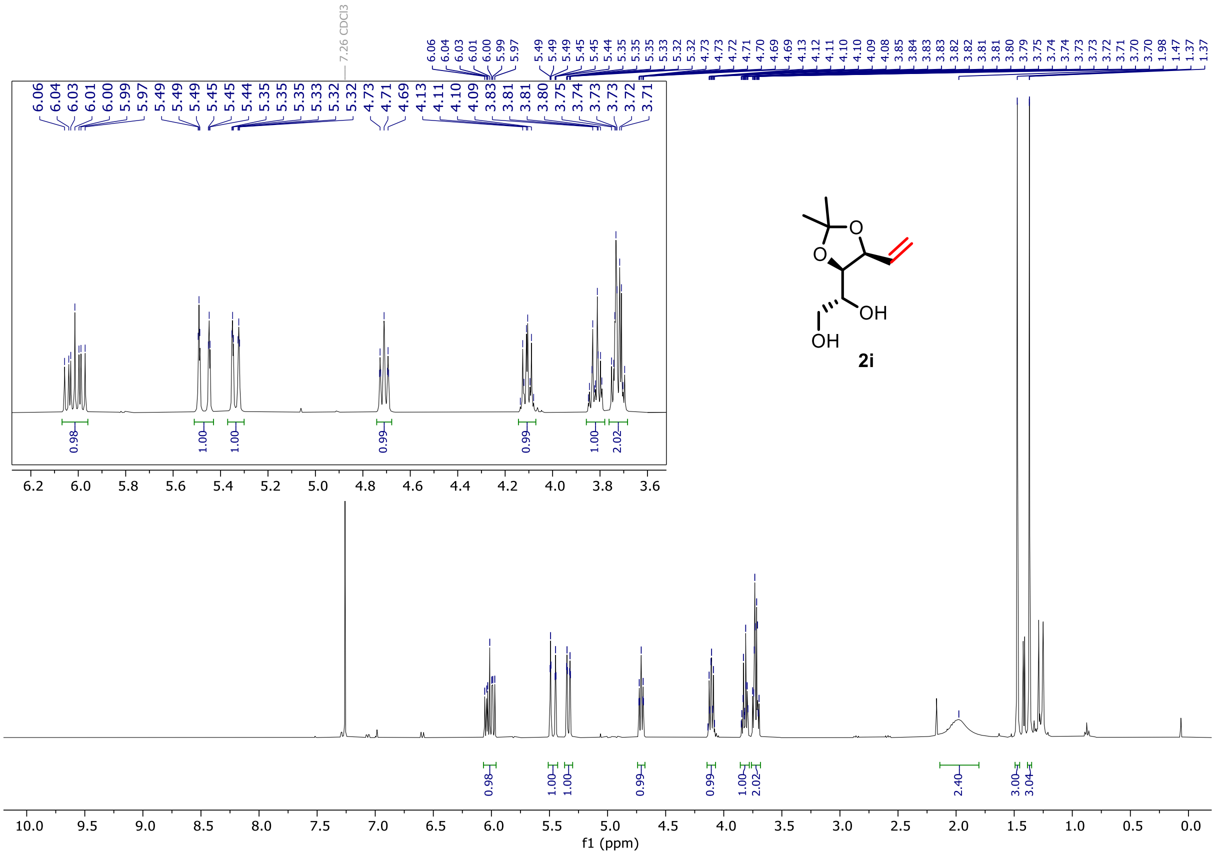

Figure S30. *400 MHz ^{1}H NMR of **2i***

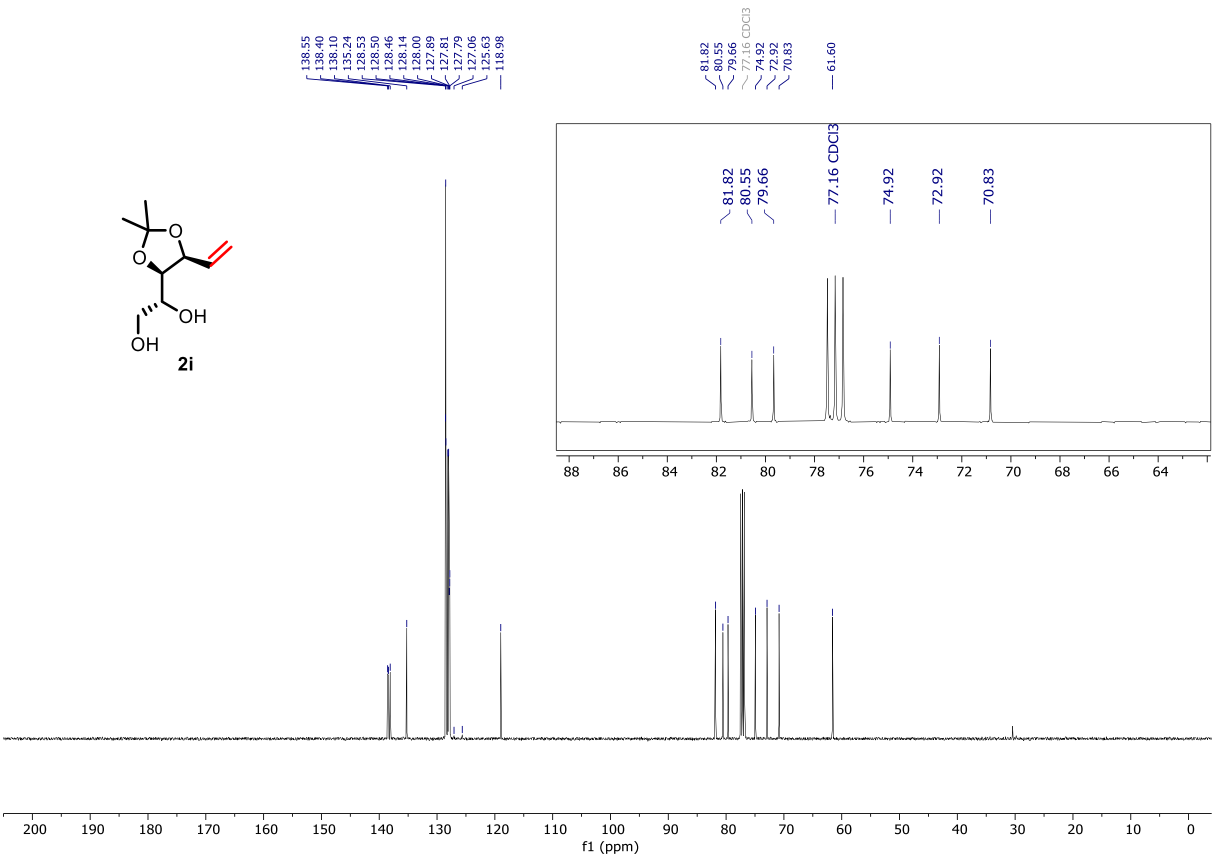

Figure S31. *101 MHz ^{13}C NMR of **2i***

H.2.10. NMR of 1,2-dideoxy-3,4-*O*-(1-methylethylidene)-L-*erythro*-pent-1-enitol (2j)

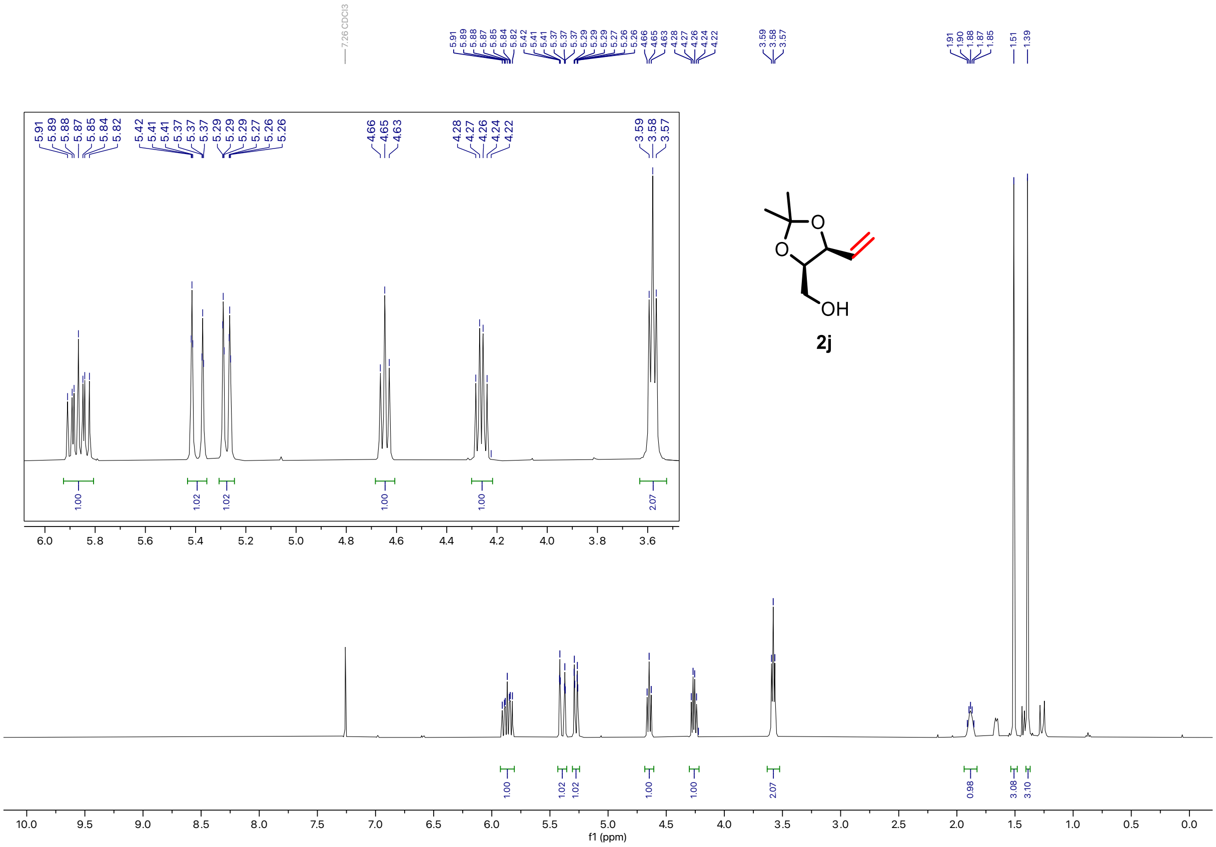

Figure S32. 400 MHz ^1H NMR of ***2j***

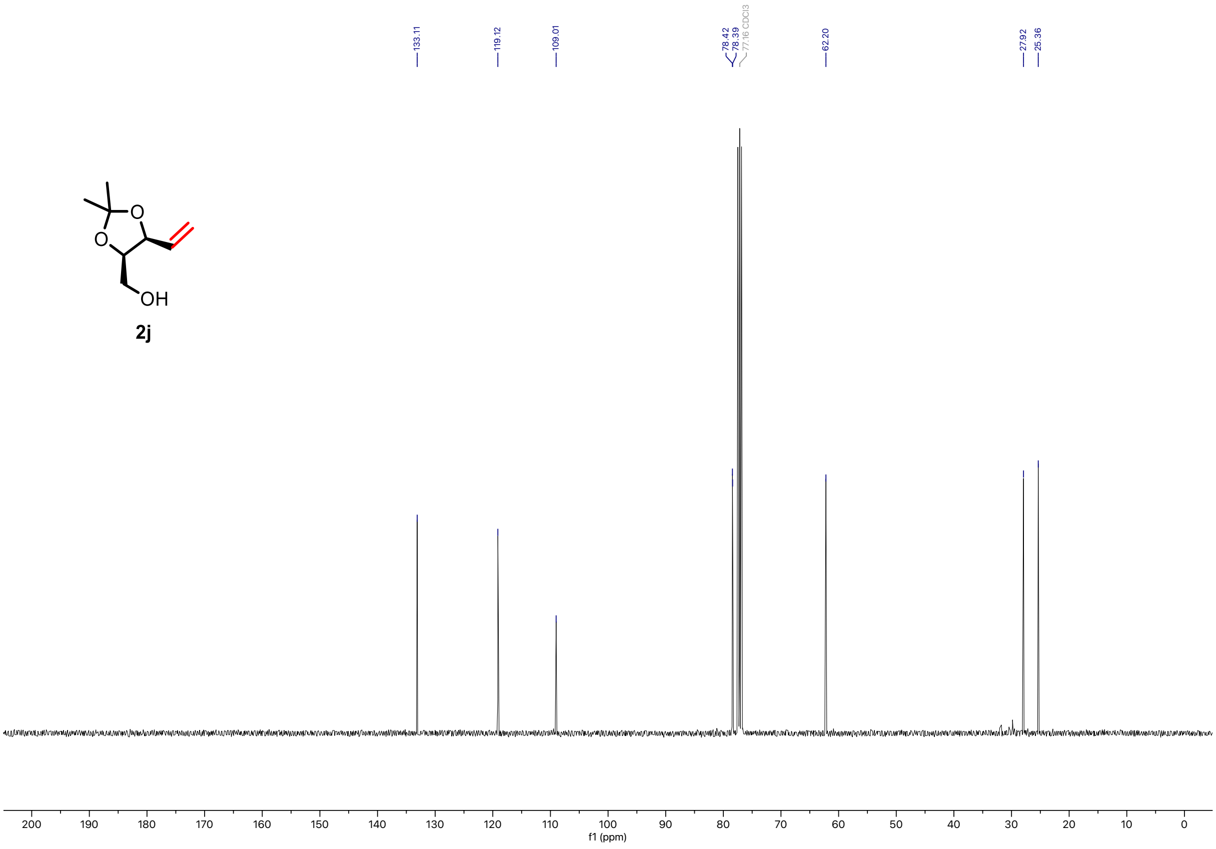

Figure S33. 101 MHz ^{13}C NMR of ***2j***

H.2.11. (1,1-$^{2}H_2$)-1,2-Dideoxy-3,4;6,7-di-*O*-isopropylidene-D-*manno*-hept-1-enitol (2k)

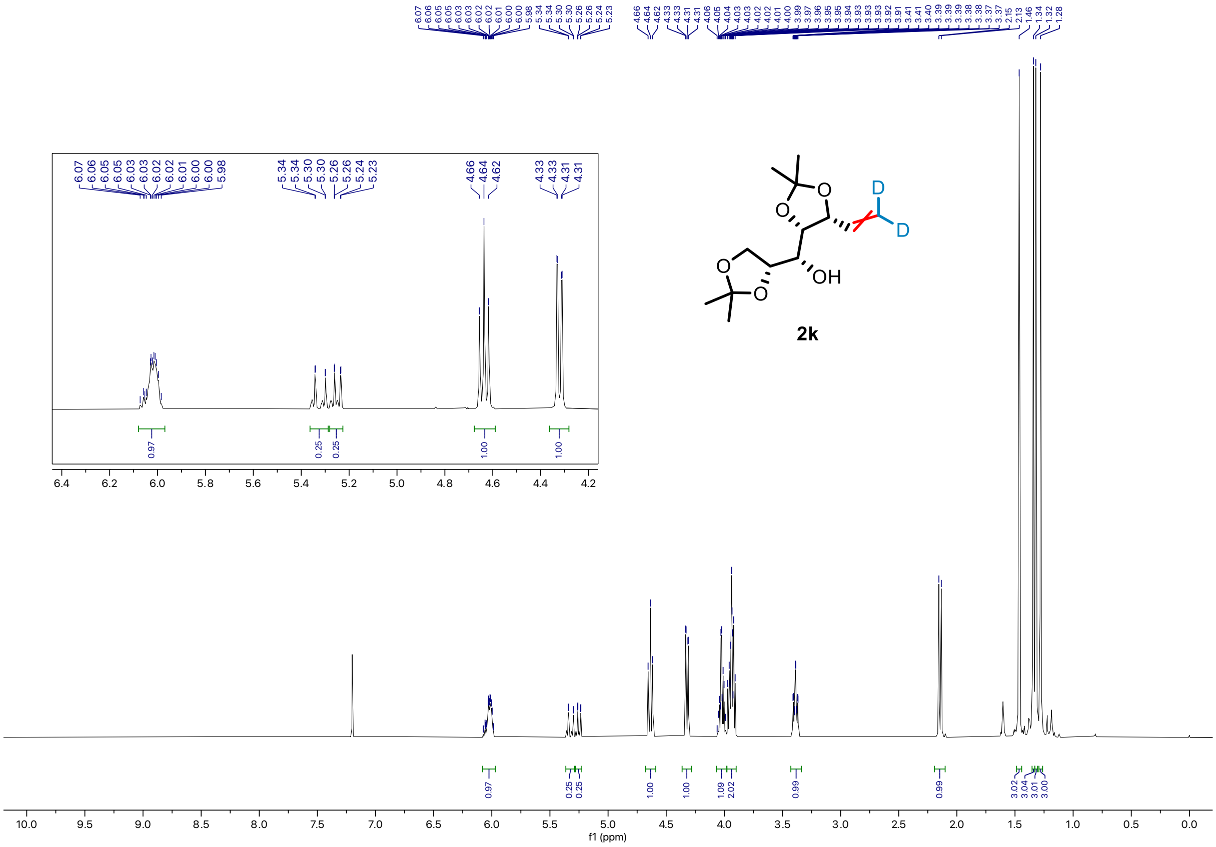

Figure S34. *400 MHz ^{1}H NMR of* ***2k*** *(D% = 75%)*

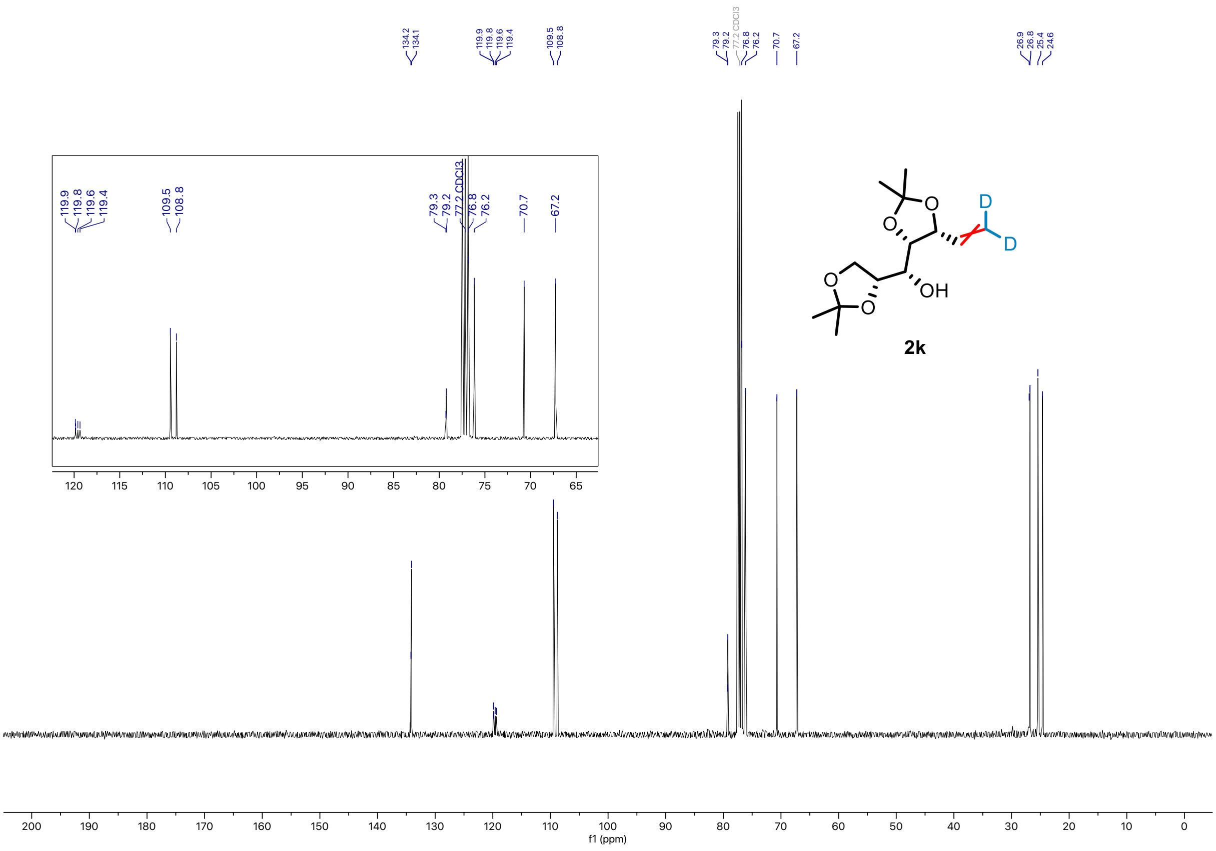

Figure S35. *101 MHz^{13}C NMR of* ***2k*** *(D% = 75%)*

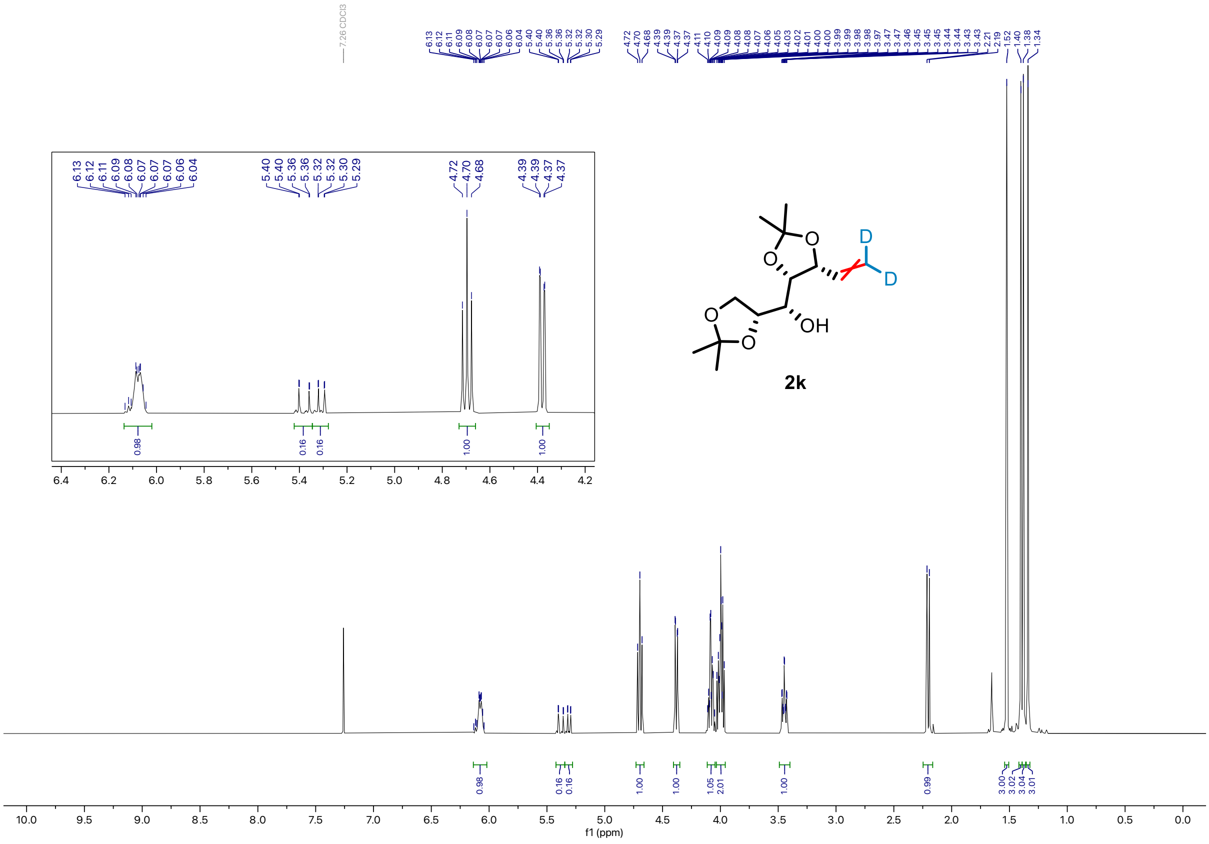

Figure S36. *400 MHz ^{1}H NMR of **2k** (D% = 84%)*

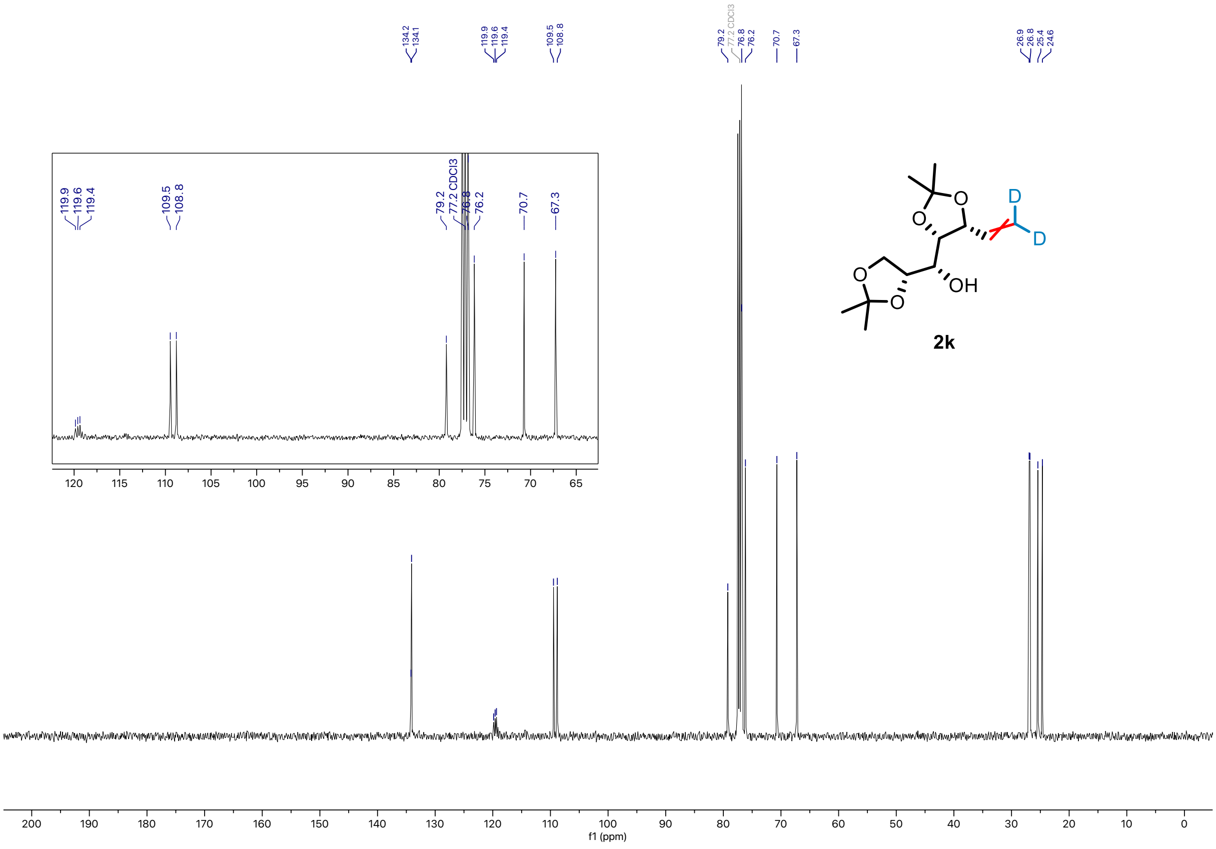

Figure S37. *101 MHz^{13}C NMR of **2k** (D% = 84%)*

H.2.12. (*R*)-[(*R*)-2,2-Dimethyl-1,3-dioxolan-4-yl][(4*S*,5*R*)-2,2-dimethyl-5-(2-methylprop-1-enyl)-1,3-dioxolan-4-yl]methanol (*manno*) (2l)

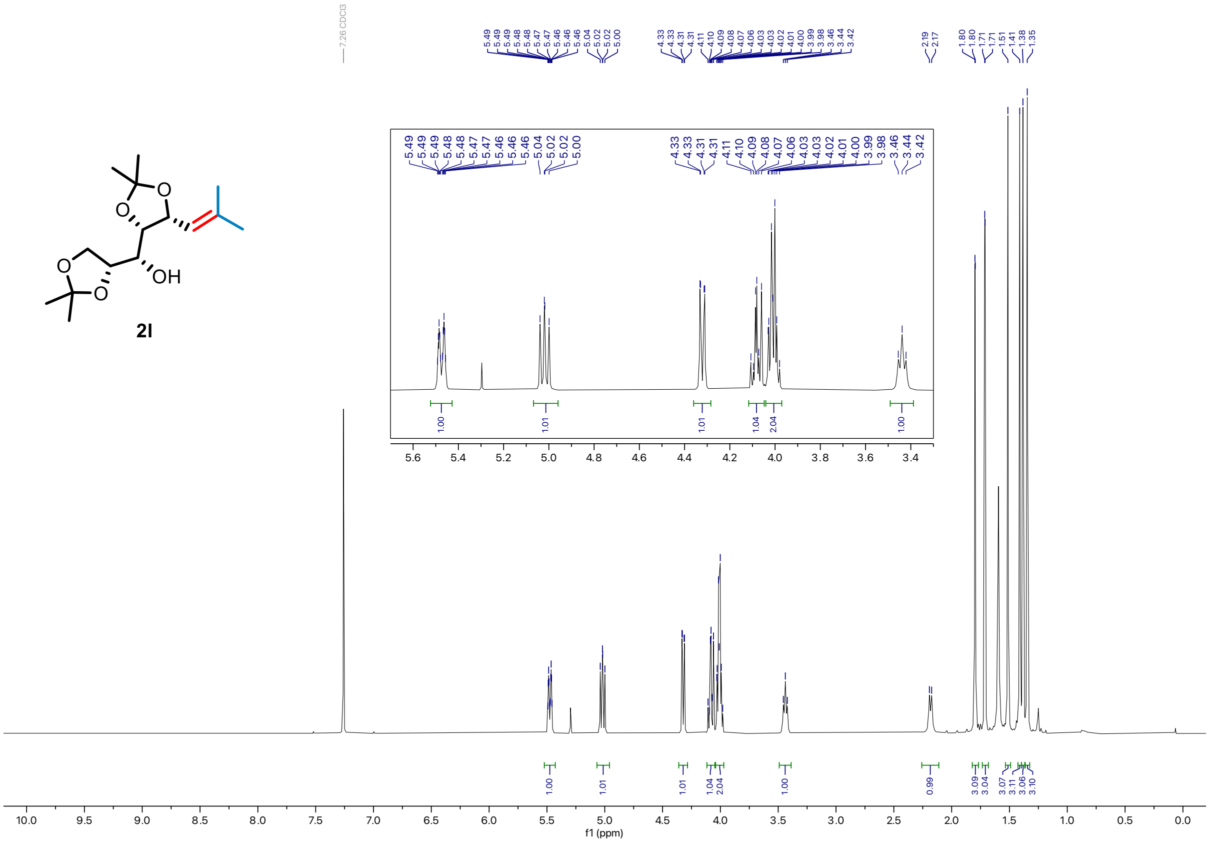

Figure S38. *400 MHz ^{1}H NMR of **2l***

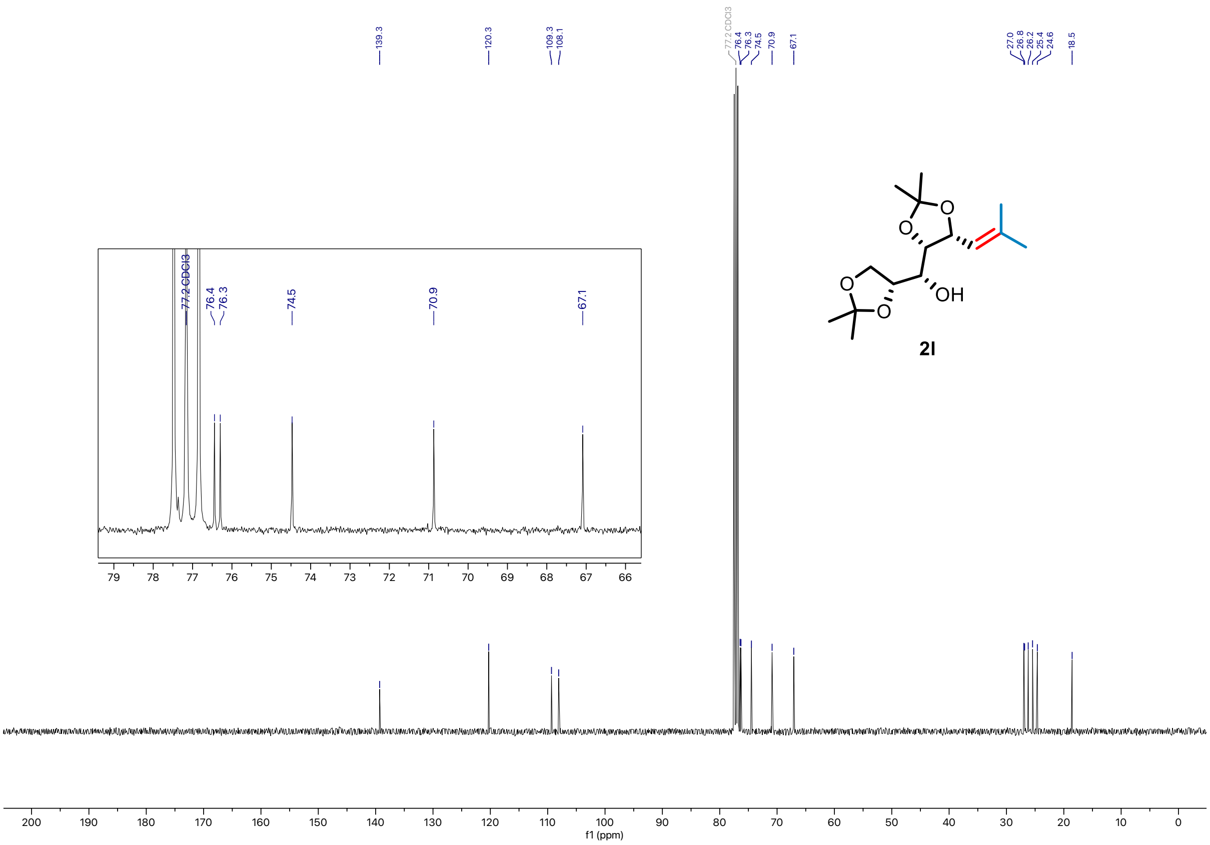

Figure S39. *151 MHz ^{13}C NMR of **2l***

H.2.13. NMR of (2*R*,3*R*,4*R*,5*R*)-3-Hydroxy-1,2;4,5-di-*O*-isopropylidene-6-decene (*manno*) (2m)

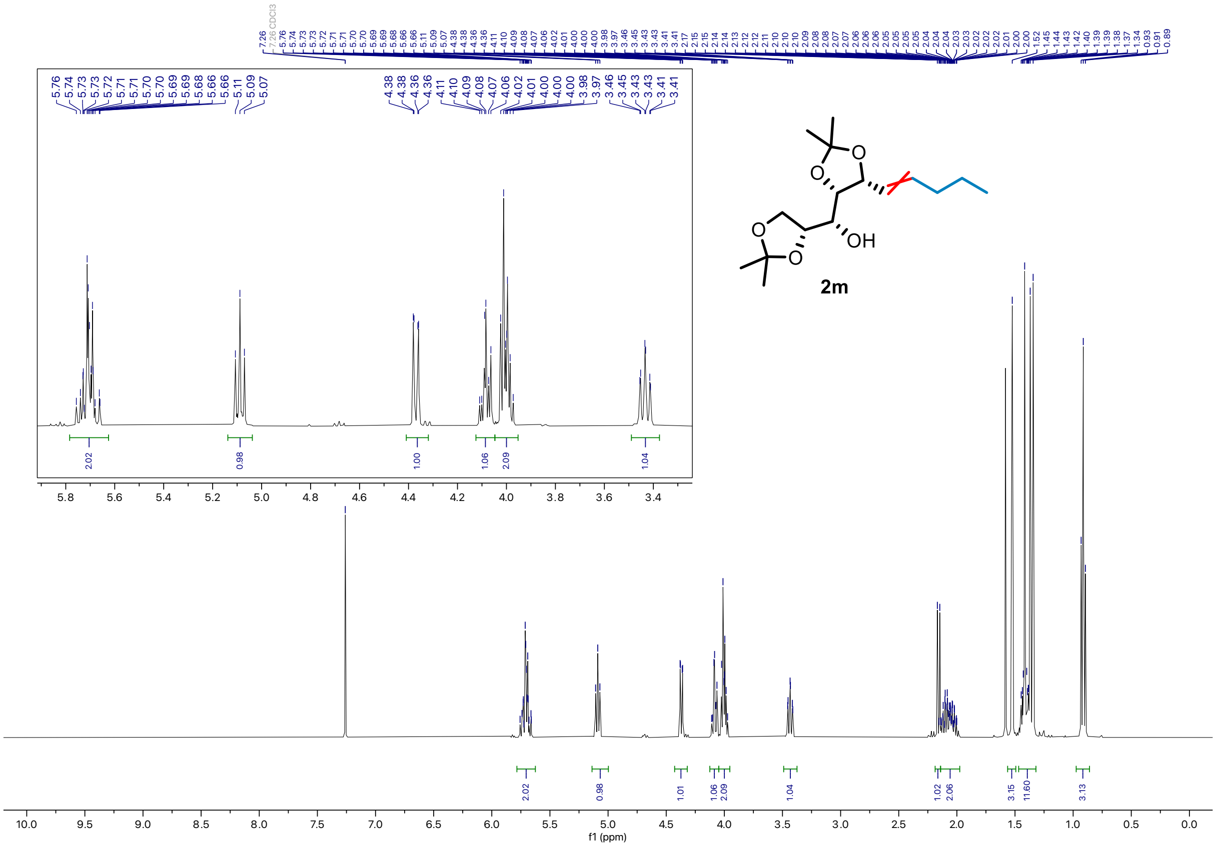

Figure S40. *400 MHz ^{1}H NMR of **2m***

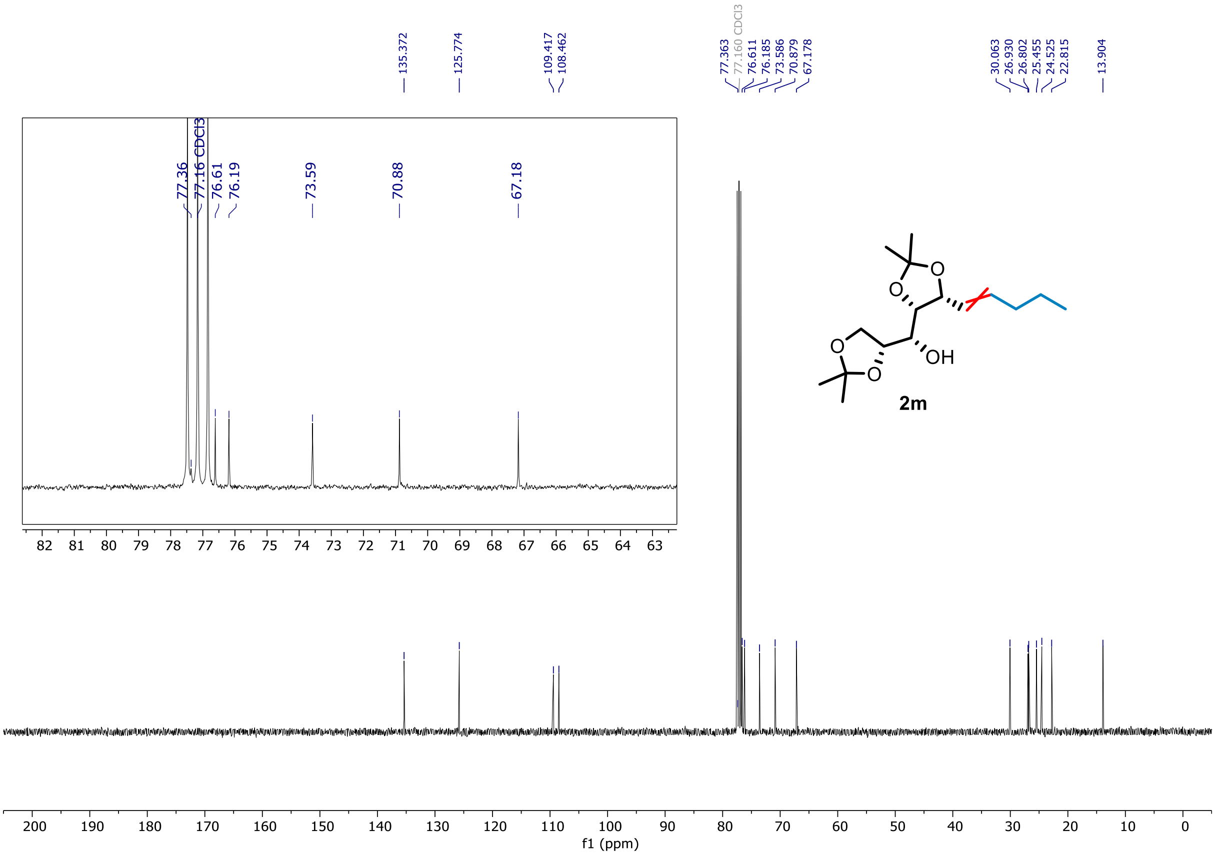

Figure S41. *101 MHz ^{13}C NMR of **2m***

H.2.14. NMR of (*Z*)-5-[(4*R*,5*S*)-5-{(*R*)-[(*R*)-2,2-dimethyl-1,3-dioxolan-4-yl]hydroxymethyl}-2,2-dimethyl-1,3-dioxolan-4-yl]pent-4-enenitrile (*manno*) (2n)

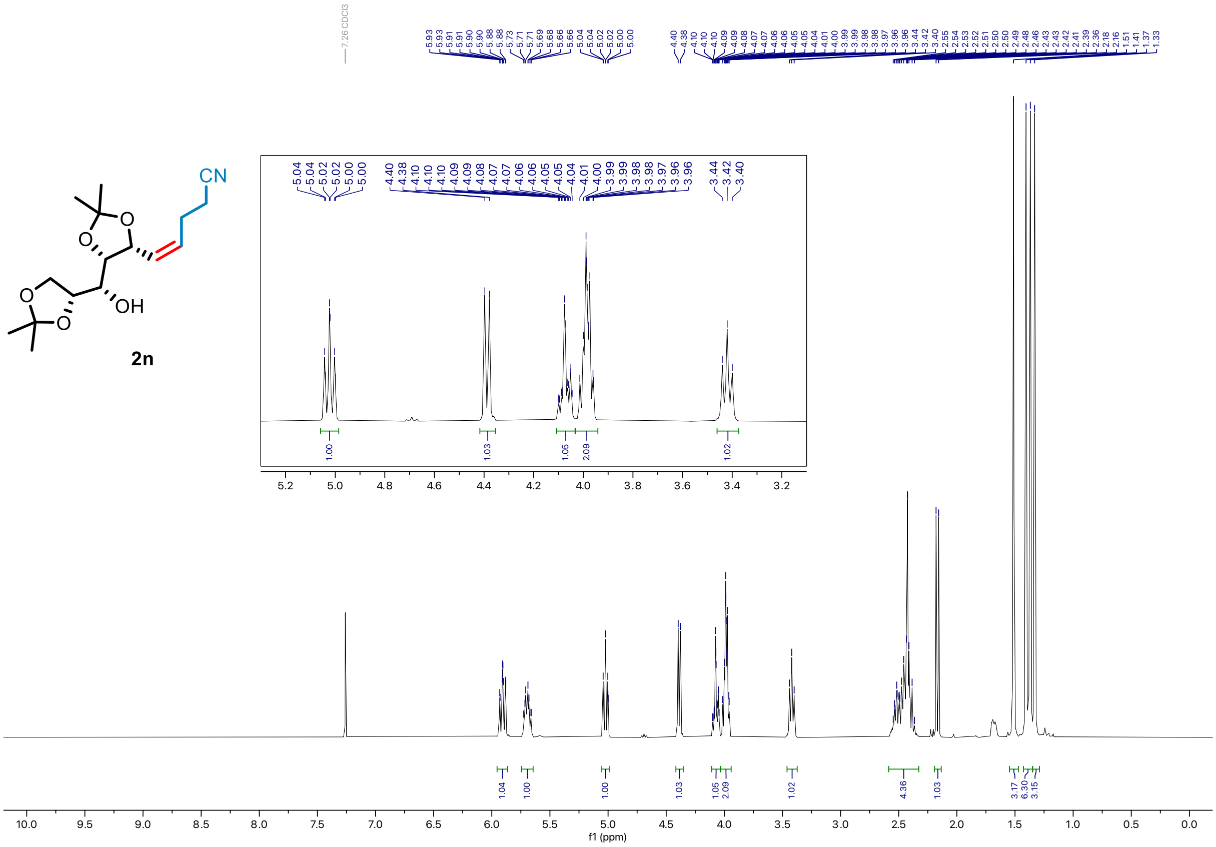

Figure S42. *400 MHz ^{1}H NMR of* ***2n***

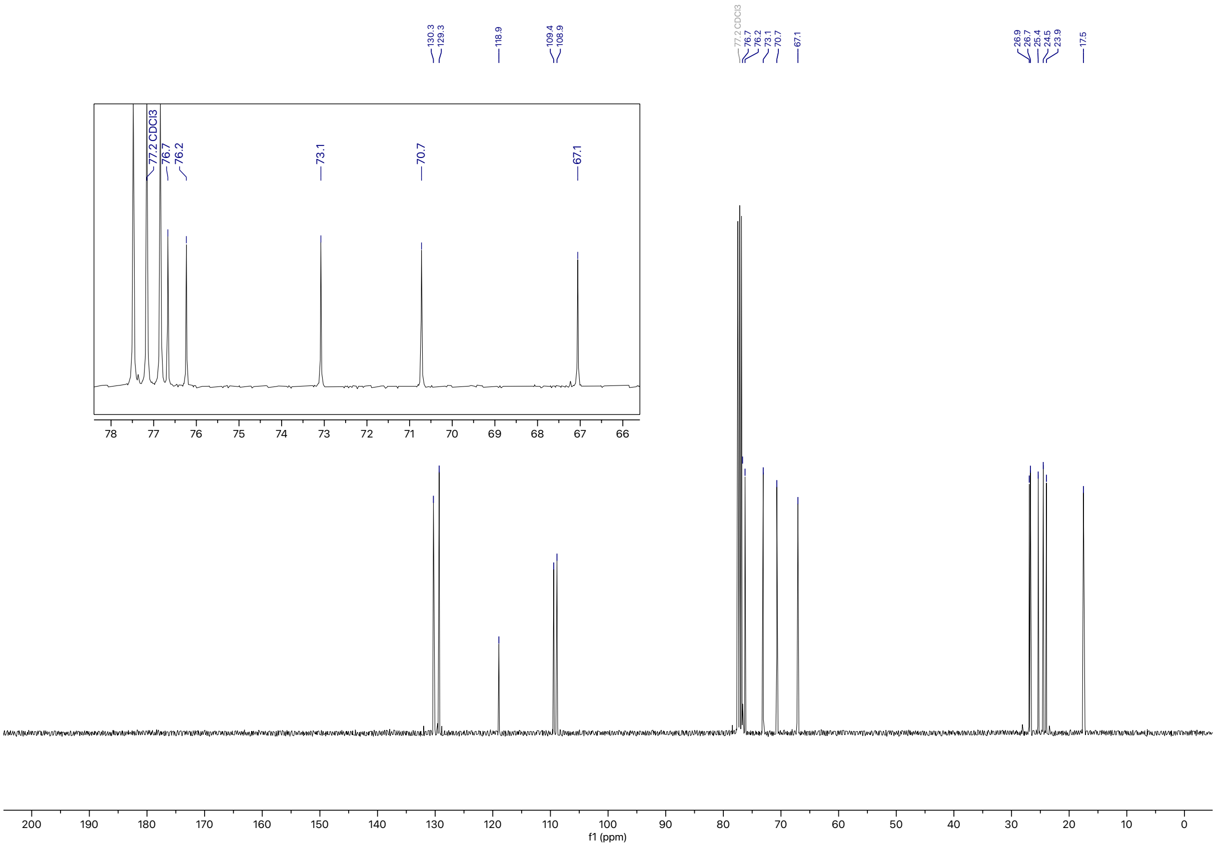

Figure S43. *101 MHz ^{13}C NMR of* ***2n***

H.2.15. NMR of (*R*)-((4*S*,5*R*)-5-((*E*)-buta-1,3-dien-1-yl)-2,2-dimethyl-1,3-dioxolan-4-yl)((*R*)-2,2-dimethyl-1,3-dioxolan-4-yl)methanol (*manno*) (2o)

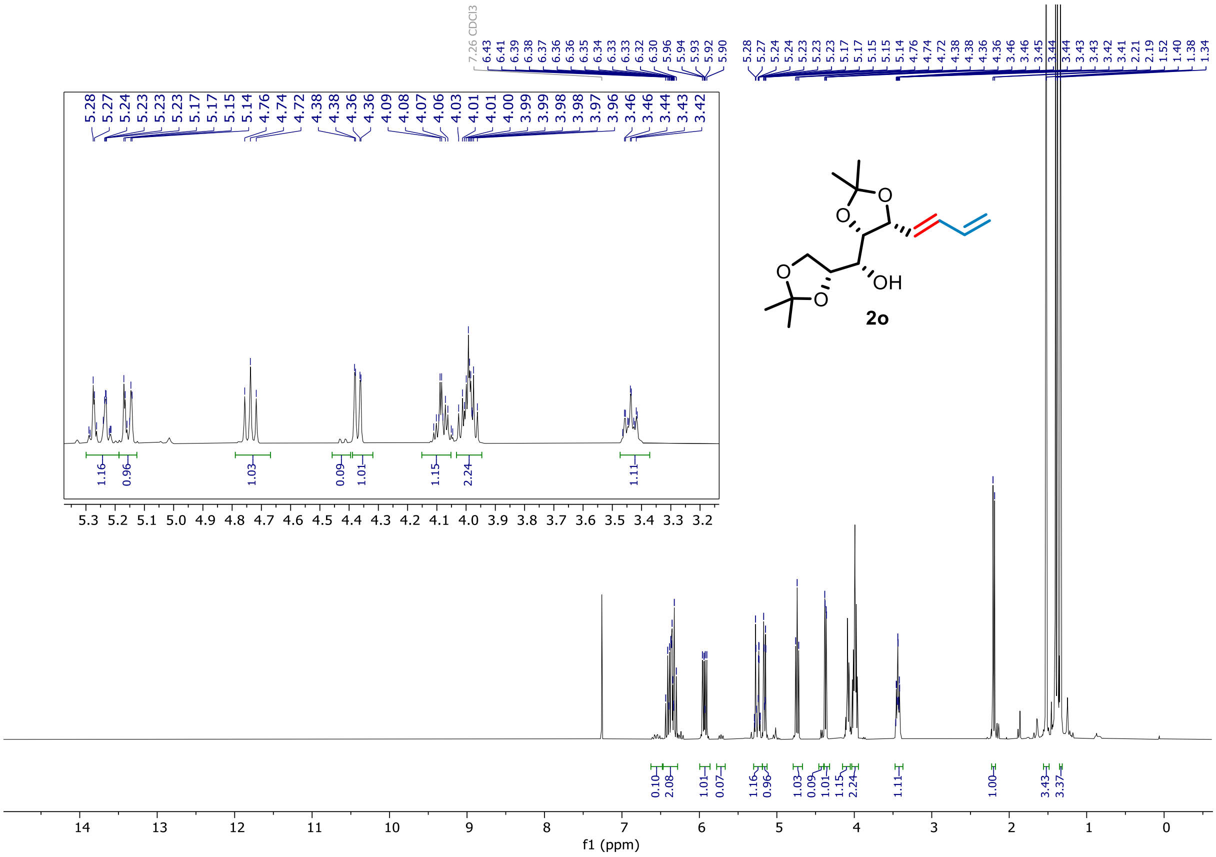

Figure S44. *400 MHz ^{1}H NMR of **2o***

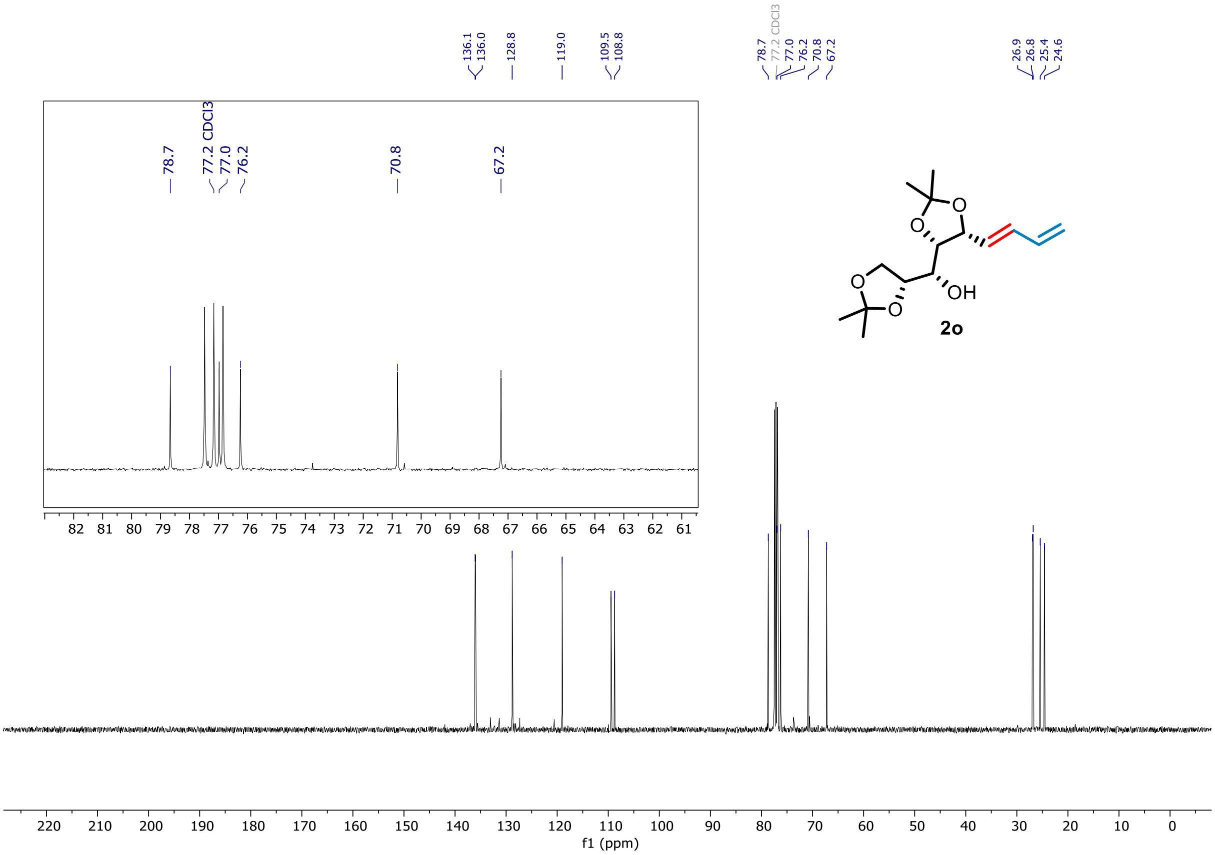

Figure S45. *101 MHz ^{13}C NMR of **2o***

H.2.16. 1,2-Dideoxy-3,4;6,7-di-*O*-isopropylidene-1-phenyl-D-*manno*-hept-1-enitol (2p)

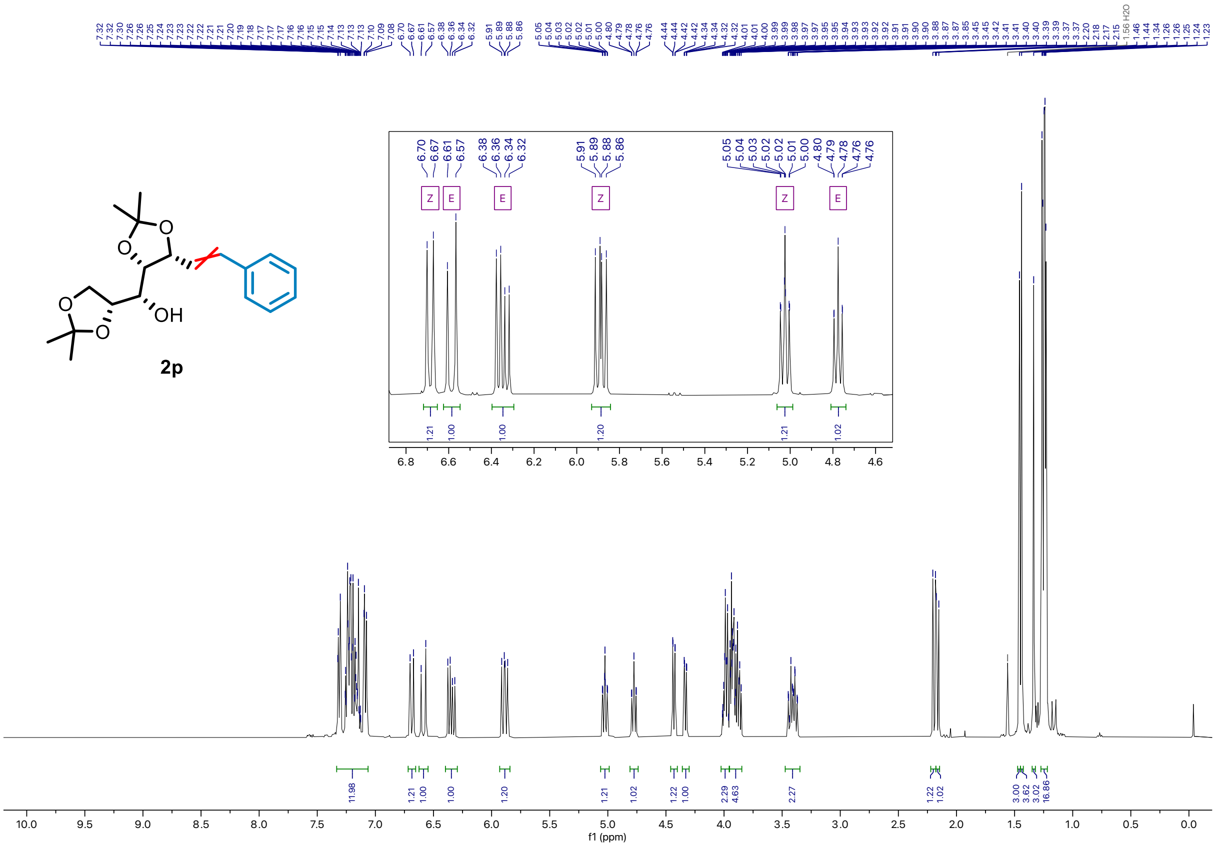

Figure S46. *400 MHz* ^{1}H *NMR of* ***2p***

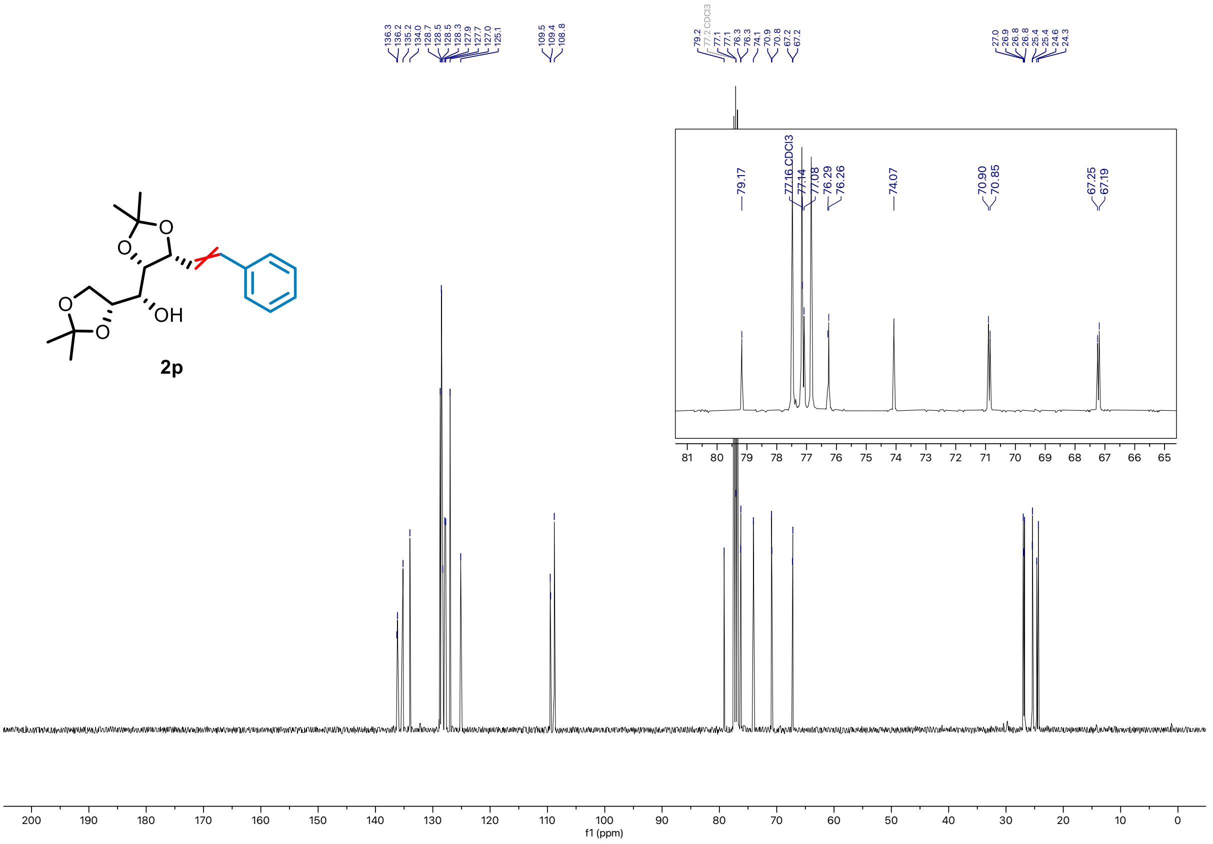

Figure S47. *101 MHz* ^{13}C *NMR of* ***2p***

H.2.17. 1-(2,4-Dichlorophenyl)-1,2-dideoxy-3,4;6,7-di-*O*-isopropylidene-D-*manno*-hept-1-enitol (2q)

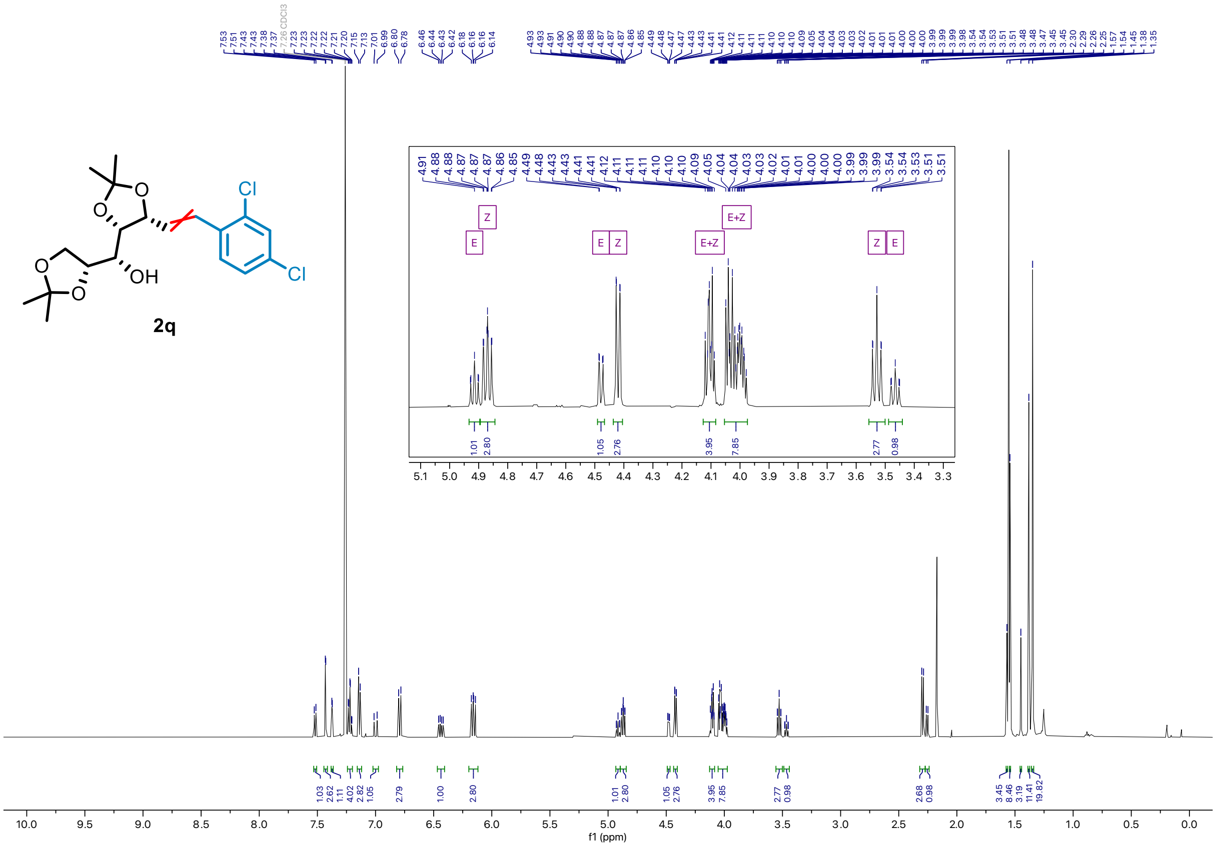

Figure S48. *600 MHz* ^1H *NMR of* ***2q***

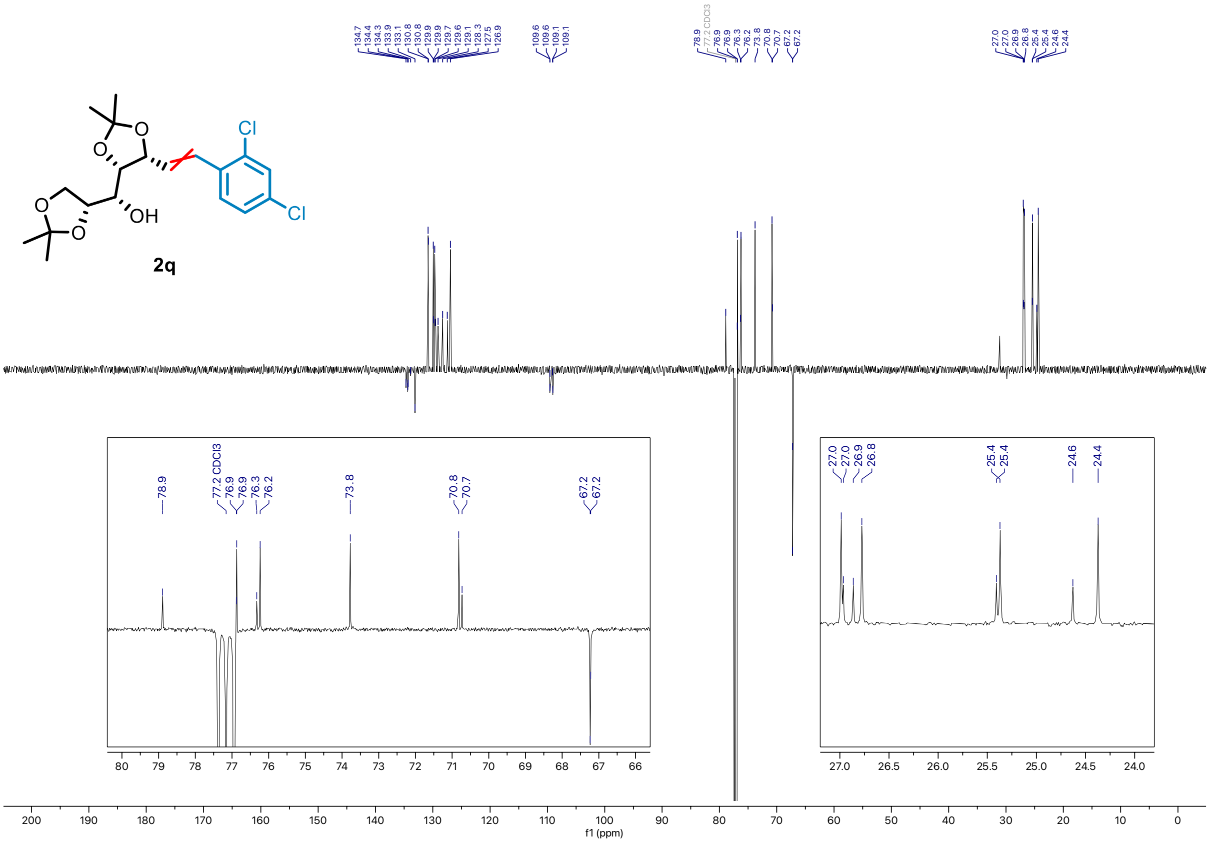

Figure S49. *151 MHz* ^{13}C *NMR of* ***2q***

H.2.18. NMR of (*R*)-((*R*)-2,2-dimethyl-1,3-dioxolan-4-yl)((4*S*,5*R*)-5-(2-methoxyvinyl)-2,2-dimethyl-1,3-dioxolan-4-yl)methanol (*manno*) (2r)

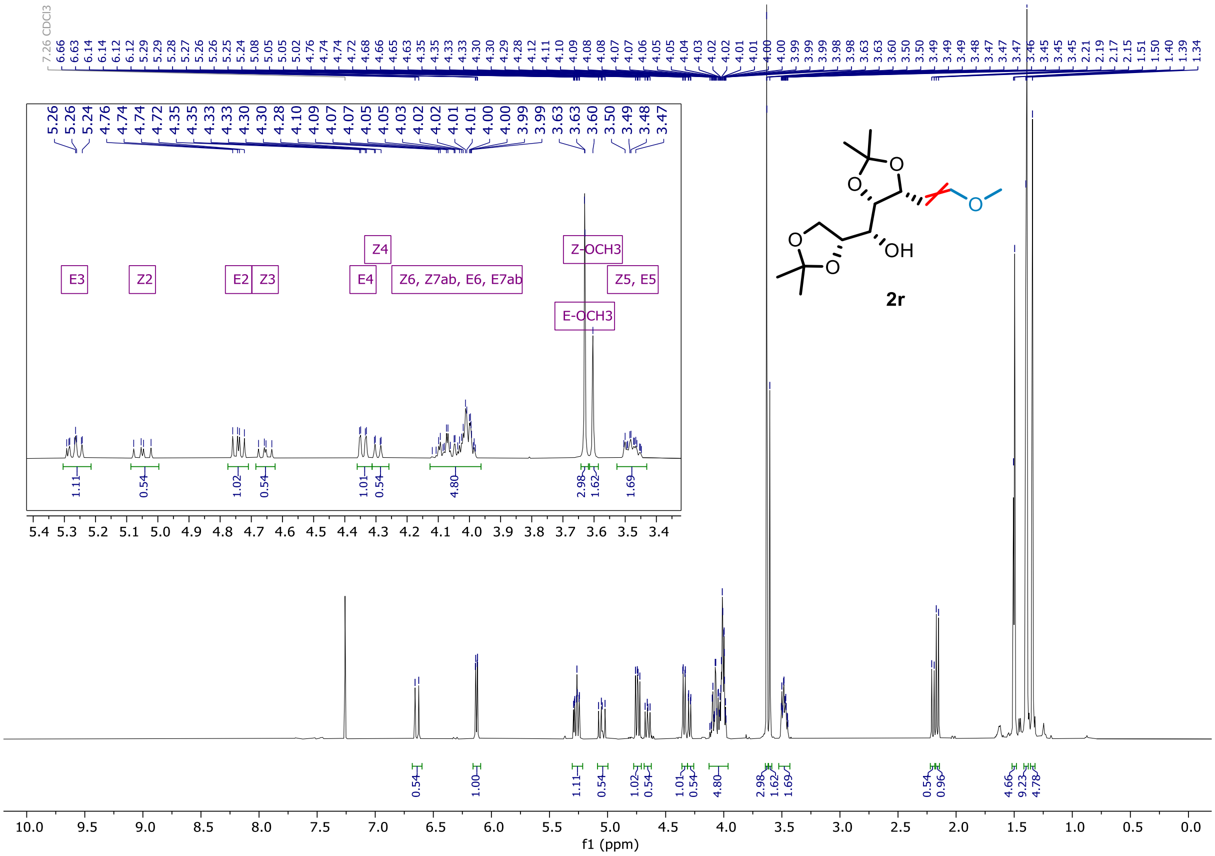

Figure S50. *400 MHz ^{1}H NMR of* ***2r***

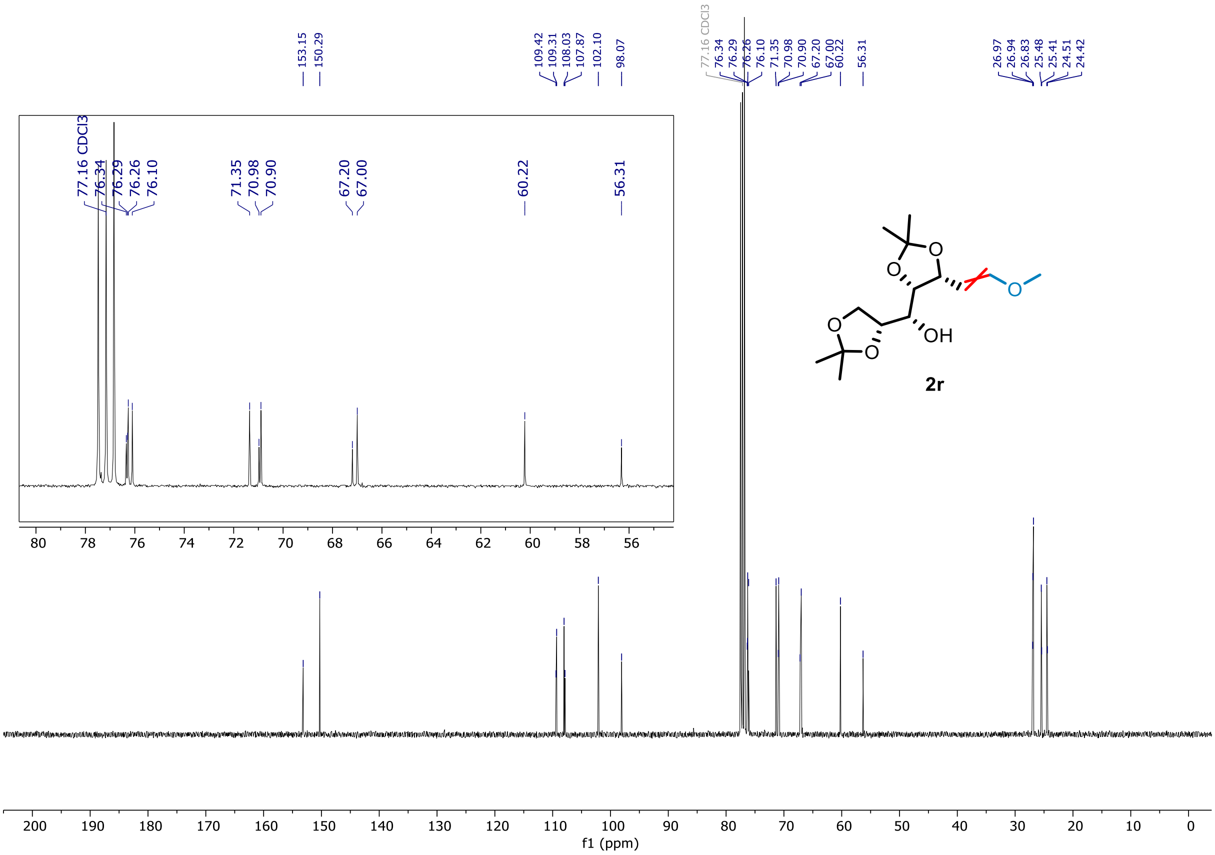

Figure S51. *101 MHz ^{13}C NMR of* ***2r***

H.2.19. NMR of (*R*)-((4*S*,5*R*)-5-(2-(1*H*-benzo[*d*][1,2,3]triazol-1-yl)vinyl)-2,2-dimethyl-1,3-dioxolan-4-yl)((*R*)-2,2-dimethyl-1,3-dioxolan-4-yl)methanol (*manno*) (2s)

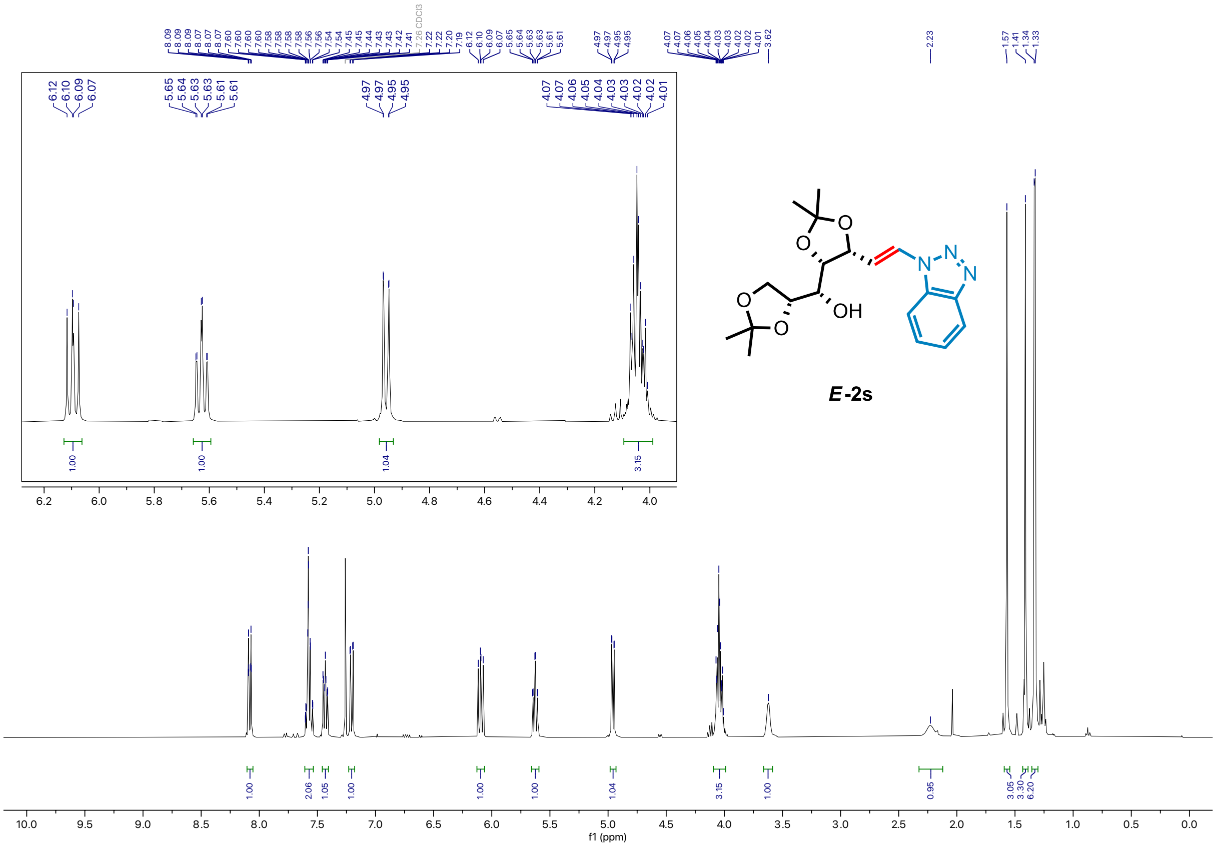

Figure S52. *400 MHz ^{1}H NMR of **2s** – E isomer*

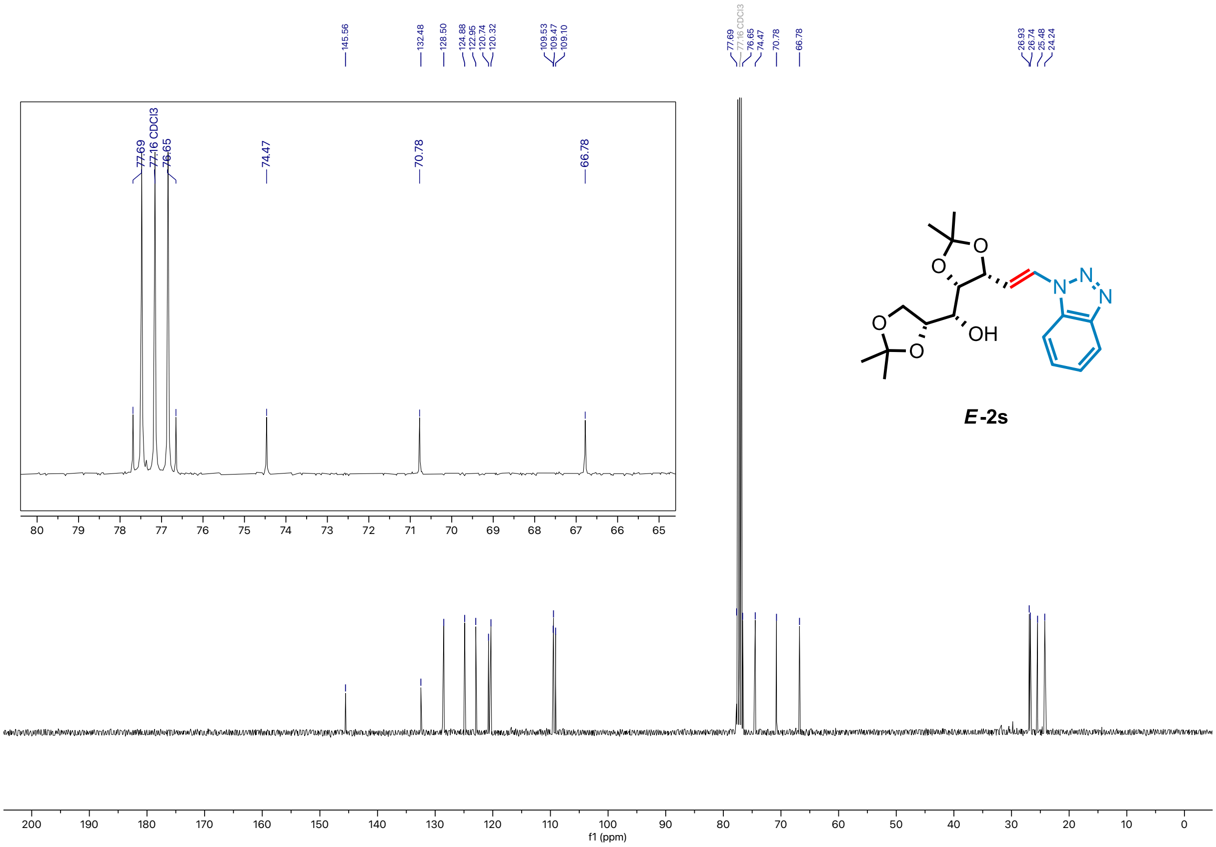

Figure S53. *101 MHz ^{13}C NMR of **2s** – E-isomer*

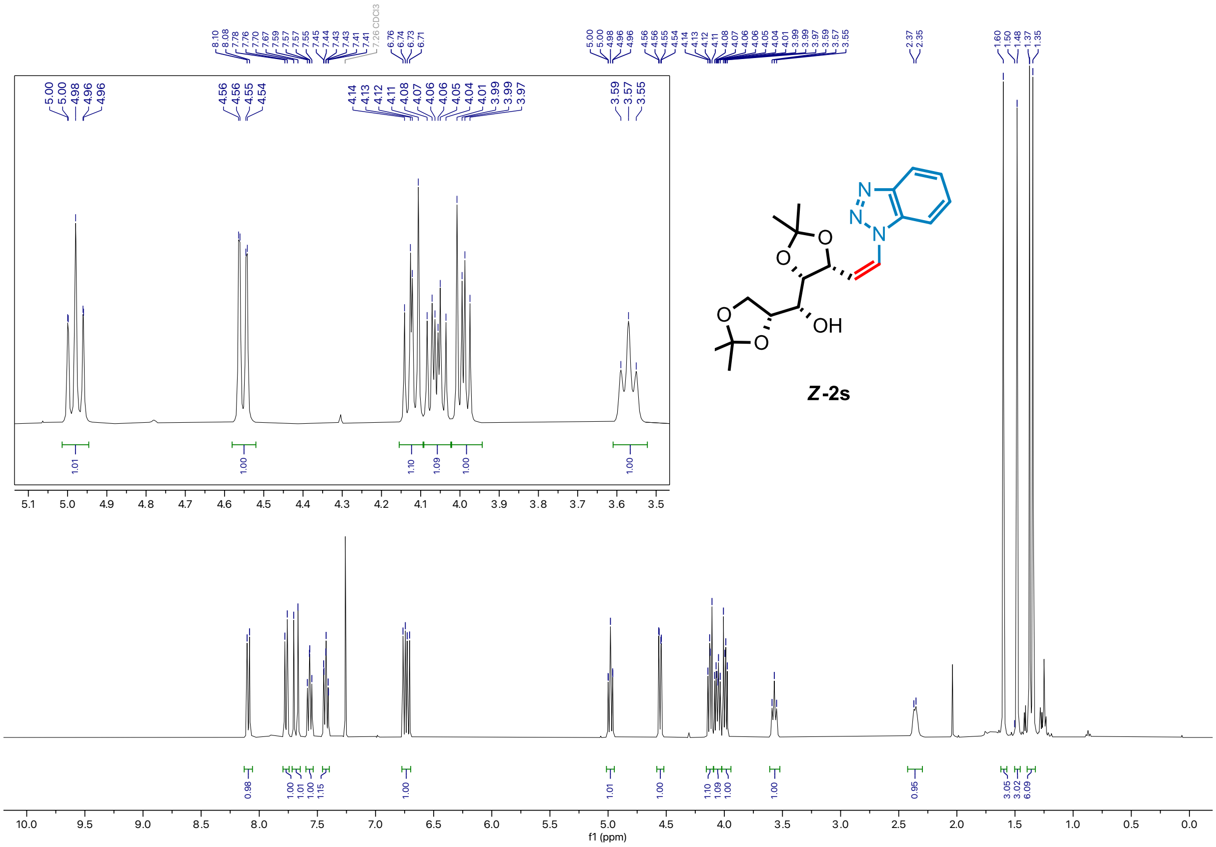

Figure S54. *400 MHz* ^{1}*H NMR of* ***2s*** *– Z isomer*

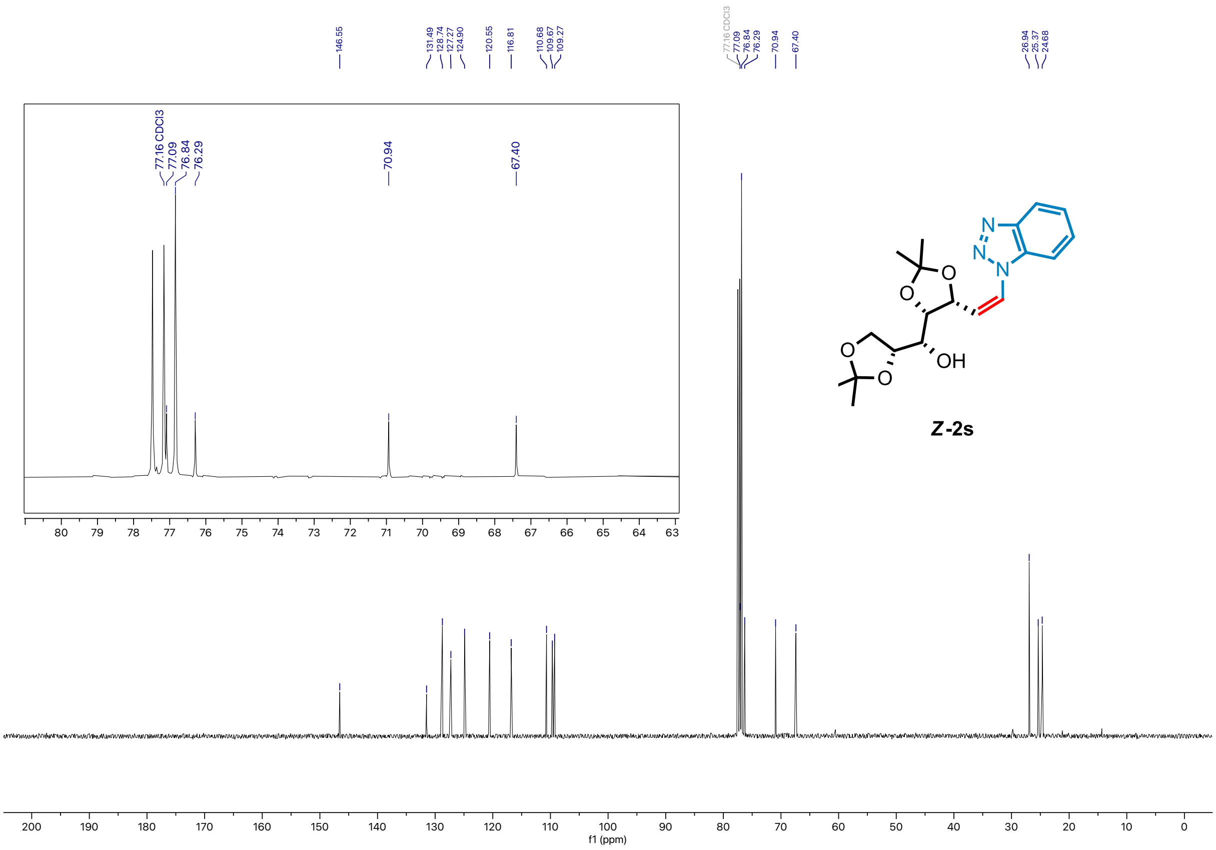

Figure S55. *101 MHz* ^{13}*C NMR of* ***2s*** *– Z-isomer*

H.2.20. (*E*)-1-Chloro-1,2-dideoxy-3,4;6,7-di-*O*-isopropylidene-D-*manno*-hept-1-enitol (2t)

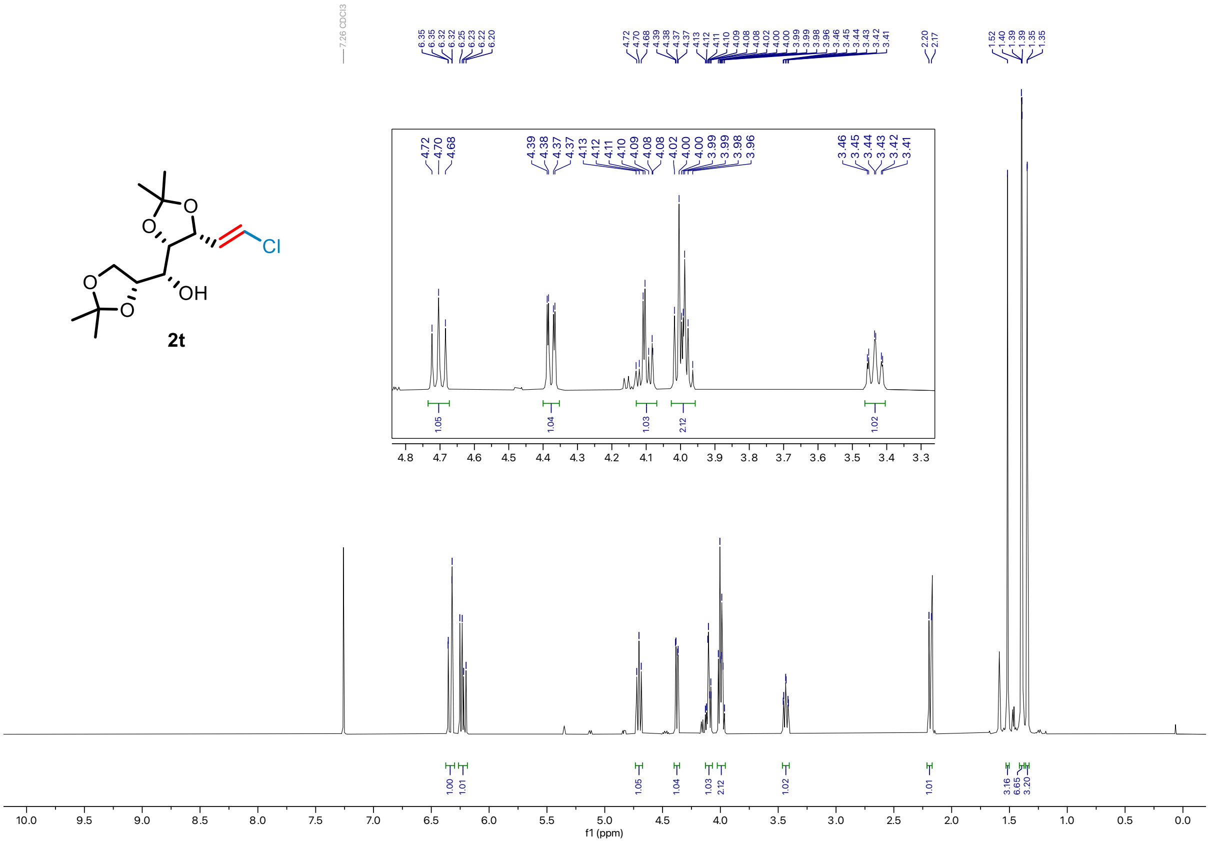

Figure S56. *400 MHz ^{1}H NMR of* ***2t***

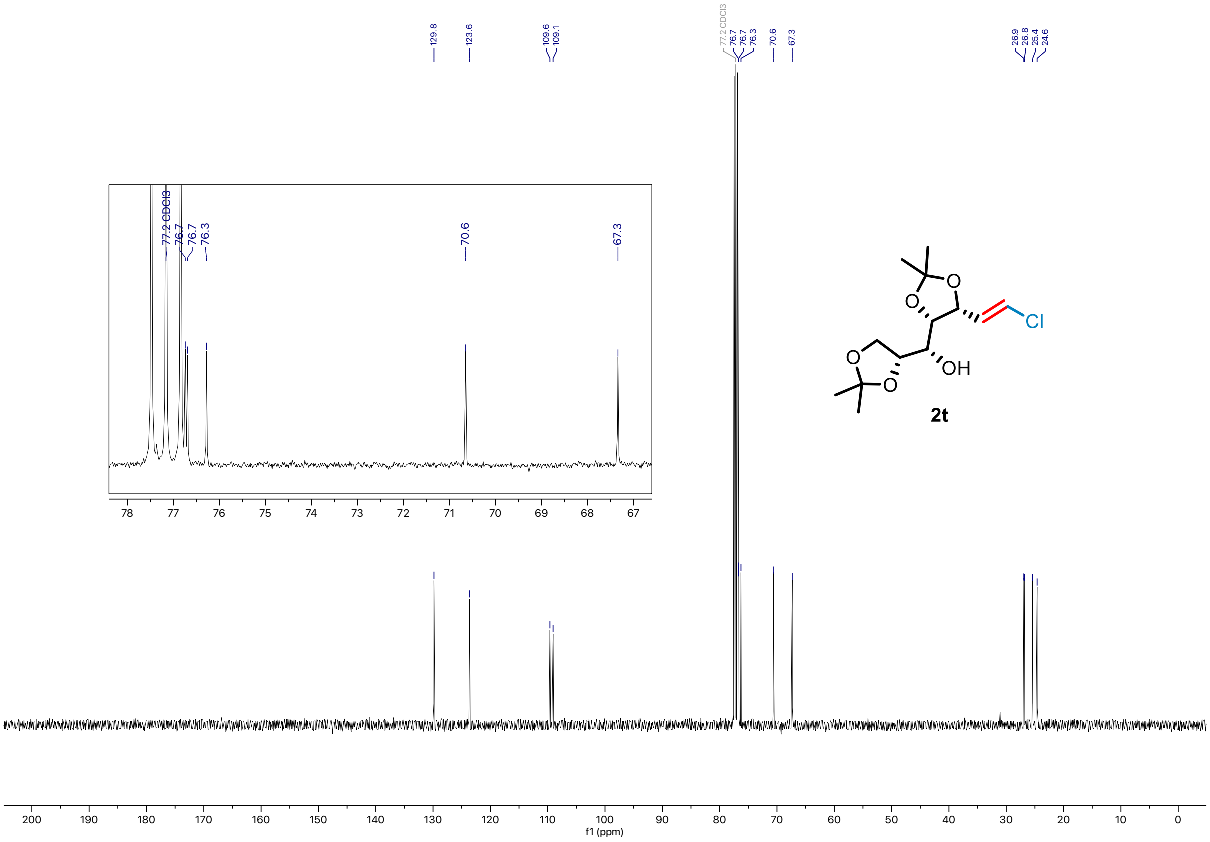

Figure S57. *101 MHz ^{13}C NMR of* ***2t***

H.2.21. NMR of (2*R*,3*R*,4*S*)-2,3,4-tris(benzyloxy)non-5-en-1-ol (*xylo*) (2u)

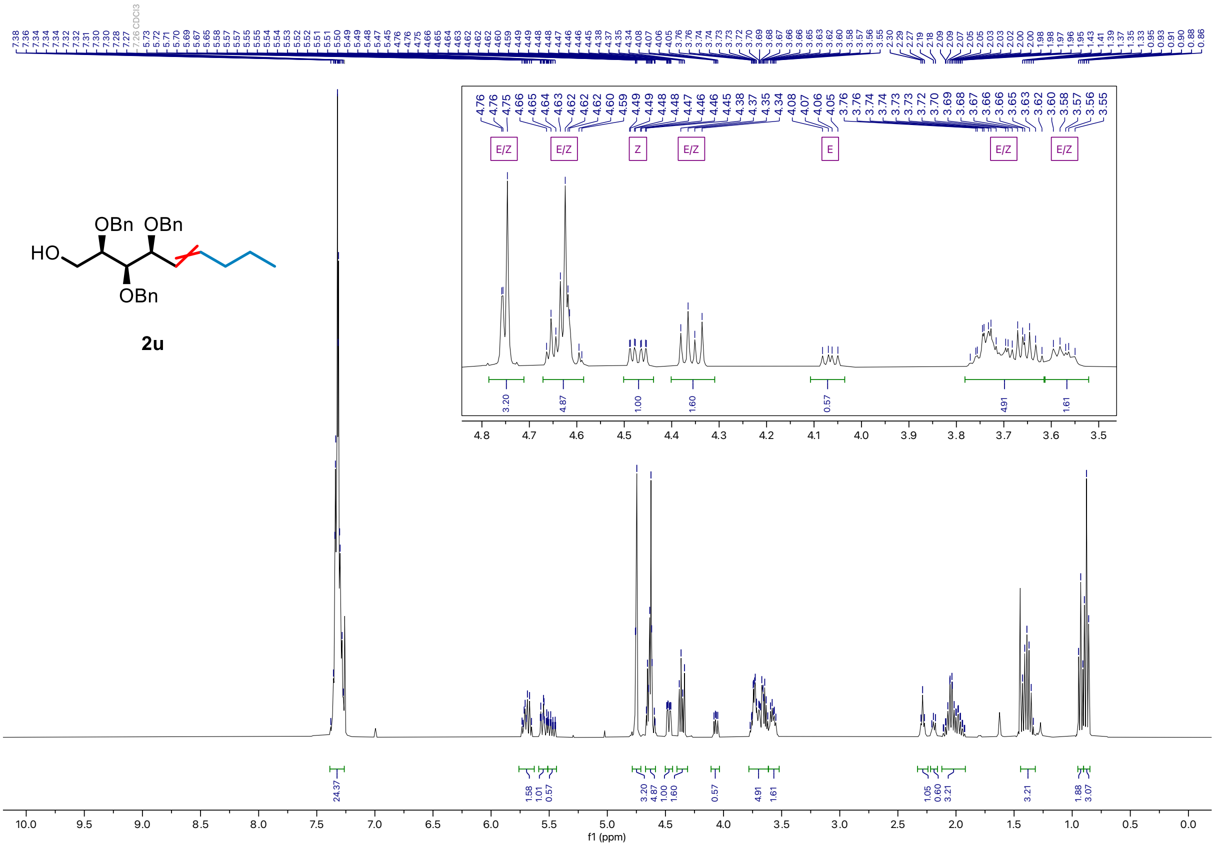

Figure S58. *400 MHz ^{1}H NMR of* ***2u***

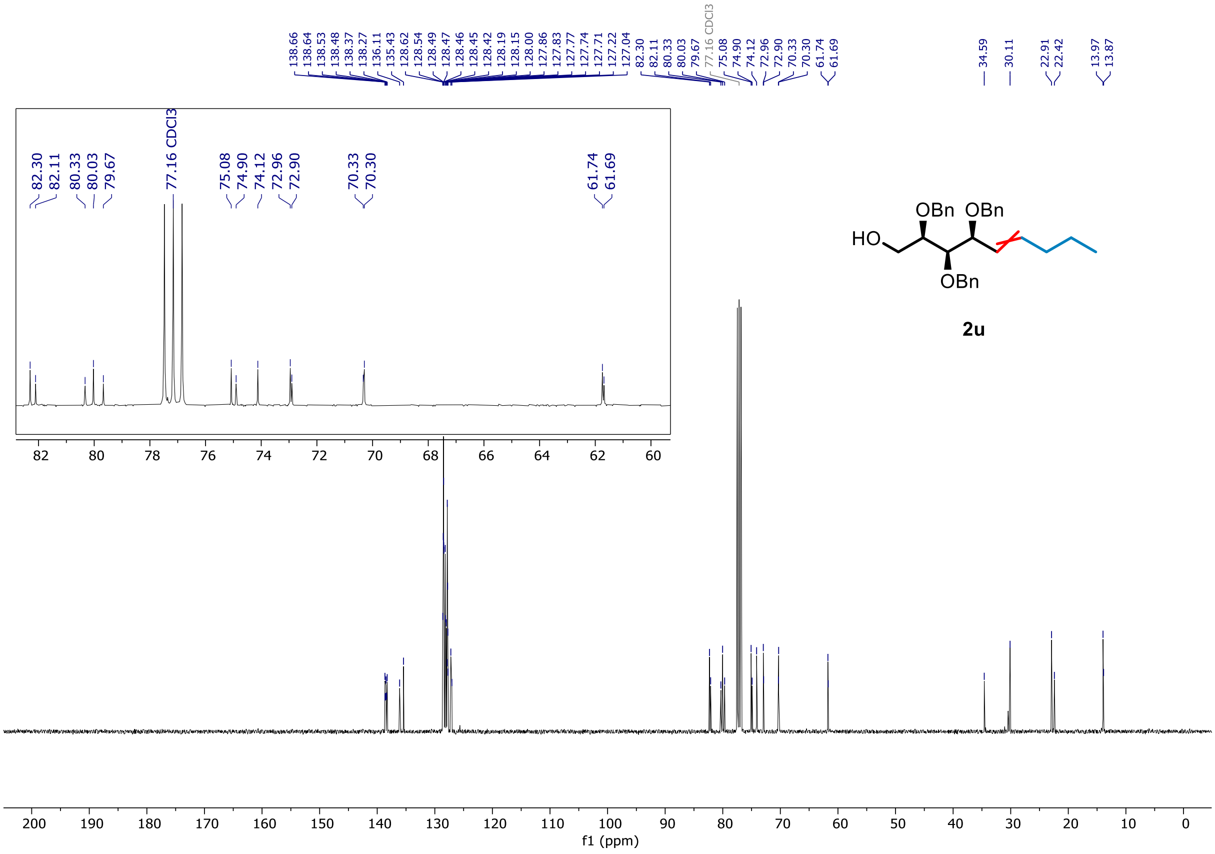

Figure S59. *101 MHz ^{13}C NMR of* ***2u***

H.2.22. NMR of (2*R*,3*R*,4*S*)-2,3,4-tris(benzyloxy)-6-cyclopropylhex-5-en-1-ol (*xylo*) (2v)

2v

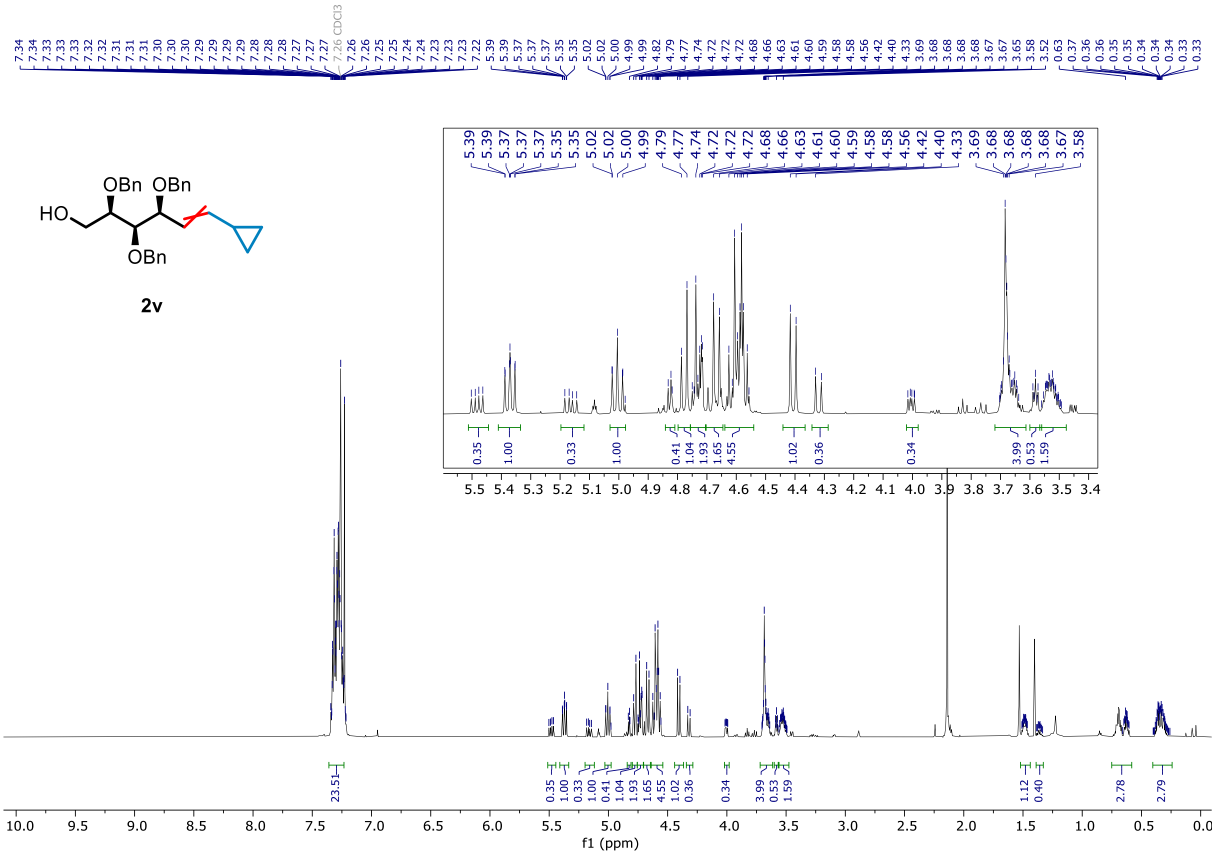

Figure S60. *600 MHz ^{1}H NMR of* ***2v***

2v

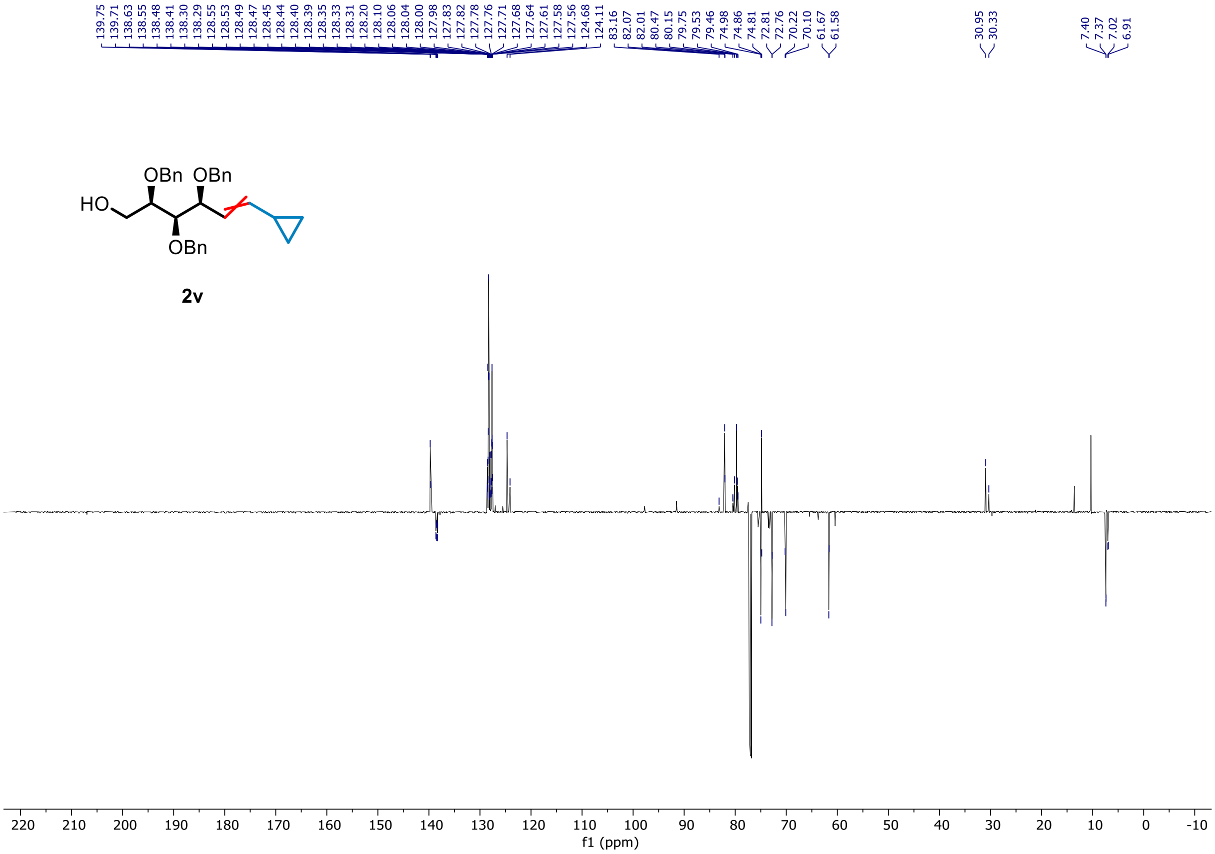

Figure S61. *151 MHz ^{13}C NMR of* ***2v***

H.3. Utilization Products

H.3.1. NMR of 1,2-dideoxy-3,4-*O*-(1-methylethylidene)-5-*O*-tosyl-L-*erythro*-pent-1-enitol (3a)

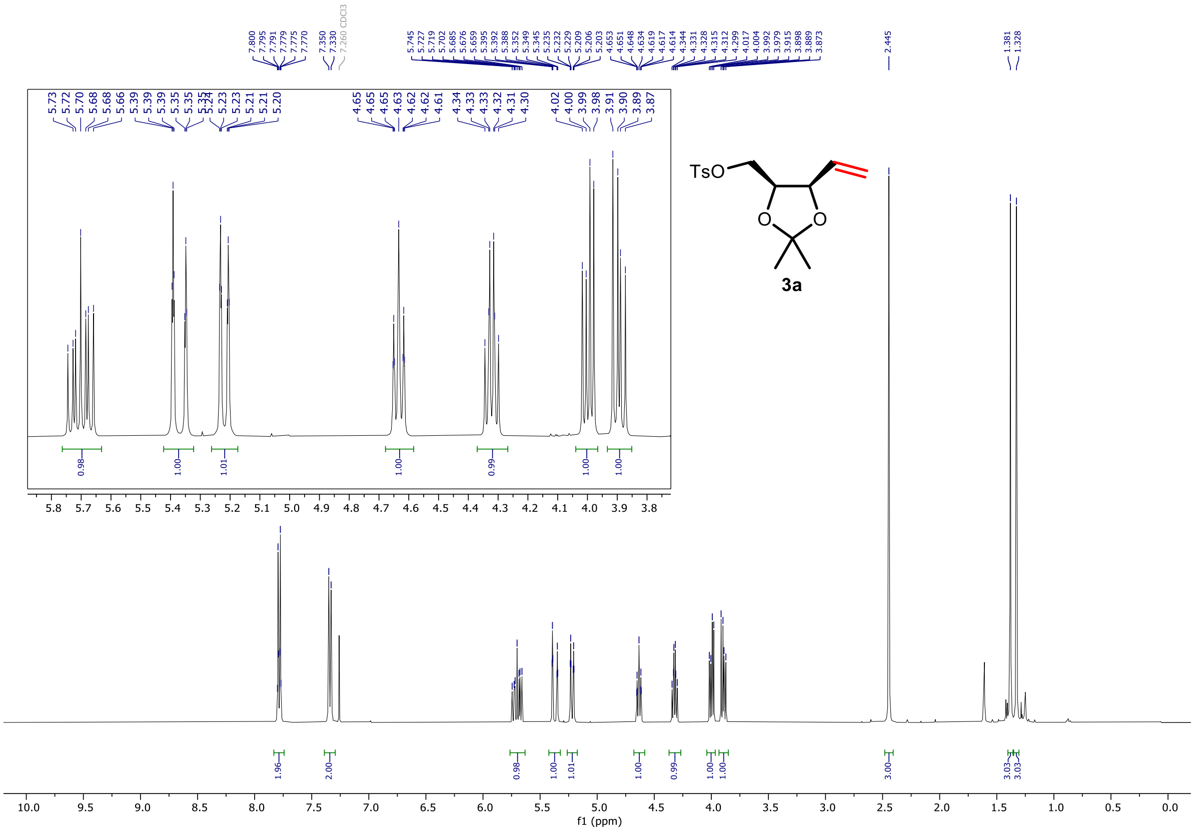

Figure S62. *400 MHz ^{1}H NMR of* ***3a***

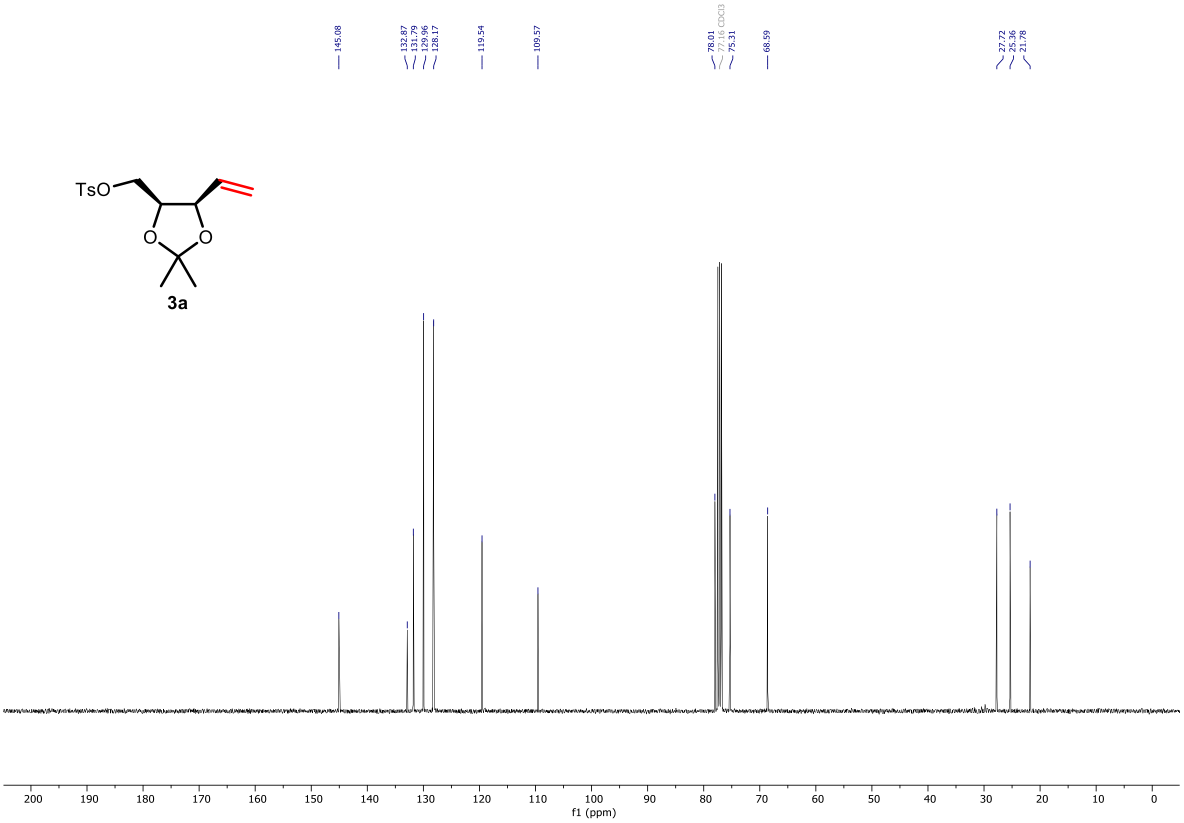

Figure S63. *101 MHz^{13}C NMR of* ***3a***

H.3.2. NMR of 2-{[(4*S*,5*R*)-2,2-dimethyl-5-vinyl-1,3-dioxolan-4-yl]methyl}isoindoline-1,3-dione (3b)

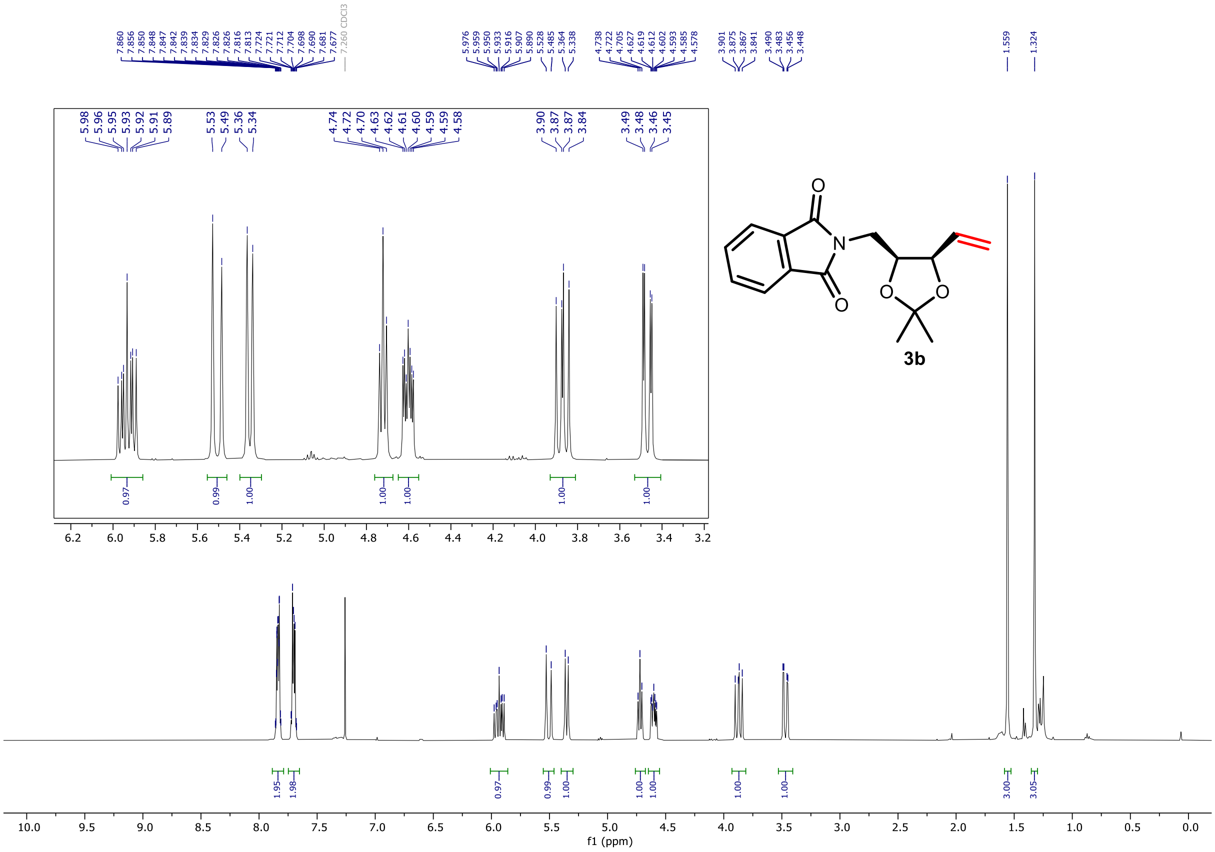

Figure S64. *400 MHz ^{1}H NMR of* ***3b***

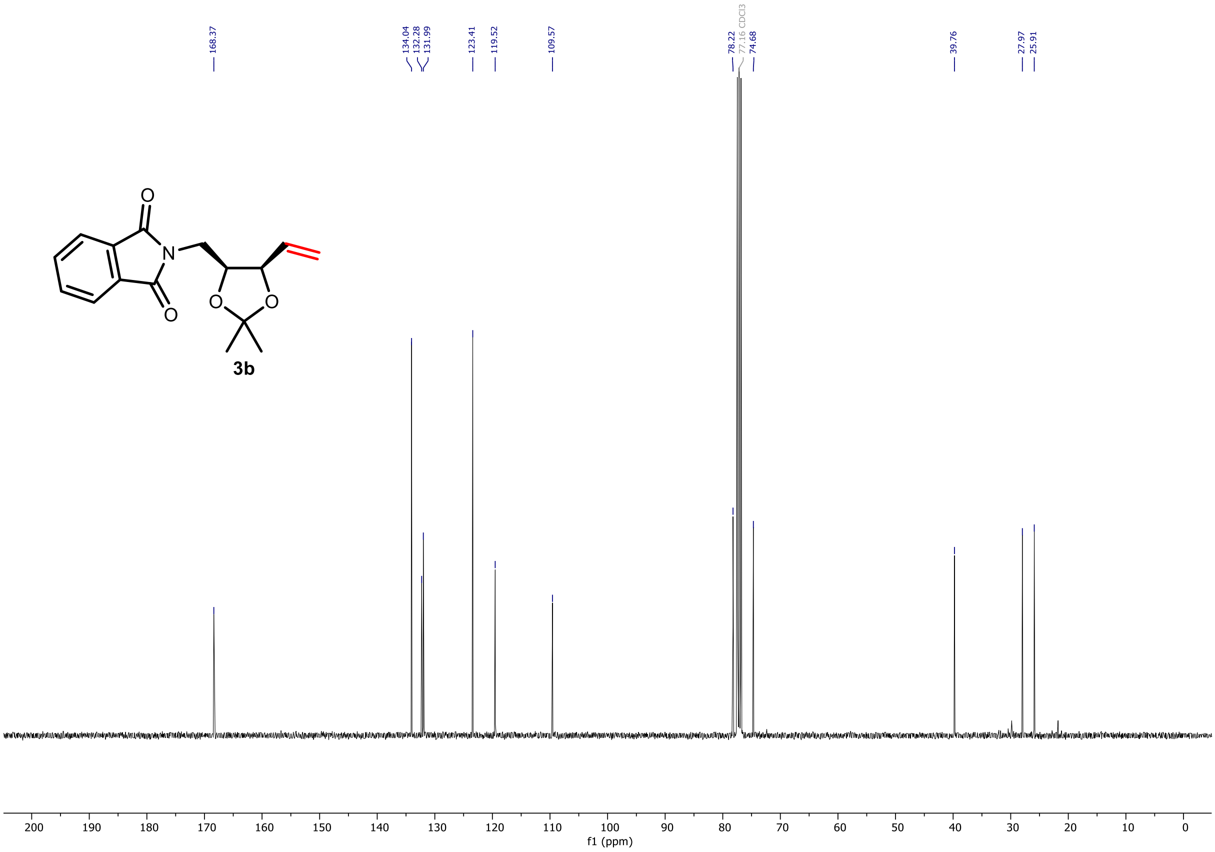

Figure S65. *101 MHz^{13}C NMR of* ***3b***

H.3.3. NMR of 1,2,5-trideoxy-3,4-*O*-(1-methylethylidene)-5-iodine-L-*erythro*-pent-1-enitol (3c)

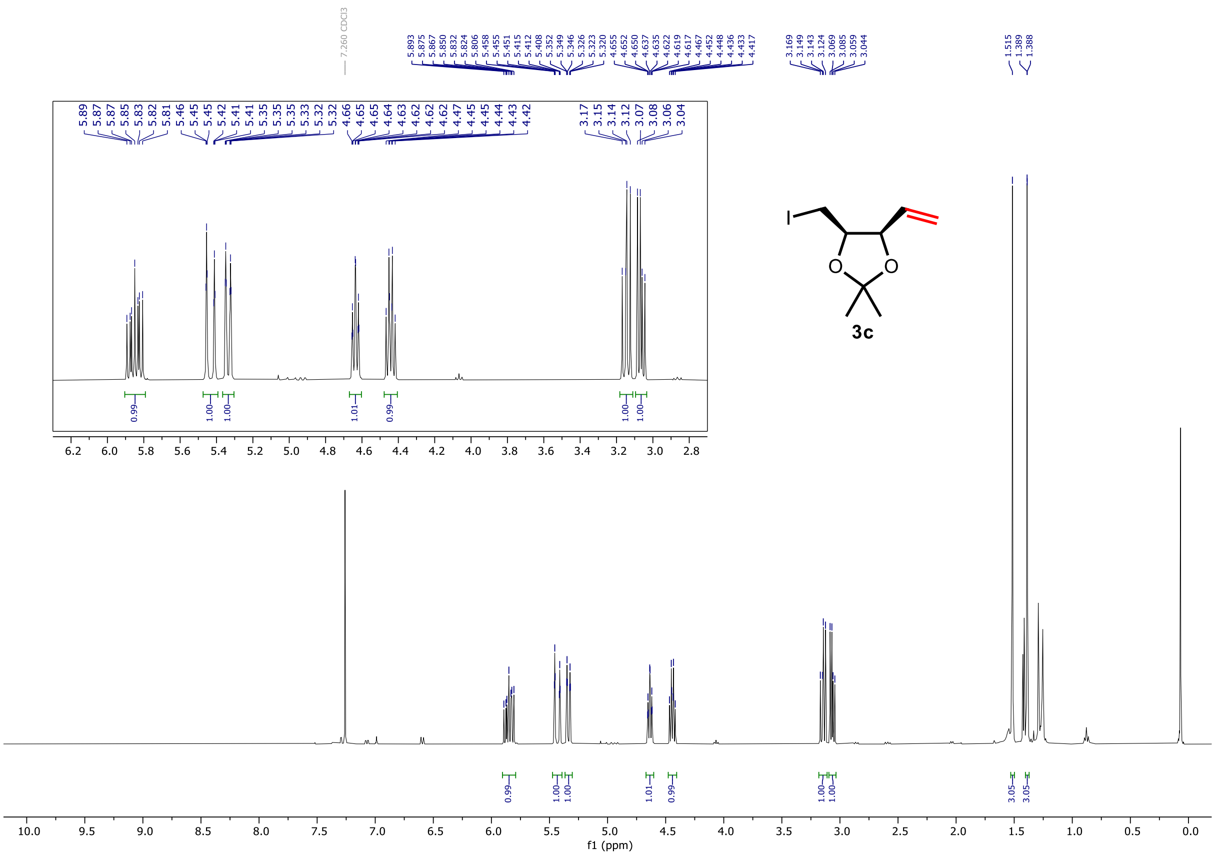

Figure S66. *400 MHz ^{1}H NMR of* ***3c***

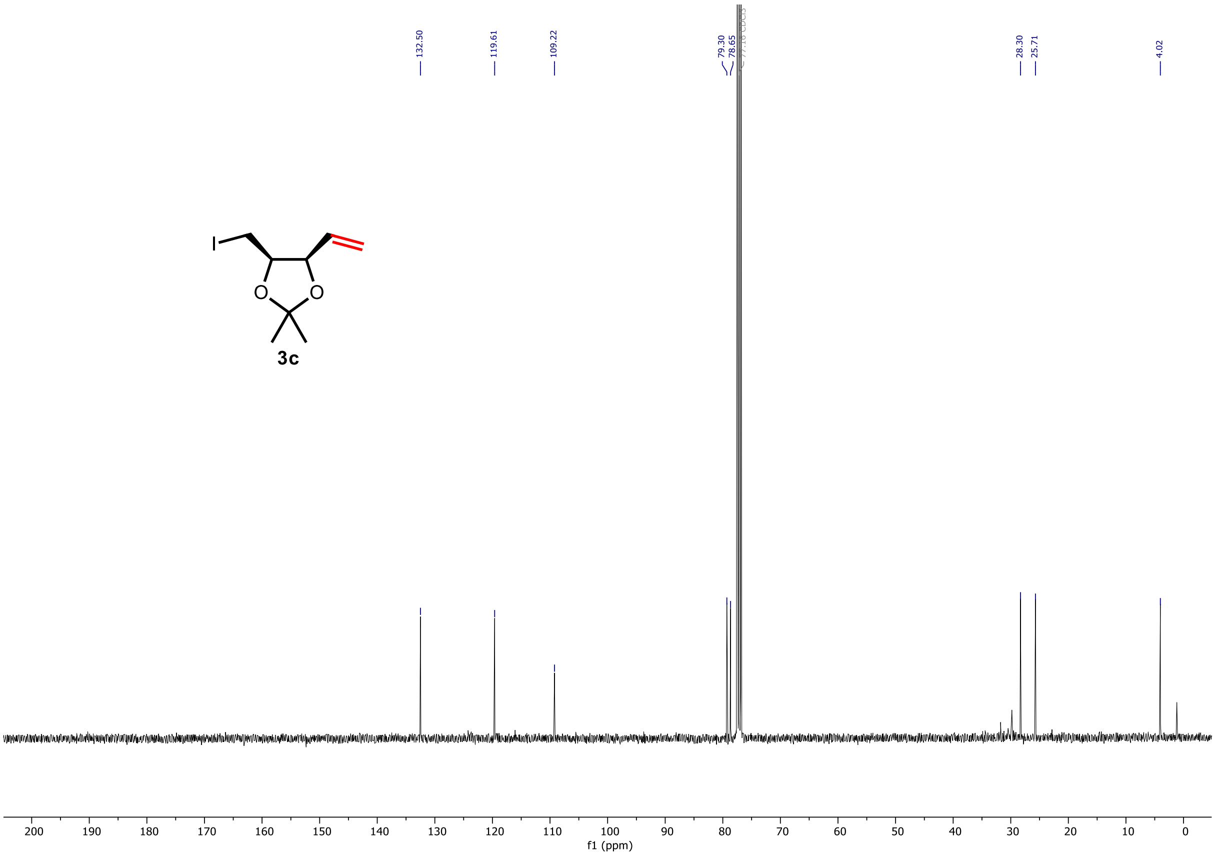

Figure S67. *101 MHz ^{13}C NMR of* ***3c***

H.4. Diene-Products

H.4.1. NMR of 1,2,4-trideoxy-3,5,7-tri-*O*-benzyl-D-*gluco*-hept-1,3-dienitol (2b')

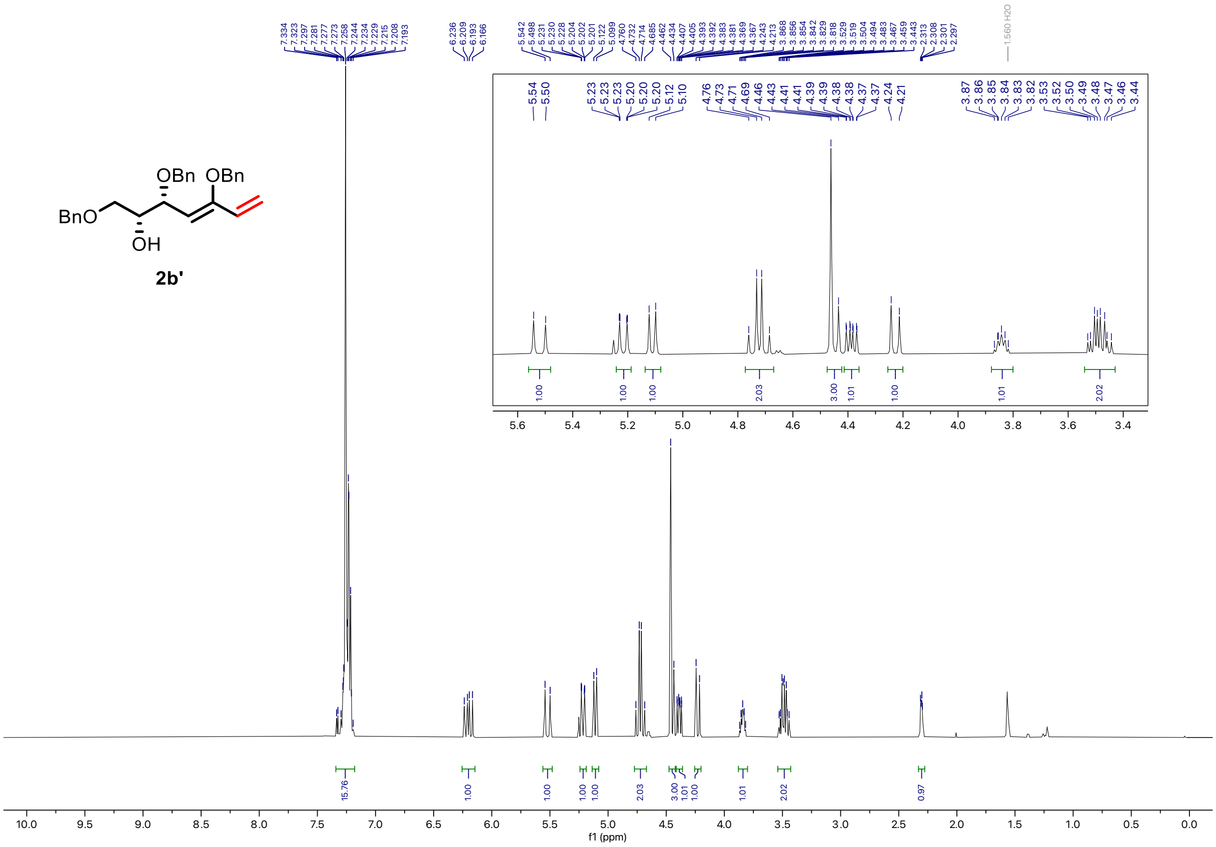

Figure S68. *400 MHz ^1H NMR of **2b'***

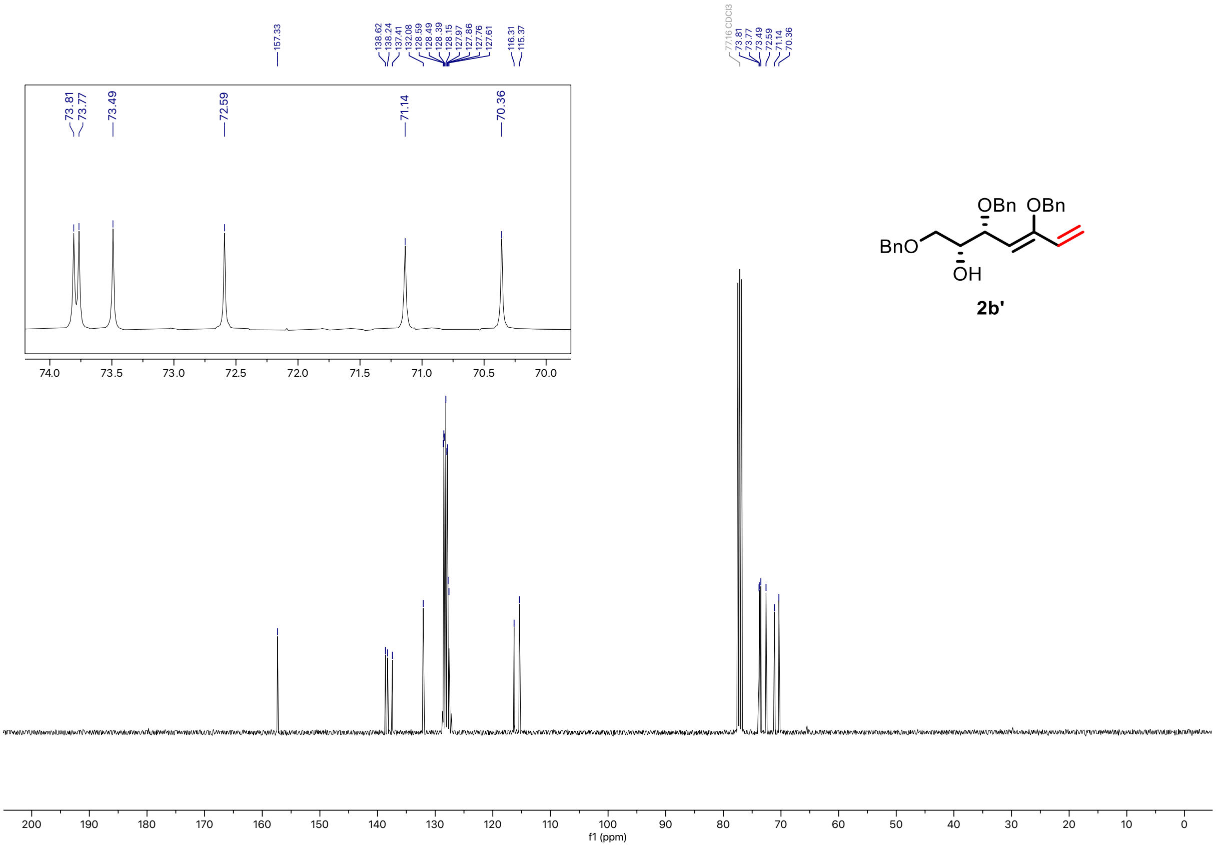

Figure S69. *101 MHz ^{13}C NMR of **2b'***

H.4.2. NMR of 1,2,4-trideoxy-3,5-di-*O*-benzyl-D-*xylo*-hex-1,3-dienitol (2f')

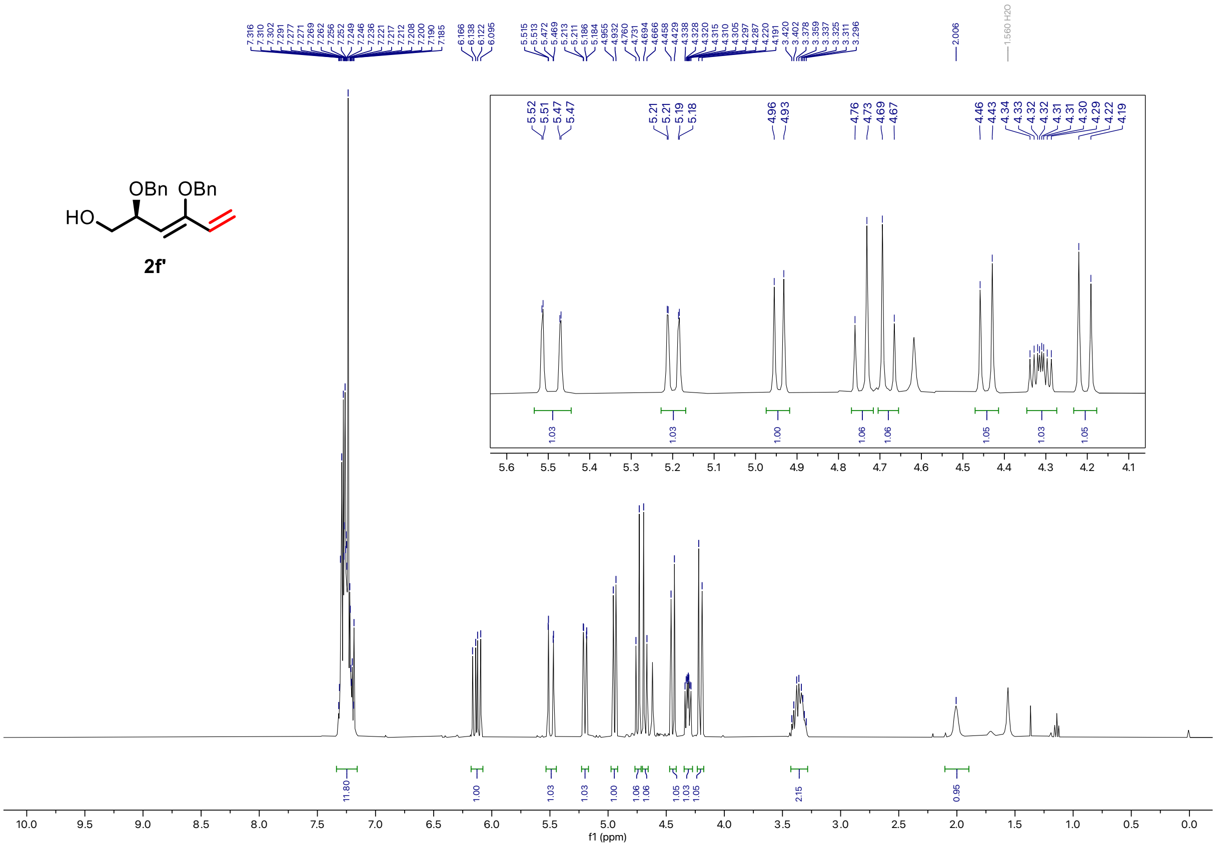

Figure S70. 400 MHz ^{1}H NMR of **2f'**

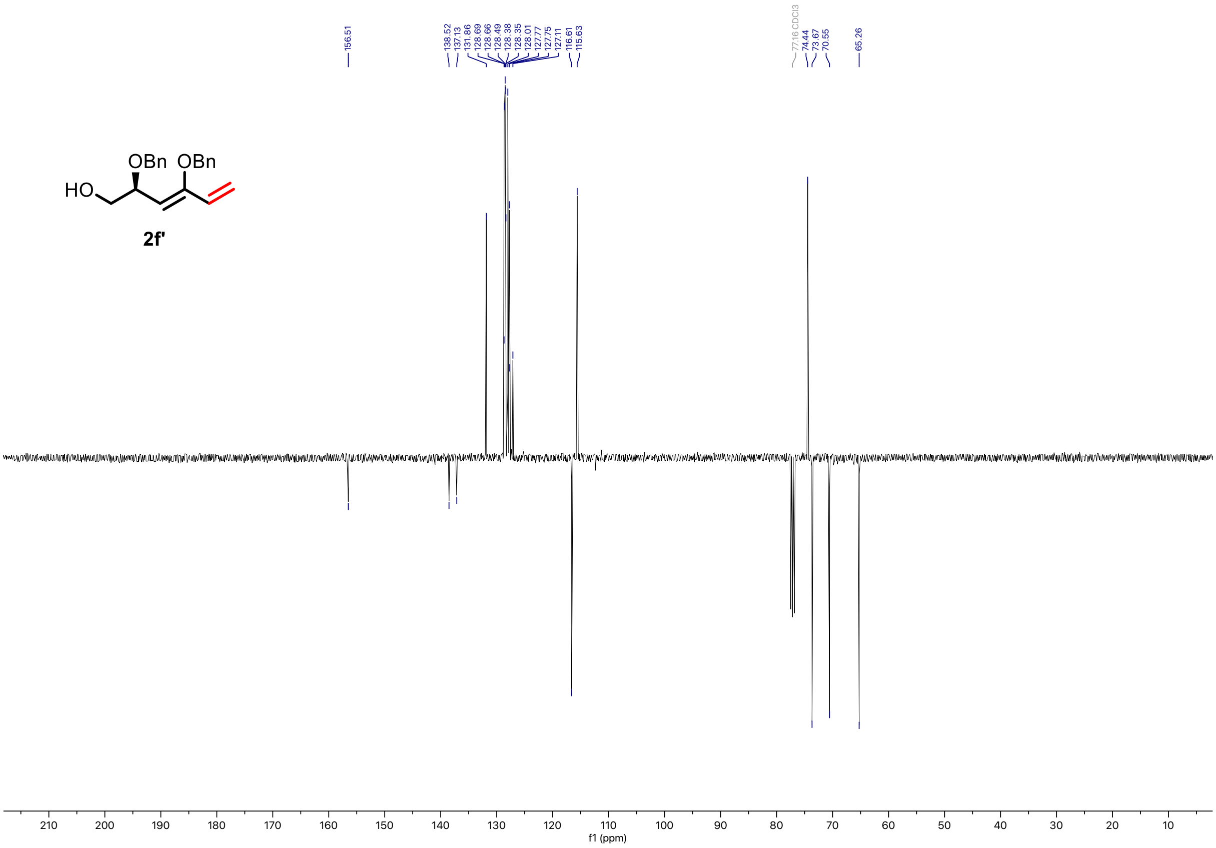

Figure S71. 101 MHz ^{13}C NMR of **2f'**
